# Supplementary material for: Deregulations of miR‐1 and its target Multiplexin promote dilated cardiomyopathy associated with myotonic dystrophy type 1
Source: EMBO Rep. 2023 Feb 28;24(4):e56616. doi: 10.15252/embr.202256616 (PMC10074075; doi:10.15252/embr.202256616)
Supplement: Supplementary file 1 — Expanded View Figures PDF [file EMBR-24-e56616-s002.pdf]

Expanded View Figures

Figure EV1. Flies showing DCM present myofibrillar disarray in the heart tubes.

A–D′ Z stack projection from hearts of controls (*UAS-mblRNAi*, *UAS-Bru3*, *UAS-dmiR-1 sponge* and *UAS-Mp*) (A–D) and mutants (*Hand* > *mblRNAi*, *Hand* > *Bru3*, *Hand* > *dmiR-1 sponge* and *Hand* > *Mp*) (A′–D′) aged of 5 weeks. The arrows point to irregularity in myofibrillar density and arrangement in the heart tubes. Scale bar = 20 μm.

E–G Box plots showing cardiac size analyses (diastolic (E) and systolic (F) diameters) and percent fractional shortening (G) performed by SOHA approach for controls (*Hand-Gal4* and *UAS-960CTG*) and mutant (*Hand* > *960CTG*) at 1 and 5 weeks of age. Aged *Hand* > *960CTG* flies present cardiac dilation characterized by a significant increase in diastolic and systolic diameters in comparison with control *Hand* > *Gal4* (E, F). *n* = 20 hearts. Central band corresponds to median. Whiskers correspond to Min to Max. Box corresponds to interquartile range from 25th to 75th percentile.

Data information: *P*-value < 0.05 considered statistically significant. (**) *P*-value = 0.021, (***) *P*-value = 0.0002. The nonparametric Mann–Whitney test was performed to compare control samples and samples of interest.

© 2023 The Authors

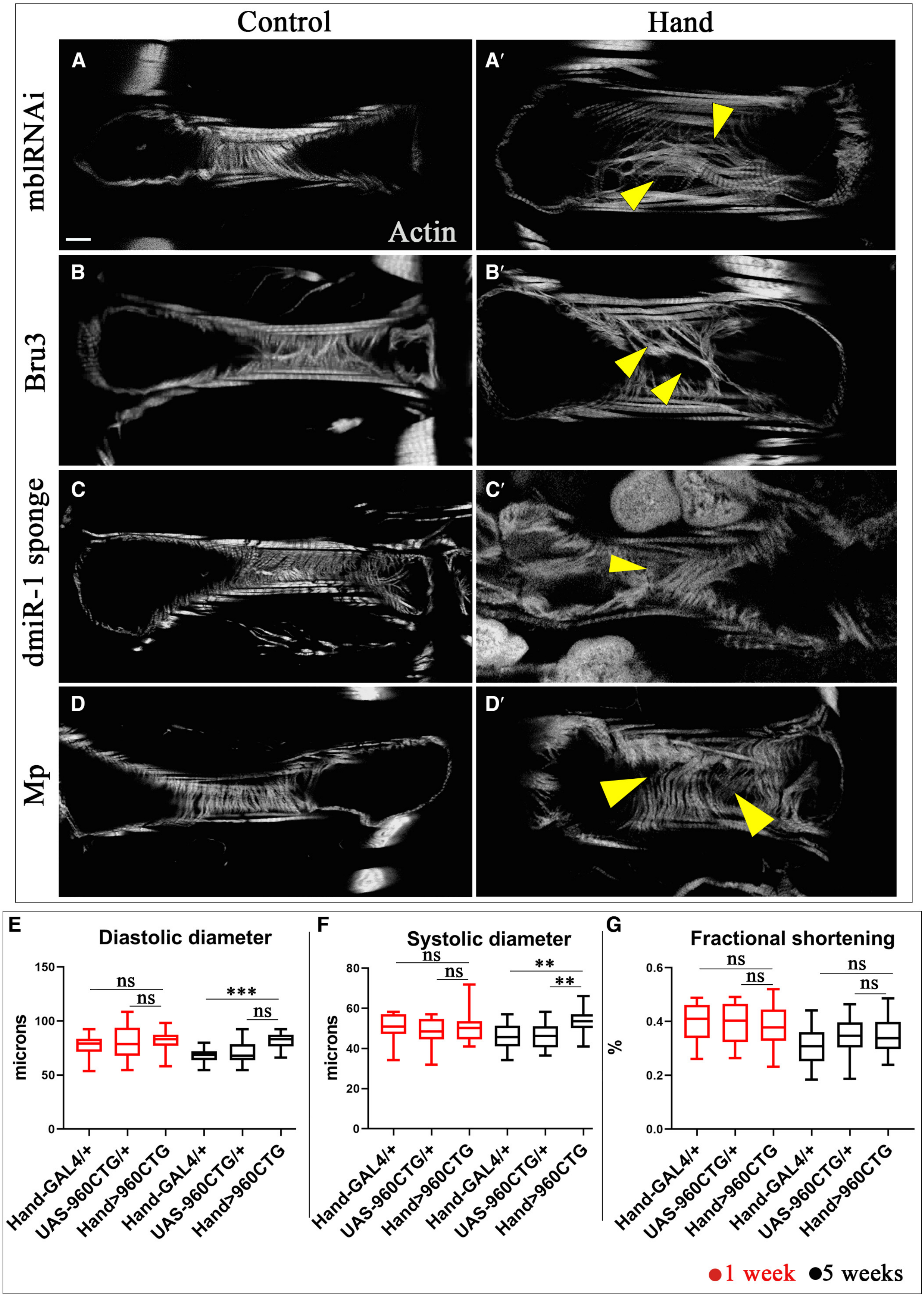

Figure EV1.

 © 2023 The Authors

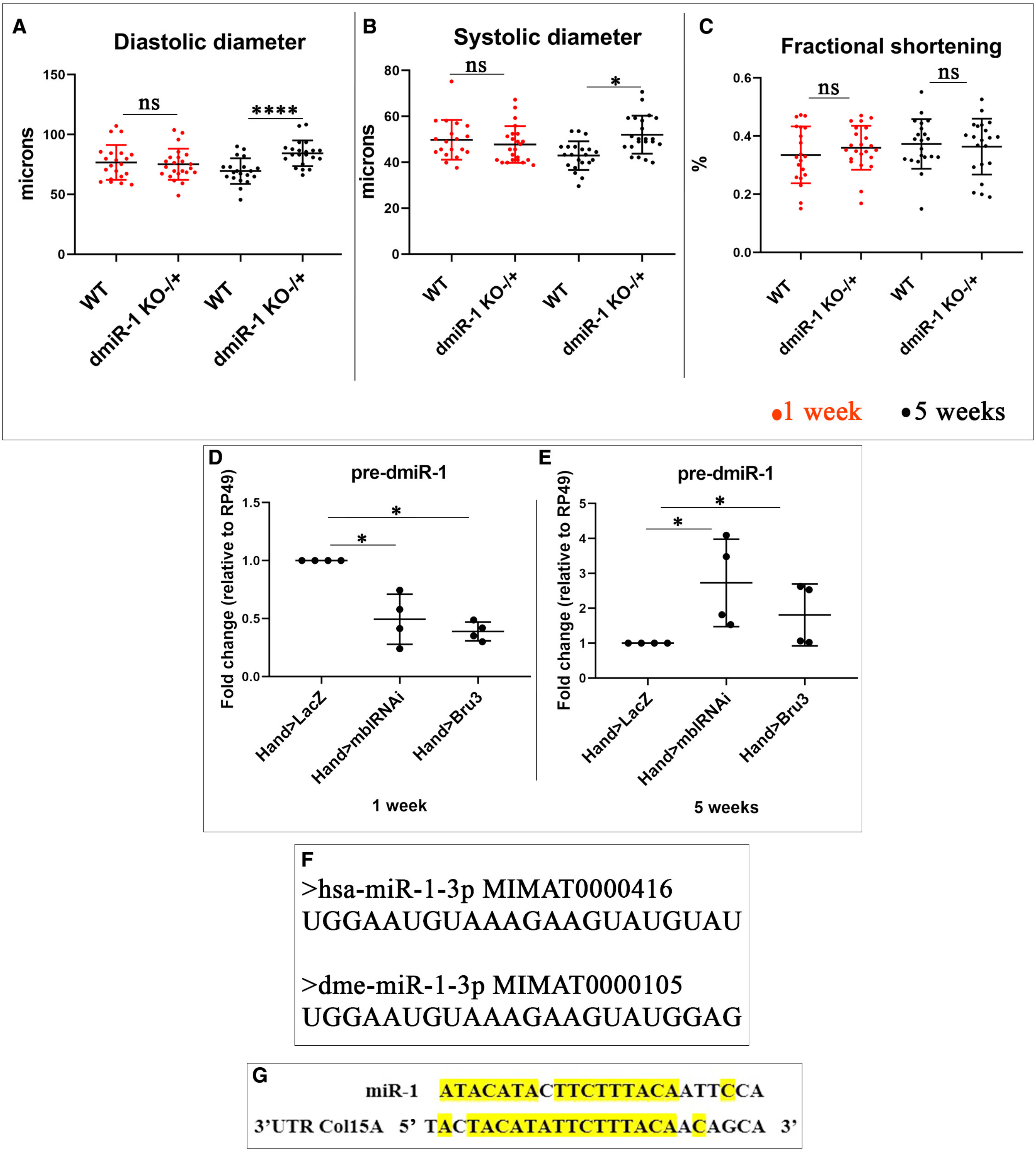
A
Diastolic diameter
microns
150
100
50
0
ns
****
WT
dmiR-1 KO-/+
WT
dmiR-1 KO-/+
B
Systolic diameter
microns
80
60
40
20
0
ns
*
WT
dmiR-1 KO-/+
WT
dmiR-1 KO-/+
C
Fractional shortening
%
0.6
0.4
0.2
0.0
ns
ns
WT
dmiR-1 KO-/+
WT
dmiR-1 KO-/+
•1 week
•5 weeks
D
pre-dmiR-1
Fold change (relative to RP49)
1.5
1.0
0.5
0.0
*
*
Hand>LacZ
Hand>mblRNAi
Hand>Bru3
1 week
E
pre-dmiR-1
Fold change (relative to RP49)
5
4
3
2
1
0
*
*
Hand>LacZ
Hand>mblRNAi
Hand>Bru3
5 weeks
F
>hsa-miR-1-3p MIMAT0000416
UGGAAUGUAAAGAAGUAUGUAU
>dme-miR-1-3p MIMAT0000105
UGGAAUGUAAAGAAGUAUGGAG
G
miR-1 ATACATACTTCTTTACAATTCCA
3'UTR Col15A 5' TACTACATATTCTTTACAACAGCA 3'

Figure EV2.

Figure EV2. Supplemental analyses of *dmiR-1*.

A–C Scatter plot showing cardiac size analyses (diastolic (A) and systolic (B) diameters) and percent fractional shortening (C) performed by SOHA approach for control wt^{1118} and heterozygous mutant context (*dmiR-1 KO*$^{-/+}$) at 1 and 5 weeks of age. Aged *Hand* > *dmiR-1 KO*$^{-/+}$ flies present cardiac dilation characterized by a significant increase in diastolic and systolic diameters in comparison with control. $n = 20$ hearts. Bars correspond to mean ± SD.

D, E RTq–PCR analysis for *pre-dmiR-1* transcript in adult heart of 1 and 5 weeks of age for control (*Hand* > *LacZ*) and DM1 contexts (*Hand* > *mblRNAi*, *Hand* > *Bru3*). $n = 4$ biological replicates.

F Alignment of *Drosophila* and human *miR-1* sequences.

G Potential binding site of human *miR-1* in 3′UTR of *COL15A1* (*COL15A1–201* transcript position 4428 to 4447).

Data information: *P*-value < 0.05 considered statistically significant. (*) *P*-value = 0.033, (****) *P*-value < 0.0001. The nonparametric Mann–Whitney test was performed to compare control samples and samples of interest.

Figure EV3. DM1 flies show reduced levels of *dmiR-1* in cardiac cells.

A–H′ FISH for scramble (5′ 3′ DIG) (A′) and U6 (5′DIG) (B′) detected in ω^{1118} cardiac tube. FISH with miRCURY LNA probe for *dmiR-1* detection in controls (*UAS-dmiR-1*, *UAS-mblRNAi*, *UAS-Bru3*) (C′, E′, G′) and mutants (*Hand* > *dmiR-1*, *Hand* > *mblRNAi*, *Hand* > *Bru3*) (D′, F′, H′) cardiac tubes labeled with *dmiR-1* probes (red) and actin (green) at 1 week of age. Representative spot views generated using Imaris from *in situ* hybridization with miRCURY LNA probe for *dmiR-1* and used for quantification of *dmiR-1* levels.

I, I′ Spot views of *dmiR-1* in hearts of 1-week-old control (*UAS-mblRNAi*) (I) and DM1 (*Hand* > *mblRNAi*) flies (I′) are shown. Each spot represents a pool of *dmiR-1* transcripts detected in the same area. The zoom area in (I) and (I′) corresponds to pericardial cells.

J, K Scatter plot graph showing the signal intensity of *dmiR-1* levels quantified in pericardial cells of 1- and 5-week-old flies for controls (*UAS-Bru3*, *UAS-mblRNAi*) and DM1 contexts (*Hand* > *Bru3*, *Hand* > *mblRNAi*). $n = 27$ pericardial cells. Bars correspond to mean ± SD.

Data information: Scale bar = 40 μm. *P*-value < 0.05 considered statistically significant. (*) *P*-value = 0.033, (**) *P*-value = 0.021, (***) *P*-value = 0.0002, (****) *P*-value < 0.0001. The nonparametric Mann–Whitney test was performed to compare control samples and samples of interest.

© 2023 The Authors

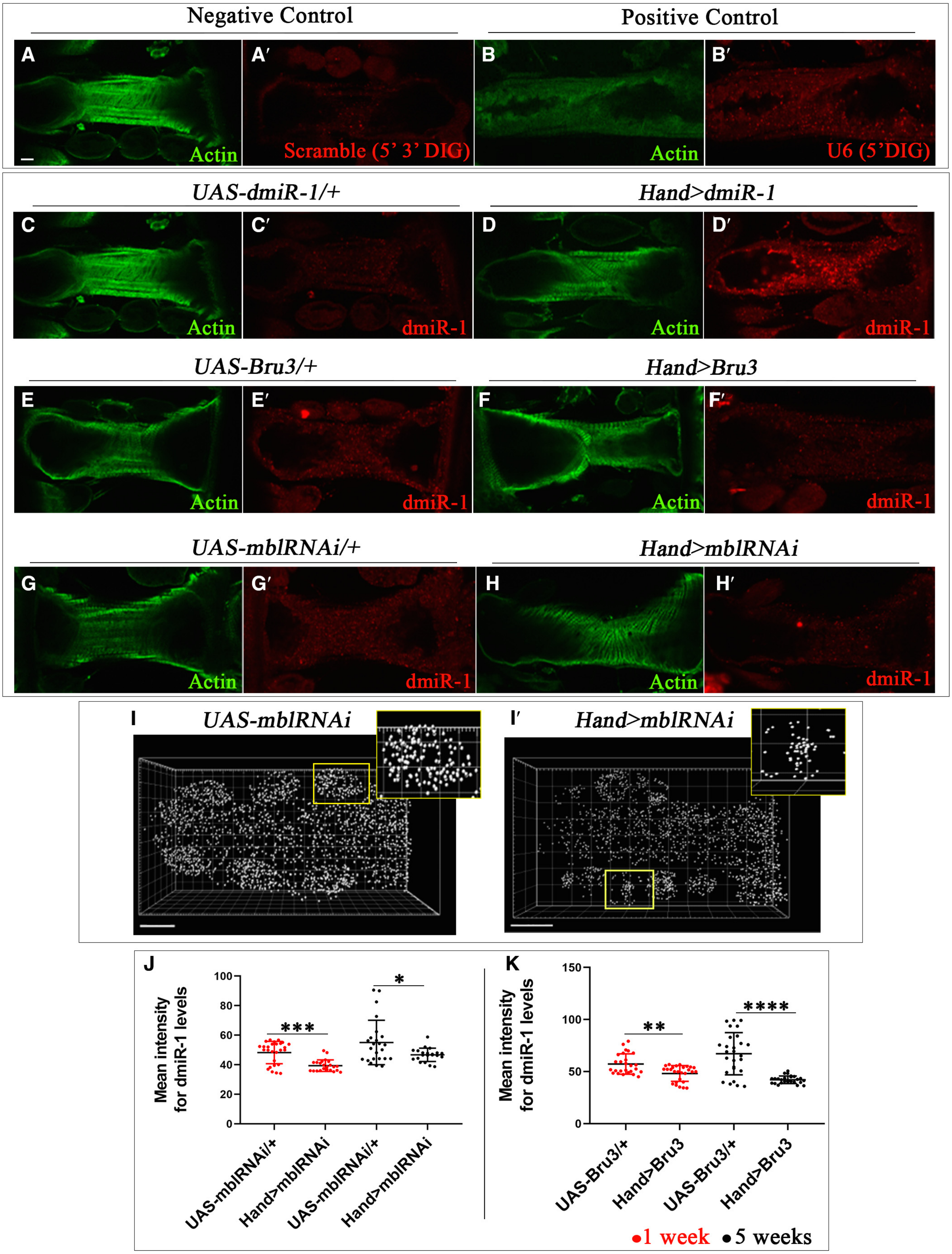
Negative Control
Positive Control
A
A′
B
B′
Actin
Scramble (5' 3' DIG)
Actin
U6 (5'DIG)
UAS-dmiR-1/+
Hand>dmiR-1
C
C′
D
D′
Actin
dmiR-1
Actin
dmiR-1
UAS-Bru3/+
Hand>Bru3
E
E′
F
F′
Actin
dmiR-1
Actin
dmiR-1
UAS-mblRNAi/+
Hand>mblRNAi
G
G′
H
H′
Actin
dmiR-1
Actin
dmiR-1
I
UAS-mblRNAi
I′
Hand>mblRNAi
J
Mean intensity for dmiR-1 levels
100
80
60
40
20
0
***
*
UAS-mblRNAi/+
Hand>mblRNAi
UAS-mblRNAi/+
Hand>mblRNAi
K
Mean intensity for dmiR-1 levels
150
100
50
0
**
****
UAS-Bru3/+
Hand>Bru3
UAS-Bru3/+
Hand>Bru3
1 week
5 weeks

Figure EV3.

Figure EV4. Mp is expressed in the adult fly heart and up-regulated in pericardial cells of DCM-developing DM1 lines.

A–C″ (A) Adult heart of ω^{1118} line labeled for Mp (green), actin (red), and DAPI (blue). (C) The yellow arrows indicate ventral longitudinal muscles (VLM). (C′) The yellow arrows indicate circular fibers (within cardioblast cells). (A″, B″) Cross-section of ω^{1118} cardiac tube after 3D reconstruction with Imaris software, showing the expression of Mp at the internal and the external surface of the cardiac tube.

D–G′ Adult heart of 1 and 5 weeks of age labeled for Mp (green) for controls (*UAS-mblRNAi*, *UAS-Bru3*) (D–G) and DM1 context (*Hand > mblRNAi*, *Hand > Bru3*) (D′–G′). Encircled region in E′ corresponds to an example of area used for quantifications of the fluorescent signal in pericardial cells using the CTCF method.

H, I Fluorescence signal intensity quantification for Mp expression in pericardial cells in adult heart of 1 and 5 weeks of age for controls (*UAS-mblRNAi*, *UAS-Bru3*) and DM1 context (*Hand > mblRNAi*, *Hand > Bru3*) using the CTCF method. For each genotype, 9 hearts were analyzed and 3 pericardial cells were analyzed from each heart. Bars correspond to mean ± SD.

J Fluorescence signal intensity quantification for Mp expression in cardioblasts in adult heart of 1 week of age for control (*UAS-dmiR-1 sponge*) and mutant (*Hand > dmiR-1 sponge*) using the CTCF method. For each genotype, 9 hearts were analyzed and fluorescence intensity was measured in two regions from segment A3 and two regions from segment A4. Bars correspond to mean ± SD.

Data information: Scale bar = 20 μm. *P*-value < 0.05 considered statistically significant. (**) *P*-value = 0.021, (***) *P*-value = 0.0002, (****) *P*-value < 0.0001. The nonparametric Mann–Whitney test was performed to compare control samples and samples of interest.

 © 2023 The Authors

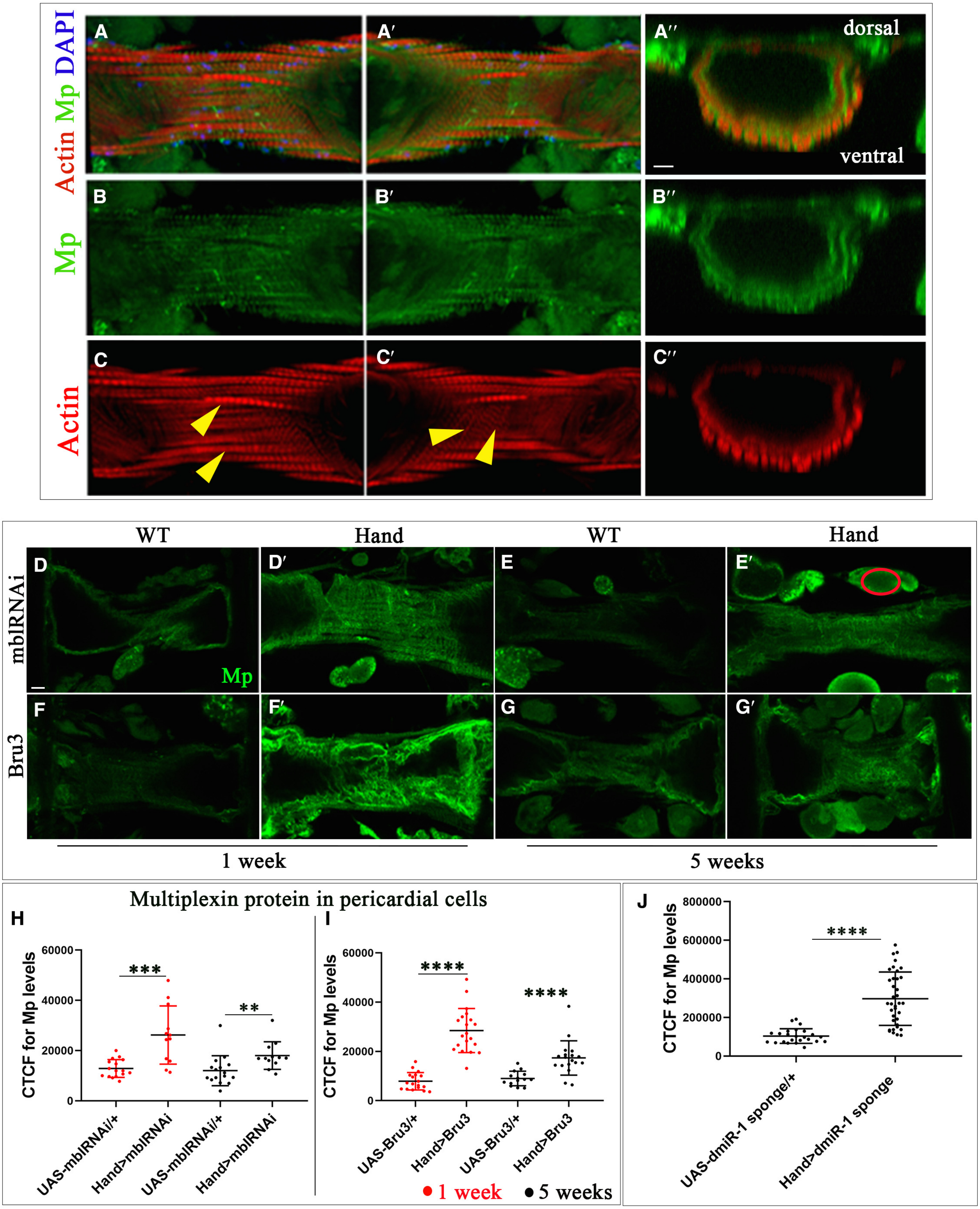
Actin Mp DAPI
A
A′
A″
dorsal
ventral
Mp
B
B′
B″
Actin
C
C′
C″
WT
Hand
WT
Hand
mblRNAi
D
D′
E
E′
Mp
Bru3
F
F′
G
G′
1 week
5 weeks
Multiplexin protein in pericardial cells
H
CTCF for Mp levels
60000
40000
20000
0
***
**
UAS-mblRNAi/+
Hand>mblRNAi
UAS-mblRNAi/+
Hand>mblRNAi
I
CTCF for Mp levels
60000
40000
20000
0
****
****
UAS-Bru3/+
Hand>Bru3
UAS-Bru3/+
Hand>Bru3
1 week
5 weeks
J
CTCF for Mp levels
800000
600000
400000
200000
0
****
UAS-dmiR-1 sponge/+
Hand>dmiR-1 sponge

Figure EV4.

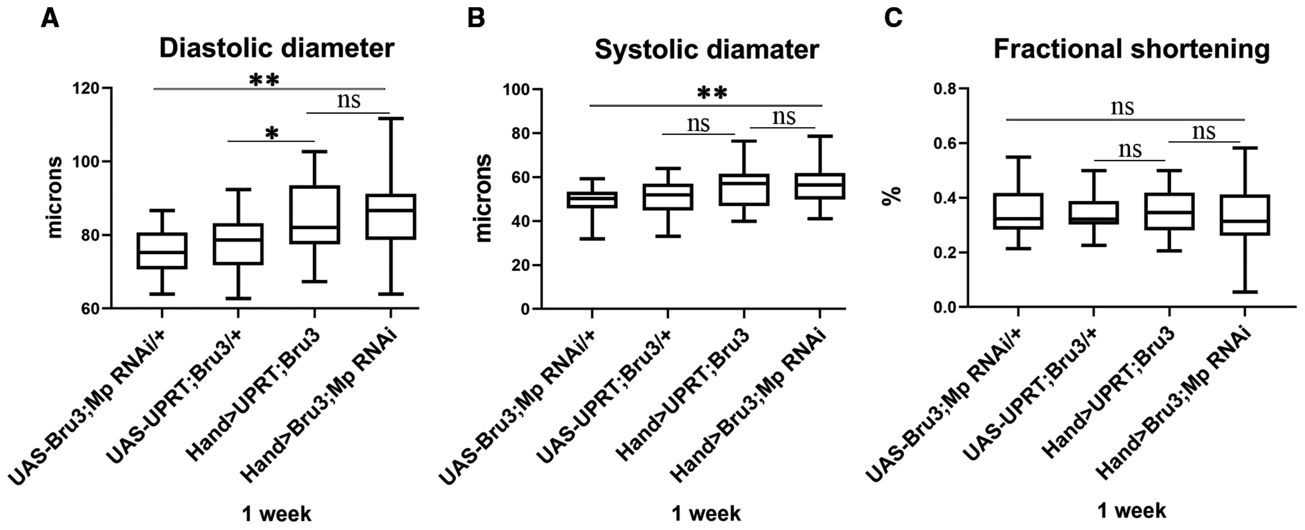

Figure EV5. The young DM1 flies with reduced Mp present normal heart size.

A–C Box plots showing cardiac size analyses (diastolic (A) and systolic (B) diameters) and percent fractional shortening (C) performed by SOHA approach for controls (*UAS-Bru3; UAS-MpRNAi* and *UAS-UPRT; Bru3*) and Mp rescue (*Hand > Bru3; Mp RNAi*) and DM1 (*Hand > UPRT; Bru3*) at 1 week of age. $n = 20$ hearts. Central band corresponds to median. Whiskers correspond to Min to Max. Box corresponds to interquartile range from 25th to 75th percentile.

Data information: P-value < 0.05 considered statistically significant. (*) P-value = 0.033, (**) P-value = 0.021. The nonparametric Mann–Whitney test was performed to compare control samples and samples of interest.

 © 2023 The Authors
