# Supplementary material for: Deregulations of miR‐1 and its target Multiplexin promote dilated cardiomyopathy associated with myotonic dystrophy type 1
Source: EMBO Rep. 2023 Feb 28;24(4):e56616. doi: 10.15252/embr.202256616 (PMC10074075; doi:10.15252/embr.202256616)
Supplement: Supplementary file 3 — PDF+ [file EMBR-24-e56616-s007.pdf]

Article

SOURCE DATA · TRANSPARENT PROCESS · OPEN ACCESS

EMBO reports

Deregulations of miR-1 and its target Multiplexin promote dilated cardiomyopathy associated with myotonic dystrophy type 1

Anissa Souidi[1], Masayuki Nakamori[2], Monika Zmojdzian[1], Teresa Jagla[1], Yoan Renaud[1] & Krzysztof Jagla[1,*]

Abstract

Myotonic dystrophy type 1 (DM1) is the most common muscular dystrophy in adults. It is caused by the excessive expansion of non-coding CTG repeats, which when transcribed affects the functions of RNA-binding factors with adverse effects on alternative splicing, processing, and stability of a large set of muscular and cardiac transcripts. Among these effects, inefficient processing and down-regulation of muscle- and heart-specific miRNA, *miR-1*, have been reported in DM1 patients, but the impact of reduced *miR-1* on DM1 pathogenesis has been unknown. Here, we use *Drosophila* DM1 models to explore the role of *miR-1* in cardiac dysfunction in DM1. We show that *miR-1* down-regulation in the heart leads to dilated cardiomyopathy (DCM), a DM1-associated phenotype. We combined *in silico* screening for *miR-1* targets with transcriptional profiling of DM1 cardiac cells to identify *miR-1* target genes with potential roles in DCM. We identify Multiplexin (Mp) as a new cardiac *miR-1* target involved in DM1. *Mp* encodes a collagen protein involved in cardiac tube formation in *Drosophila*. Mp and its human ortholog Col15A1 are both highly enriched in cardiac cells of DCM-developing DM1 flies and in heart samples from DM1 patients with DCM, respectively. When overexpressed in the heart, Mp induces DCM, whereas its attenuation rescues the DCM phenotype of aged DM1 flies. Reduced levels of *miR-1* and consecutive up-regulation of its target Mp/Col15A1 might be critical in DM1-associated DCM.

Keywords dilated cardiomyopathy; *Drosophila*; *miR-1*; *Multiplexin*; myotonic dystrophy type 1
Subject Categories Cardiovascular System; Molecular Biology of Disease; Musculoskeletal System
DOI 10.15252/embr.202256616 | Received 6 December 2022 | Revised 3 February 2023 | Accepted 14 February 2023 | Published online 28 February 2023
EMBO Reports (2023) 24: e56616

Introduction

Myotonic dystrophy type 1 (DM1) is the most common muscular dystrophy in adults, with an estimated incidence of one in 8,000 births (Meola & Cardani, 2015). DM1 is a multisystem disorder, with cardiac abnormalities accounting for 30% of fatalities (Mathieu *et al*, 1999). Cardiac involvements in DM1 include conduction defects, supraventricular and ventricular arrhythmias (Pelargonio *et al*, 2002), impaired diastolic or systolic function (Pelargonio *et al*, 2002; Hermans *et al*, 2012), and dilated cardiomyopathy (DCM) (Nguyen *et al*, 1988; Lin *et al*, 1989; Hermans *et al*, 2012; Schilling *et al*, 2013; Papa *et al*, 2018).

The primary cause of DM1 is the gain-of-function of toxic transcripts carrying expanded noncoding CUG repeats that aggregate into foci in nuclei, sequestering RNA-binding protein Muscleblind-like 1 (MBNL1) (Fardaei *et al*, 2001, 2002). Reduced MBNL1 levels and concomitant stabilization of another RNA-binding protein, Elav-like family member 1 (CELF1) (Kuyumcu-Martinez *et al*, 2007), lead to the deregulation of alternative splicing with the abnormal expression of embryonic splice variants in adult tissues (Lee & Cooper, 2009). For example, the CELF1-dependent inclusion of fetal exon 5 in the adult isoform of *cardiac troponin T* (*cTnT*) has been associated with cardiac conduction defects in DM1 (Philips *et al*, 1998). Besides their roles as alternative splicing regulators, both MBNL1 and CELF1 are involved in mRNA translation (Timchenko *et al*, 2005, 2006; Dasgupta & Ladd, 2012; de Haro *et al*, 2013), deadenylation and decay (Vlasova *et al*, 2008; Dasgupta & Ladd, 2012; Masuda *et al*, 2012), and in RNA editing (Dasgupta & Ladd, 2012). MBNL1 is specifically involved in miRNA processing (Rau *et al*, 2011). These diverse functions of MBNL1 and CELF1 mean that DM1 may involve the deregulation of multiple pathways.

To investigate the pathophysiology and the molecular mechanisms underlying DM1, several DM1 models, both mouse (Wang *et al*, 2007; Orengo *et al*, 2008; Huguet *et al*, 2012) and *Drosophila* (Houseley *et al*, 2005; de Haro *et al*, 2006; Garcia-Lopez *et al*, 2008; Picchio *et al*, 2013), have been created.

1 iGReD Genetics Reproduction and Development Institute, Clermont Auvergne University, Clermont-Ferrand, France
2 Department of Neurology, Osaka University Graduate School of Medicine, Suita, Japan
*Corresponding author. Tel: +33 473178181; E-mail: christophe.jagla@uca.fr

© 2023 The Authors. Published under the terms of the CC BY 4.0 license.

The reduction in MBNL1 and stabilization of CELF1 are thought to be involved in most DM1 phenotypes. Indeed, *Mbnl1* knockout mice develop muscle myotonia, weakness/wasting, and cardiac defects including dilated cardiomyopathy and heart conduction block (Lee *et al*, 2013). Mice overexpressing *CELF1* in the heart show conduction abnormalities and dilated cardiomyopathy (Koshelev *et al*, 2010) thus confirming the contribution of MBNL1 sequestration and CELF1 up-regulation to DM1 pathogenesis. Overall, the mouse models reproduced multiple DM1 features including RNA foci formation and various alternative splice defects.

We generated a series of inducible *Drosophila* DM1 lines bearing UAS-iCTG constructs with 240, 480, 600, and 960 CTGs (Picchio *et al*, 2013). These lines were used to model DM1 in larval somatic muscles showing not only nuclear foci formation and Mbl sequestration but also muscle hypercontraction, splitting of muscle fibers, reduced fiber size, and myoblast fusion defects leading to impaired larva mobility (Picchio *et al*, 2013). The severity of phenotypes in these *Drosophila* models could be correlated with repeat size (Picchio *et al*, 2013), as also observed in DM1 patients. Finally, the overexpression of *Drosophila CELF1* ortholog *Bru3* and attenuation of *MBNL1* counterpart *mbl* (Picchio *et al*, 2018; Auxerre-Plantié *et al*, 2019) offer further valuable models for identifying gene deregulations underlying DM1.

Among molecular mechanisms associated with DM1, the deregulation of miRNAs and in particular reduced levels of evolutionarily conserved muscle- and heart-specific miRNA, *miR-1*, has been reported in DM1 patients (Rau *et al*, 2011) and in DM1 models including mouse (Kalsotra *et al*, 2014) and *Drosophila* (Fernandez-Costa *et al*, 2013). However, the impact of *miR-1* down-regulation on DM1-associated phenotypes has not yet been analyzed.

Here, we made use of *Drosophila* DM1 models to explore *miR-1* involvement in cardiac dysfunction in DM1. We observed that *dmiR-1* level was reduced in the cardiac cells of DM1 flies and that its down-regulation in the heart led to DCM, thus suggesting that reduced *dmiR-1* levels contribute to DM1-associated DCM. Among potential *dmiR-1* regulated genes from *in silico* screening, we identified *Multiplexin* (*Mp*)/*Collagen15A1* (*Col15A1*) as a new cardiac *dmiR-1* target involved in DM1. Both Mp and Col15A1 proteins were highly enriched in cardiac cells of DCM-developing DM1 flies and in heart samples from DM1 patients with DCM, respectively. Moreover, the heart-targeted overexpression of Mp was sufficient to induce DCM, whereas its attenuation rescues the DCM phenotype in DM1 flies. *miR-1* and its target *Mp/Col15A1* thus emerge as molecular determinants of DM1-associated DCM.

Results

Heart-targeted *dmiR-1* attenuation causes DCM in *Drosophila*

Reduced *miR-1* levels had previously been observed in mice developing DCM (Isserlin *et al*, 2014) and in cardiac samples from patients with end-stage DCM (Ikeda *et al*, 2007). It had also been shown that *miR-1* knockout mice display the DCM phenotype (Wei *et al*, 2014). However, whether *miR-1* attenuation specifically within the heart leads to DCM has not been assessed. Here we tested the heart-specific knockdown (KD) of *dmiR-1* in *Drosophila* using a sponge line that contains multiple complementary binding sites for *dmiR-1*. When expressed at high levels under the control of a cardiac-specific *Hand-Gal4* driver, sponge sequences inhibit the activity of *dmiR-1* in fly heart, thus preventing its function (Fulga *et al*, 2015). In the *Hand* > *dmiR-1 sponge* context, the adult fly hearts showed a larger diameter with an enlarged cardiac lumen (Fig 1B and B′) in comparison to the control (Fig 1A and A′). Consistent with this observation, analyses of semi-intact *Hand* > *dmiR-1 sponge* heart preparations and generated M-modes (Fig 1C and C′) confirmed enlargement of the cardiac tube and showed significantly increased diastolic and systolic heart diameters in young and aged flies (Fig 1D and E) concomitant with myofibrillar disarray in the heart tube (Fig EV1C and C′). We then characterized the effects of *dmiR-1* down-regulation on heart contractility by assessing percent fractional shortening (%FS), which refers to the size of the cardiac tube at the end of systole and the end of diastole. The cardiac dilation in *Hand* > *dmiR-1 sponge* flies was associated with a significant reduction in heart contractility at both 1 and 5 weeks of age (Fig 1F). Sponge-driven *dmiR-1* attenuation in the *Drosophila* heart thus leads to DCM. This finding is supported by significantly increased diastolic and systolic heart diameters observed in heterozygous *dmiR-1 KO* −/+ flies at 5 weeks of age (Fig EV2A–C).

DM1 flies develop a DCM phenotype and show a reduced *dmiR-1* level in cardiac cells

DCM accounts for fatal cardiac involvements in DM1 patients, but the gene deregulations underlying DM1-associated DCM have not been identified. To address this issue, we first tested whether *Drosophila* DM1 models developed DCM. We tested three heart-specific DM1 contexts, namely (i) overexpression of 960CTG repeats, (ii) overexpression of *CELF1* ortholog *Bru3*, and (iii) attenuation of *MBNL1* ortholog *mbl*. Since the severity of cardiac phenotypes in DM1 increases with age, we performed all analyses at 1 and 5 weeks of age. From the three heart-specific DM1 contexts the DCM phenotype was present in *Hand* > *Bru3* and *Hand* > *mblRNAi* models at both 1 and 5 weeks of age (Fig 2A–F) but not in *Hand* > *960CTG* (Fig EV1E–G). The DCM phenotype was also associated with myofibrillar disarray in the *Hand* > *mblRNAi* and *Hand* > *Bru3* heart tubes (Fig EV1A′ and B′) in comparison to the controls (Fig EV1A and B). The *Hand* > *960CTG* line presents cardiac dilation at 5 weeks of age characterized by a significant increase in diastolic and systolic diameters (Fig EV1E and F). Nonaffected contractility in these DM1 models (Fig EV1G) could be due to a milder effect of 960CTG repeats on Bru3 and mbl levels compared with GAL4-driven overexpression of Bru3 and RNAi-knockdown of *mbl*.

Given that *dmiR-1* attenuation leads to DCM and that *Hand* > *Bru3* and *Hand* > *mblRNAi* DM1 models develop a DCM phenotype, we tested whether cardiac cells of *Hand* > *Bru3* and *Hand* > *mblRNAi* flies showed reduced *dmiR-1* levels. Using *in situ* hybridization (ISH) with *dmiR-1*-specific miRCURY LNA probe we found a significantly reduced *dmiR-1* level in cardiac cells of both DCM-developing DM1 contexts (Figs 2G, G′, H, and I, and EV3A–K). *Pre-dmiR-1* expression was also lower in young DM1 flies (Fig EV2D), whereas hearts from aged *Hand* > *mblRNAi* flies showed an increased *pre-dmiR-1* level, most probably owing to its impaired processing (Fig EV2E). DCM-developing *Drosophila* DM1 models could thus serve to test and identify genes deregulated in the DM1-associated DCM context in response to a reduced *dmiR-1* level.

 © 2023 The Authors

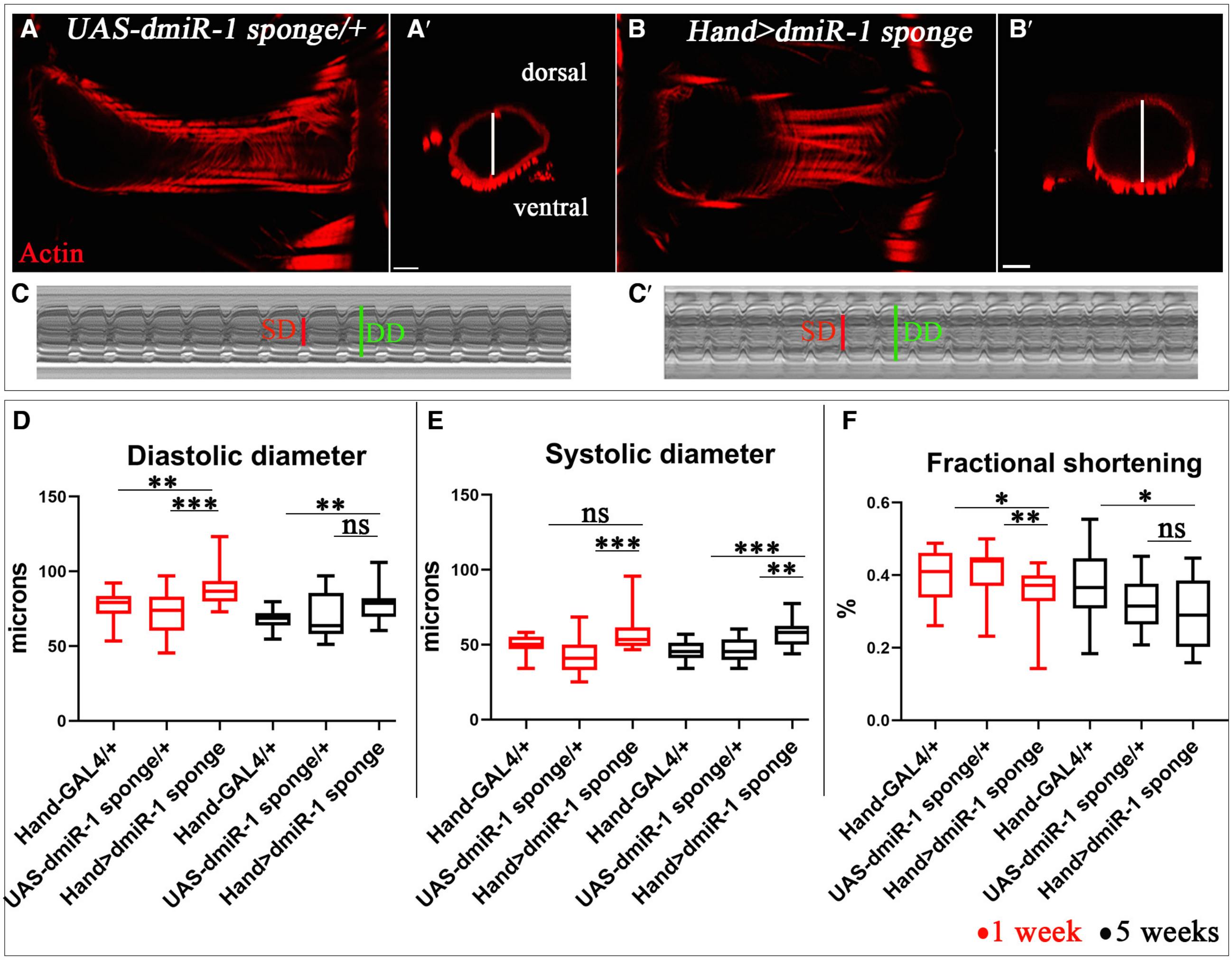

Figure 1. Heart-targeted *dmiR-1* attenuation causes DCM in *Drosophila*.

A–C′ (A) Control (*UAS-dmiR-1 sponge*) and (B) mutant (*Hand > dmiR-1 sponge*) adult hearts from 1-week-old flies labeled for F-actin (red). (A′, B′) Cross-sections of cardiac tubes 3D-reconstructed using Imaris software. White line shows the enlargement of lumen cardiac tube in *Hand > dmiR-1 sponge* (B′) in comparison with control (*UAS-dmiR-1 sponge*) (A′). M-mode records from 1-week-old control (*UAS- dmiR-1 sponge*) (C) and *Hand > dmiR-1 sponge* (C′) flies showing increased diastolic (green) and systolic diameters (red) in the *dmiR-1 sponge* context (C′).

D–F Box plots showing diastolic (D) and systolic (E) diameters and cardiac contractility analyses (percent fractional shortening) (F) performed using SOHA approach for controls (*Hand-Gal4* and *UAS-dmiR-1 sponge*) and *Hand > sponge dmiR-1* contexts at ages 1 and 5 weeks. $n = 20$ hearts. Central band corresponds to median. Whiskers correspond to Min to Max. Box corresponds to interquartile range from 25th to 75th percentile.

Data information: Scale bar = 20 μm. *P*-value < 0.05 is considered statistically significant. (*) *P*-value = 0.033, (**) *P*-value = 0.021, (***) *P*-value = 0.0002. The nonparametric Mann–Whitney test was performed to compare control samples and samples of interest.
Source data are available online for this figure.

dmiR-1 target Multiplexin is up-regulated in DCM-developing DM1 flies

To identify *dmiR-1* target genes involved in DM1-associated DCM, we first performed *in silico* screening by mapping *Drosophila*-specific *dmiR-1* seed sites annotated in miRBase (Griffiths-Jones *et al*, 2006; http://www.mirbase.org/) on 3′UTR regions of *Drosophila* genes up-regulated in the hearts of two DCM-developing DM1 contexts at 5 weeks of age (Table EV1; Fig 3A) (Auxerre-Plantié *et al*, 2019). We identified a set of 124 candidate genes that contain a potential *dmiR-1* seed site and among them *Multiplexin* (*Mp*) (Table EV1, scheme in Fig 3A).

Mp, a *Drosophila* ortholog of mammalian Collagen XVIII and Collagen XV, belongs to the family of multi-domain collagens. It is composed of an N-terminal thrombospondin-related domain, followed by multiple Collagen repeats, a Collagen trimerization domain, and a C-terminal endostatin domain (Meyer & Moussian, 2009). Mp also contains consensus glycosaminoglycan (GAG) attachment sites, and biochemical analysis of the protein extracted from embryonic tissues revealed the presence of chondroitin sulfate (CS) chains, making it more like human collagen XV than collagen XVIII in this respect. In the embryonic *Drosophila* heart, Mp is expressed in cardioblasts of the heart properly but not in aorta (Meyer & Moussian, 2009; Harpaz

© 2023 The Authors

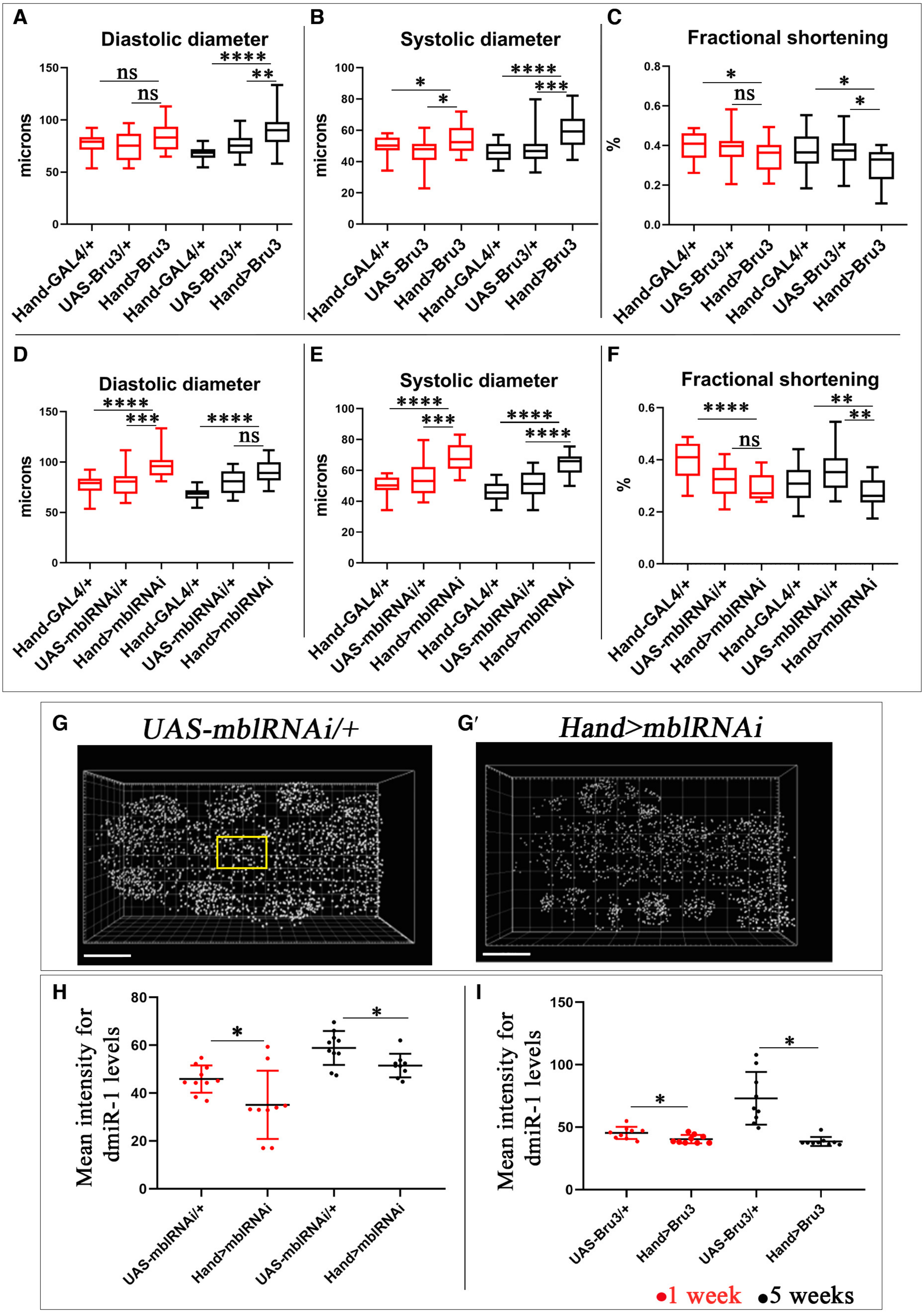
A
Diastolic diameter
B
Systolic diameter
C
Fractional shortening
D
Diastolic diameter
E
Systolic diameter
F
Fractional shortening
microns
%
Hand-GAL4/+
UAS-Bru3/+
Hand>Bru3
UAS-mblRNAi/+
Hand>mblRNAi
G
UAS-mblRNAi/+
G′
Hand>mblRNAi
H
I
Mean intensity for dmiR-1 levels
•1 week •5 weeks

Figure 2.

◀ **Figure 2. DM1 flies develop DCM phenotype with a reduced *dmiR-1* level in cardiac cells.**

A–F Box plots showing cardiac tube size (diastolic (A, D) and systolic (B, E) diameters) and contractility (percent fractional shortening (C, F)) analyses performed using SOHA approach for controls (*Hand-Gal4*, *UAS-Bru3*, and *UAS-mblRNAi*) and DM1 contexts (*Hand* > *Bru3* and *Hand* > *mblRNAi*), at ages 1 and 5 weeks. *n* = 20 hearts. Central band corresponds to median. Whiskers correspond to Min to Max. Box corresponds to interquartile range from 25th to 75th percentile.

G, G′ Representative spot views generated using Imaris from *in situ* hybridization with miRCURY LNA probe for *dmiR-1* and used for quantification of *dmiR-1* levels. Spot views of *dmiR-1* in hearts of 1-week-old control (*UAS-mblRNAi*) (G) and DM1 flies (*Hand* > *mblRNAi*) (G′) are shown. Each spot represents a pool of *dmiR-1* transcripts detected in the same area. The zoom region in G corresponds to area in which FISH signal was quantified.

H, I Scatter plot graph showing the signal intensity quantified in cardioblasts of 1- and 5-week-old flies for controls (*UAS-Bru3*, *UAS-mblRNAi*) and DM1 contexts (*Hand* > *Bru3*, *Hand* > *mblRNAi*). *n* = 9 hearts. Bars correspond to mean ± SD.

Data information: Scale bar = 40 μm. *P*-value < 0.05 considered statistically significant. (*) *P*-value = 0.033, (**) *P*-value = 0.021, (***) *P*-value = 0.0002, (****) *P*-value < 0.0001. The nonparametric Mann–Whitney test was performed to compare control samples and samples of interest.
Source data are available online for this figure.

et al, 2013). The Mp was shown to be deposited in a polarized way along the heart lumen during cardiac tube formation (Meyer & Moussian, 2009) and involved in lumen shaping by enhancing Slit/Robo activity (Harpaz *et al*, 2013). Mp overexpression in the developing embryonic heart leads to an enlargement of the heart lumen and is sufficient to promote an increase of the embryonic aorta diameter to that of the heart proper (Harpaz *et al*, 2013). We thus reasoned that Mp could be involved in DM1-associated DCM.

Here, we show that Mp is also expressed in the adult fly heart (Fig EV4A–C″). Consistent with the predicted location of an extracellular matrix protein, Mp was detected on the luminal and external surfaces of the cardiomyocytes and was also present on the underlying adult heart ventral longitudinal muscles (VLM) (Fig EV4A–C″).

The predicted *dmiR-1*-binding site within the *Mp*3′UTR region (Fig 3B) is expected to negatively regulate *Mp* transcript level in the presence of *dmiR-1*. To assess the biological relevance of this binding site *in vivo*, we cloned the *Mp3′UTR* fragment carrying the *dmiR-1* seed site downstream of the GFP coding sequence to generate the *UAS-GFP-Mp3′UTR* transgenic line. In parallel, the *UAS-GFP-ΔMp3′UTR* line was created in which the *dmiR-1* binding site was deleted from the *3′UTRMp* sequence. Both GFP sensor lines were then combined with the *UAS-dmiR-1* line to generate double transgenic lines *UAS-GFP-Mp3′UTR; UAS-dmiR-1* (Fig 3C, C′, D, and D′) and *UAS-GFP-ΔMp3′UTR*; *UAS-dmiR-1* (Fig 3E, E′, F, and F′), respectively. We found that the expression of *dmiR-1* in *Hand* > *GFP-3′UTRMp* hearts significantly reduced the GFP signal in cardiac and pericardial cells (Fig 3D′), suggesting that *dmiR-1* binds to the predicted seed site and negatively regulates *Mp* mRNA expression in the adult fly heart. The deletion of the *dmiR-1* binding site in *Hand* > *GFP-Δ3′UTRMp; dmiR-1* heart prevented the repressive effects of *dmiR-1* (Fig 3F′), demonstrating that the GFP silencing observed in *Hand* > *GFP-Mp3′UTR; dmiR-1* hearts (Fig 3D′) is *dmiR-1* dependent. This finding is also consistent with increased Mp protein levels detected in hearts of the *Hand* > *dmiR-1-sponge* context (Fig EV4J) mimicking *miR-1* attenuation and leading to DCM.

We then tested whether Mp protein level increased in DM1 contexts with DCM characterized by a reduced *dmiR-1*. We detected a significant increase in Mp protein level on the surface of cardiomyocytes in DCM-developing lines (*Hand* > *mblRNAi* and *Hand* > *Bru3*) in both young and aged flies (Fig 3G–L). A similar increase in Mp expression was found in DM1 pericardial cells (Fig EV4D–I).

Heart-targeted Mp overexpression leads to DCM

Mp protein level increases in DCM-developing DM1 hearts and in the heart-specific *dmiR-1* attenuation context causing DCM. Moreover, the 3′UTR region of *Mp* carries a *dmiR-1* binding site, making Mp an *in vivo dmiR-1* target in the heart. All these observations prompted us to determine whether the increased cardiac Mp level could lead to DCM. To generate cardiac Mp gain-of-function, we crossed *Hand-Gal4* with the *UAS-Mp 3HNC1* (*UAS-Mp*) line (Meyer & Moussian, 2009). The *Hand* > *Mp* adult flies expressed a high Mp protein level in the heart (Fig 4B and D) in comparison to the control (*UAS-Mp*) (Fig 4A and C). Already at age 1 week, the Mp gain-of-function flies displayed a larger heart tube diameter and a wider cardiac lumen than control (*UAS-Mp*) (Fig 4A′–D′). The diastolic and systolic diameters of hearts overexpressing Mp were also significantly increased at both 1- and 5-week-old *Hand* > *Mp* flies relative to controls (Fig 4E, F, H, and H′) with myofibrillar disarray associated with the enlarged heart tubes (Fig EV1D and D′). The cardiac dilation was concomitant with significantly reduced fractional shortening and thus adversely affected cardiac contractility (Fig 4G). These findings demonstrate that a heart-specific increase in Mp protein level is detrimental to cardiac function, leading to DCM in flies.

To further analyze the effects of Mp expression level on heart morphology and function, we tested the impact of cardiac-specific Mp knockdown at 1 and 5 weeks of age. The *Hand* > *Mp RNAi* adult flies expressed a lower Mp protein level in the heart (Fig 5B′ and C) compared with control (*UAS-Mp RNAi*) (Fig 5A′). We observed a reduced heart lumen in *Hand* > *Mp RNAi* flies (Fig 5B) compared with controls (Fig 5A) and a significant decrease in diastolic and systolic diameters at ages 1 and 5 weeks (Fig 5D and E) without effect on cardiac contractility (Fig 5F).

Taken together, our findings demonstrate that Mp expression level plays a critical role in setting the size of the cardiac lumen and the systolic and diastolic fly heart variables, and its overexpression in the heart leads to DCM. We thus infer that the reduced *dmiR-1* leading to the up-regulation of its direct target Mp promotes the development of DCM in the DM1 context (see scheme in Fig 4I).

Mp counterpart Col15A1 is up-regulated and *miR-1* is down-regulated in cardiac samples of DM1 patients with DCM

Given the increased Mp level in DM1 fly models developing DCM, we examined whether the expression of its human ortholog Col15A1 was also up-regulated in cardiac cells of DM1 patients. Of three DM1 cardiac tissue samples tested, two were from DM1 patients with DCM (Fig 6A and B). We observed that DM1 cardiac cells showed an increase in Col15A1 expression compared with cardiac cells from healthy donors that are nonsignificant at RNA levels

(Fig 6A) but significant at protein levels (Fig 6B). We also noticed differentially higher Col15A1 levels in DM1 patients with DCM compared with cardiac cells from DM1 patients without DCM (Fig 6A and B).

Since the up-regulation of Mp was associated with *dmiR-1* loss-of-function in DM1 flies, we tested the expression of *miR-1* in heart samples from DM1 patients. We observed that DM1 patients showed significant down-regulation of *miR-1* in cardiac tissue in comparison with controls (Fig 6C). Thus, like in *Drosophila* DM1 models, the down-regulation of *miR-1* and the concomitant up-regulation of Col15A1 in the heart correlate with DCM in DM1 patients.

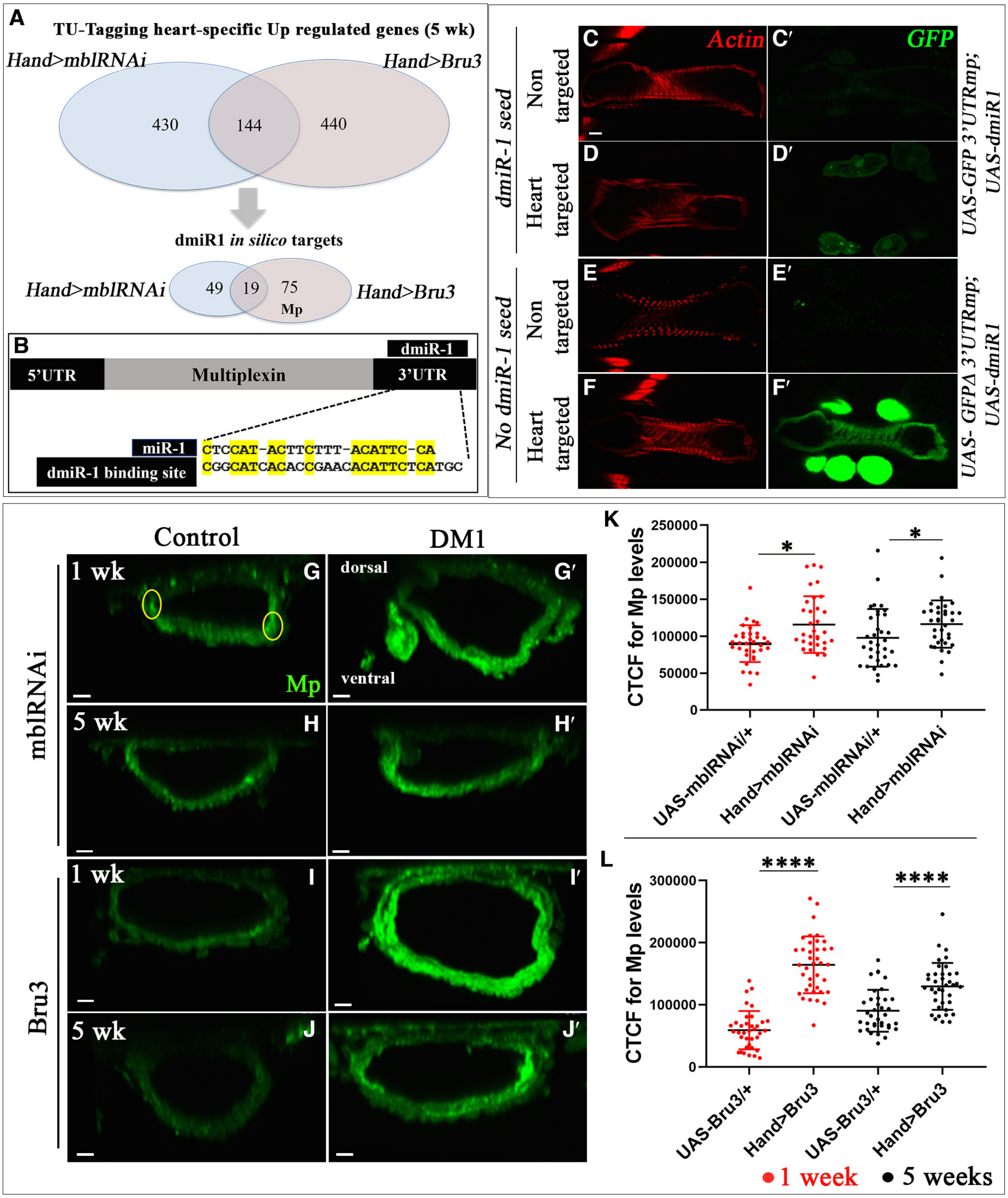

Figure 3.

© 2023 The Authors

Figure 3. Multiplexin, a new cardiac *dmiR-1* target, is up-regulated in DCM-developing DM1 flies.

A Up-regulated genes identified by heart-specific transcriptomic approach (TU-tagging) (Auxerre-Plantié *et al*, 2019) in DM1 contexts developing DCM (*Hand* > *mblRNAi* and *Hand* > *Bru3*) aged of 5 weeks (top Venn diagrams). Lower Venn diagrams show DM1 up-regulated genes identified *in silico* as potential *dmiR-1* targets.

B Alignment of *dmiR-1* binding site with 3′UTR region of *Multiplexin*.

C–F′ *dmiR-1* binding site in *3′UTRMp* region is required to negatively regulate Mp expression *in vivo*. Adult hearts from transgenic GFP sensor lines carrying *3′UTRMp* region with (*UAS-GFP-3′UTRMp*) or without *dmiR-1* seed site (*UAS-GFP Δ3′UTRMp*). These lines were combined with the *UAS-dmiR-1* line to generate double transgenic lines and tested for GFP expression in nontargeted context (crossed with w^{1118}) (C, E) or in heart-targeted context (crossed with heart-specific driver *Hand-Gal4*) (D, F). When crossed with *Hand-Gal4*, GFP expression in *Hand* > *GFP-3′UTRMp; dmiR-1* line (carrying *dmiR-1* seed site) (D′) is attenuated in cardiac tube and pericardial cells compared with *Hand* > *GFP Δ3′UTRMp; dmiR-1* line (lacking *dmiR-1* seed site) (F′). Scale bar = 40 μm.

G–J′ Cross-sections of adult hearts from 1- and 5-week-old controls (*UAS-mblRNAi*, *UAS-Bru3*) (G–J) and DM1 contexts (*Hand* > *mblRNAi*, *Hand* > *Bru3*) (G′–J′) labeled for Mp (green). Highlighted regions in G correspond to areas used for quantifications of the fluorescent signals. Scale bar = 10 μm.

K, L Graphs of Mp signal quantification in cardioblasts from adult flies aged 1 and 5 weeks for controls (*UAS-mblRNAi*, *UAS-Bru3*) and DM1 contexts (*Hand* > *mblRNAi*, *Hand* > *Bru3*) using the CTCF method. For each genotype, 9 hearts were analyzed and fluorescence intensity was measured in two regions from segment A3 and two regions from segment A4. Bars correspond to mean ± SD.

Data information: *P*-value < 0.05 considered statistically significant; (*) *P*-value = 0.033; (**) *P*-value = 0.021; (***) *P*-value = 0.0002; (****) *P*-value < 0.0001. The non-parametric Mann–Whitney test was performed to compare control samples and samples of interest.
Source data are available online for this figure.

Heart-specific attenuation of Mp rescues DM1-associated DCM phenotype

To determine whether the increased Mp/Col15A1 levels could contribute to the DCM phenotype in DM1, we applied our DM1 fly models and performed genetic rescue experiments by attenuating Mp expression in the DCM-developing *Hand* > *Bru3* context and we used *UAS-UPRT; Bru3* as a negative control (*UPRT* encodes uracil phospho-ribosyltransferase enzyme). Young 1-week-old *Hand* > *UPRT; Bru3* flies do not present DCM (Fig EV5A–C). However, 5-week-old flies from this line show a significant increase in diastolic and systolic diameter in comparison to the control (Fig 6D and E) with significant decrease in heart contractility measured by fractional shortening (Fig 6F). When Mp expression is attenuated via RNAi in *Hand* > *Bru3* DM1 context (*Hand* > *Bru3;Mp RNAi*), diastolic and systolic diameters are reduced in comparison to DM1 flies (*Hand* > *UPRT; Bru3*) (Fig 6D, E, G, H, and I), and the cardiac contractility defect is ameliorated and becomes similar to that of aged control flies (*UAS-UPRT; Bru3*) (Fig 6F). Thus, heart-specific attenuation of Mp rescues the DCM phenotype in aged DM1 flies.

Discussion

Myotonic dystrophy type 1 is the most common muscular dystrophy in adults. Cardiac repercussions including DCM are among the main causes of death in DM1 (Groh *et al*, 2008). However, the underlying mechanisms remain poorly understood, impeding the development of adapted treatments. As we previously demonstrated (Souidi *et al*, 2018; Auxerre-Plantié *et al*, 2019; Souidi & Jagla, 2021), *Drosophila* DM1 models recapitulate all the cardiac phenotypes observed in DM1 patients and so could help gain insight into gene deregulations underlying DM1-associated DCM.

DM1 fly models show reduced *dmiR-1* in cardiac cells and develop DCM

In humans, DCM is characterized by left ventricular dilation and systolic dysfunction defined by a depressed ejection fraction. Similarly, in DCM-developing flies, the cardiac tube is enlarged and shows an increased diastolic and systolic diameter with reduced contractility. The loss of cardiac miRNAs and in particular *miR-1* has already been correlated to DCM and heart failure in mice (Rao *et al*, 2009; Wei *et al*, 2014) and observed in patients with end-stage DCM (Ikeda *et al*, 2007). *miR-1* sequence is highly conserved between *Drosophila* and Human (Fig EV2F), and it is well known that it regulates genes involved in cardiac development and function including *Nkx2.5*, *SRF*, and components of *WNT* and *FGF* signaling pathways (Kura *et al*, 2020) and that its level is reduced in the pathological context of DM1 (Rau *et al*, 2011). However, it was not known whether the low *miR-1* level caused DM1-associated DCM, nor what were the downstream *miR-1* targets. Here, we show that two heart-targeting *Drosophila* DM1 models, *Hand* > *mblRNAi* and *Hand* > *Bru3* mimicking sequestration of MBNL1 and stabilization of CELF1, respectively, developed DCM and showed a reduced expression of *dmiR-1* in cardiac cells including cardiomyocytes and pericardial cells. Regarding the influence of *Hand-Gal4* driven expression in pericardial cells on the DM1 heart phenotypes, we previously tested all our DM1 models using cardioblast-specific *Tin-GAL4* driver. DM1 cardiac phenotypes such as conduction defects observed in the *Hand* > *Bru3* model (Auxerre-Plantié *et al*, 2019) and DCM observed in *Hand* > *mblRNAi* and *Hand* > *Bru3* models (Auxerre-Plantié *et al*, 2019 and this work) are observed when using *Tin-Gal4* driver. These results suggest that the cardiac phenotypes observed in the DM1 *Drosophila* heart, including DCM, are mainly due to gene deregulations within the cardiomyocytes. Because the overexpression of CELF1 (Koshelev *et al*, 2010) and the loss of MBNL1 (Lee *et al*, 2013) also result in DCM in mice, *Drosophila* appears well-suited to assessing the impact of reduced *miR-1* in DM1-associated DCM. One mechanism explaining why *miR-1* levels fall in the DM1 context is the sequestration of MBNL1, which can no longer play its physiological role in *pre-miR-1* processing into mature *miR-1* (Rau *et al*, 2011). Here, we observed reduced *dmiR-1* also upon the cardiac overexpression of CELF1 ortholog Bru3. How CELF1/Bru3 impinges on *miR-1* levels is not fully understood, but it was demonstrated that CELF1 could bind UG-rich miRNAs (such as *miR-1*) and mediate their de-adenylation and degradation by recruiting poly(A)-specific ribonuclease (PARN) (Katoh *et al*, 2015). Given that *Drosophila* DM1 models developing DCM showed markedly reduced *dmiR-1* in cardiac cells, we sought to determine

© 2023 The Authors

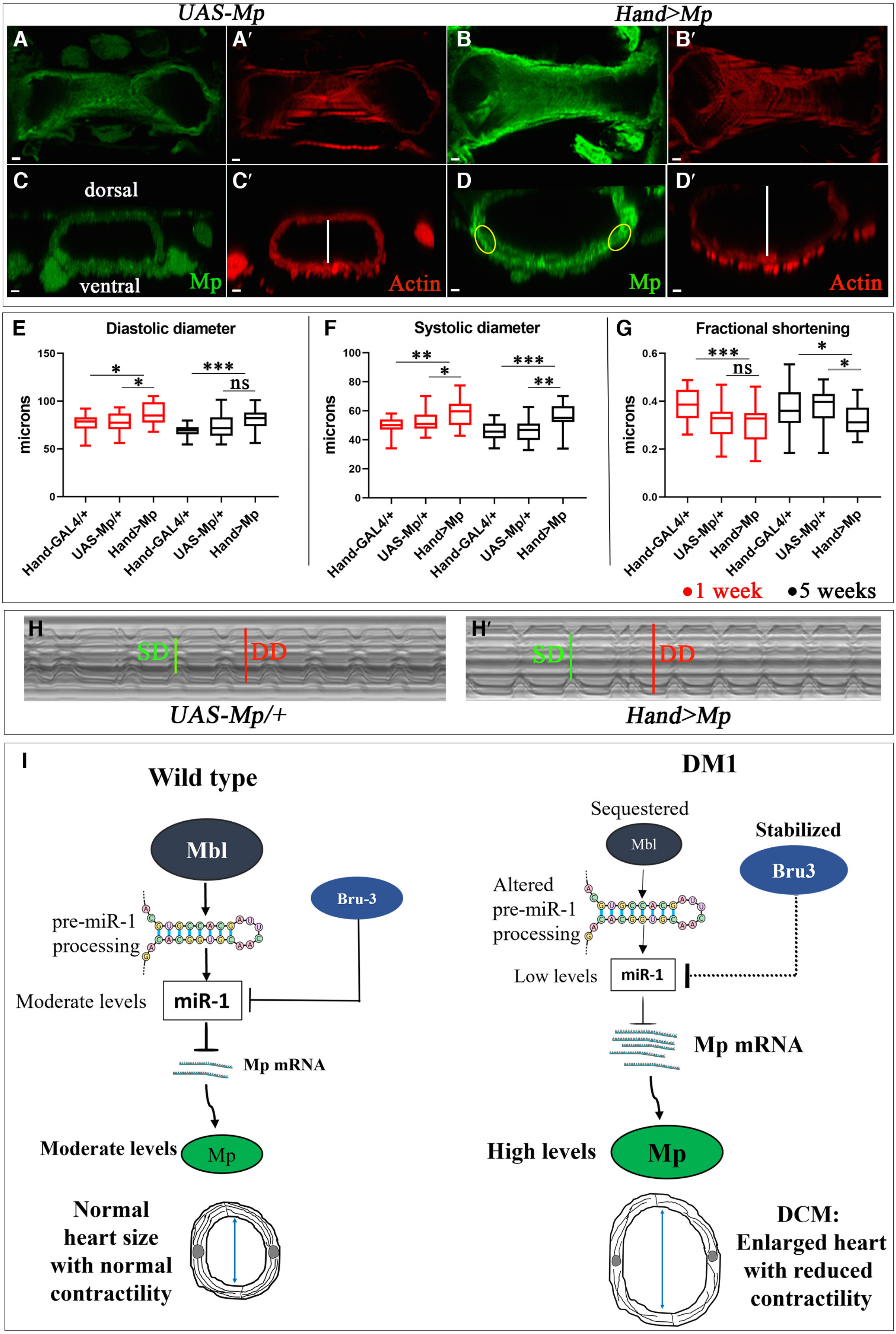

Figure 4.

 © 2023 The Authors

Figure 4. Heart-targeted Mp overexpression leads to DCM.

A–D′ Adult hearts from 1-week-old flies (A, A′, B, B′) and their cross-sections (C, C′, D, D′) labeled for Mp (green) and actin (red) for controls (*UAS-Mp*) and Mp overexpression context (*Hand > Mp*). Highlighted regions in D correspond to areas used for quantifications of the fluorescent signals. Scale bar = 5 μm.

E–G Box plots showing cardiac variables (diastolic (E) and systolic (F) diameters) and percent fractional shortening (G) for controls (*Hand-Gal4* and *UAS-Mp*) and Mp overexpression (*Hand > Mp*) conditions at 1 and 5 weeks. Notice an increase in diastolic and systolic diameters with reduced cardiac contractility in *Hand > Mp* in comparison with controls. $n = 20$ hearts. Central band corresponds to median. Whiskers correspond to Min to Max. Box corresponds to interquartile range from 25th to 75th percentile.

H, H′ M-modes representing cardiac variables in 5-week-old control (*UAS-Mp*) (H) and Mp-overexpressing (*Hand > Mp*) (H′) flies.

I Scheme presenting cardiac role of *dmiR-1* and its target *Mp* in DCM-developing *Drosophila* DM1 models. In wild-type, *Drosophila* heart mbl promotes *pre-dmiR-1* processing. Bru3 has a potential antagonistic role in the destabilization of *dmiR-1*. As a result, in this context *dmiR-1* and its target Mp levels are moderate. In DM1, mbl is sequestered and Bru3 is stabilized, causing inefficient processing of *pre-dmiR-1* and destabilization of mature *dmiR-1*. As a result, in the DM1 context, the *dmiR-1* level is reduced while its target Mp level is high. This leads to an enlarged heart with adversely affected contractility and myofibrillar disarray in the heart tubes.

Data information: *P*-value < 0.05 considered statistically significant. (*) *P*-value = 0.033, (**) *P*-value = 0.021, (***) *P*-value = 0.0002. The nonparametric Mann–Whitney test was performed to compare control samples and samples of interest.
Source data are available online for this figure.

whether heart-targeted attenuation of *dmiR-1* was sufficient to induce DCM: *dmiR-1* knockdown in the heart mimics DM1-associated DCM.

Col 15A1 ortholog Mp is a new *miR-1* target involved in DM1-associated DCM

To identify candidate *dmiR-1* target genes involved in DCM we performed *in silico* screening for *dmiR-1* seed sites in the 3′UTR regions of genes up-regulated in cardiac cells at 5 weeks of age (Auxerre-Plantié *et al*, 2019) in DM1 models developing DCM (see Table EV1). Among 1,189 3′UTR sequences tested, we found that 162 bore potential *dmiR-1* seed sites, including the 3′UTR of *Multiplexin* (*Mp*). *Mp* codes for extracellular matrix protein belonging to a conserved collagen XV/XVIII family. We top-ranked *Mp* because of its known role in setting the size of the cardiac lumen (Harpaz *et al*, 2013). The embryos overexpressing Mp display an enlarged cardiac tube and conversely, $Mp^{-/-}$ embryos were found to present a narrower lumen with reduced contractility of the heart tube (Harpaz *et al*, 2013). In parallel, the mouse mutants of *Mp* ortholog, *Col15A1*, showed age-related muscular and cardiac deterioration linked to a degraded organization of the collagen matrix (Eklund *et al*, 2001; Rasi *et al*, 2010). This prompted us to examine how Mp was expressed in the adult fly heart and what the effect of its overexpression was. Using Mp specific antibody we detected Mp on the surface of the cardiac cells and found that it accumulated to a high level in both *Hand > mblRNAi* and *Hand > Bru3* DM1 lines. We also tested whether the *in silico* identified *dmiR-1* seed site was required for the regulation of Mp expression and confirmed that *Mp* is a direct *in vivo* target of *dmiR-1* in cardiac cells. As the potential binding site for human *miR-1* is present also in 3′UTR of *Col15A1* transcript (Fig EV2G) we hypothesize that *Mp/Col15A1* are evolutionarily conserved *miR-1* targets. Consistent with its role downstream of *dmiR-1*, Mp overexpression in the heart mimicked the *dmiR-1* knockdown phenotype, leading to a significantly enlarged heart with reduced contractility. Moreover, heart-specific attenuation of Mp expression in the *Hand > Bru3* DM1 context reduced heart dilation and rescued DCM phenotype in aged flies, thus demonstrating that increased Mp levels contribute to DCM observed in DM1 flies. Previous reports (Gil-Cayuela *et al*, 2016; Louzao-Martinez *et al*, 2016) revealed increased expression levels of different collagens associated with DCM in both animal models and patients. Here, we report evidence that Col15A1 is specifically up-regulated at both transcript and protein levels in cardiac samples from DM1 patients and in particular in those with DCM, with down-regulation of *miR-1*. Altogether, the observations that Col15A1 expression level is abnormally elevated in DCM-developing DM1 patients and that attenuation of its *Drosophila* ortholog Mp could ameliorate the DCM phenotype suggest that Col15A1 could be a novel therapeutic target in DM1.

DCM, a complex cardiac condition with a poor prognosis in DM1

A large number of genes have so far been implicated in DCM, attesting to the complex molecular origin of this cardiac condition. For example, in *Drosophila*, DCM was observed in mutants of genes encoding contractile and structural muscle proteins such as Troponin I (TpnI), Tropomyosin 2 (Tm2), δ-sarcoglycan and Dystrophin but also associated with deregulations of EGF, Notch, Cdc42 and CCR4-Not signaling pathway components (reviewed in Wolf, 2012). In humans, DCM-causing mutations were also identified in a large number of genes including those encoding cytoskeletal proteins such as FLNC, nuclear membrane protein LMNA or involved in sarcomere stability (Titin, TNNT2, MYH7, TPM1) but also RNA-binding protein RBM20 (McNally & Mestroni, 2017).

Here, we focused on DCM associated with DM1. A previous study on a mouse model overexpressing CELF1 and developing DCM (Wang *et al*, 2015), identified down-regulation of *Transcription factor A mitochondrial* (*Tfam*), *Apelin* (*Apln*), and *Long-chain fatty acid-CoA ligase 1* (*Acsl1*) as potentially associated with DCM. It was suggested that CELF1 might regulate their mRNA stability by binding to their 3′UTR regions and causing destabilization and degradation of their transcripts (Chang Kuei-Ting *et al*, 2017). In this DCM-developing mouse DM1 model, Col15a transcripts were elevated (Wang *et al*, 2015), but the role of Col15a in DCM was not analyzed. Here, using *Drosophila* DM1 models with a DCM phenotype, we identified up-regulation of Col15A1 ortholog Mp as a molecular determinant of DM1-associated DCM. We also link reduced *miR-1* levels in DCM-developing DM1 cardiac cells to the up-regulation of Mp, establishing that *Mp* is an *in vivo* target of *dmiR-1*.

Importantly, our findings show that in DM1 patients, Collagen 15A1 is up-regulated in the hearts of patients with DCM. In DM1 patients, the DCM phenotype appears several years after onset and

© 2023 The Authors

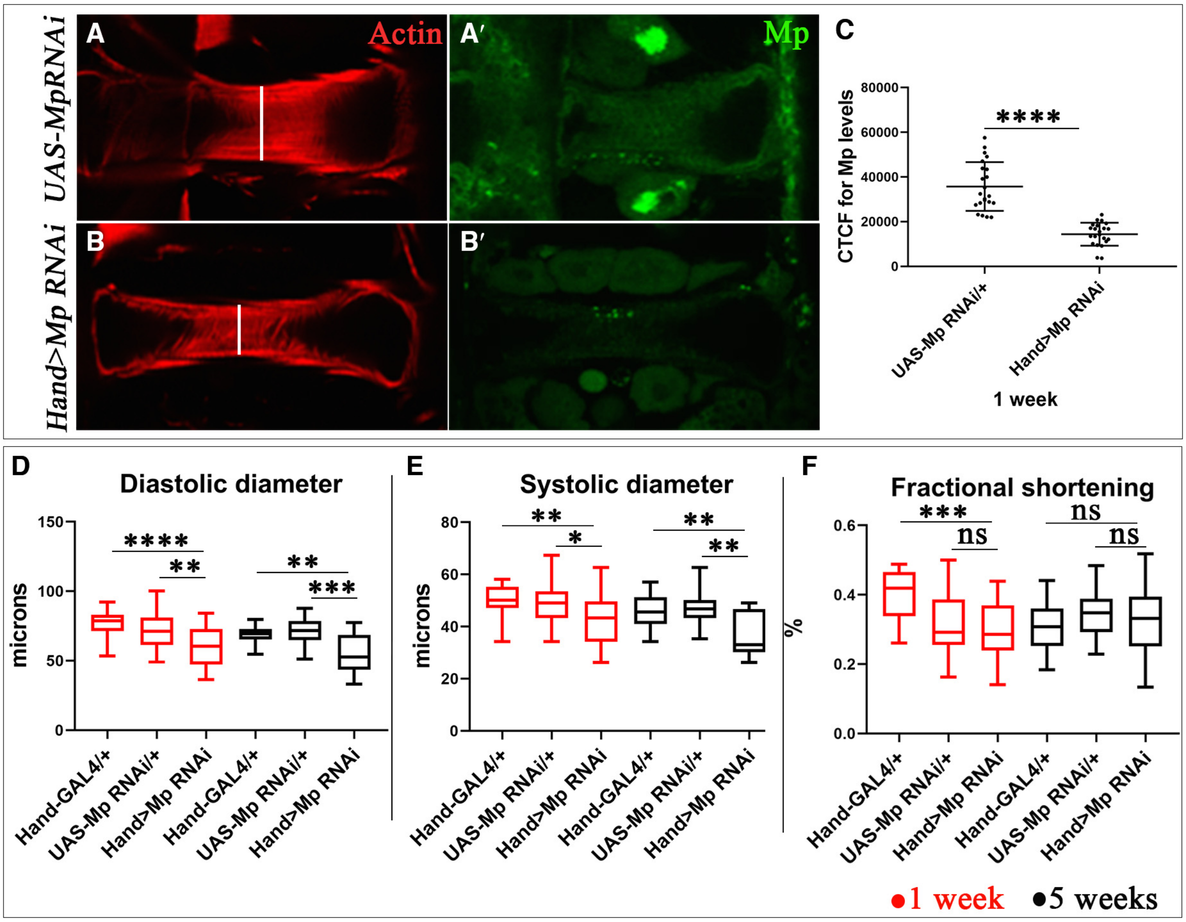

Figure 5. Mp loss-of-function leads to reduced heart size.

A–B′ Adult heart of 1 week of age labeled for Mp (green) and actin (red) for control (*UAS-Mp RNAi*) (A, A′) and mutant (*Hand* > *Mp RNAi*) contexts (B, B′).

C Fluorescence signal intensity quantification for Mp expression in cardioblasts in adult heart of 1 week for control (*UAS-Mp RNAi*) and mutant (*Hand* > *Mp RNAi*) contexts using the CTCF method. For each genotype, 9 hearts were analyzed and fluorescence intensity was measured in two regions from segment A3 and two regions from segment A4. Bars correspond to mean ± SD.

D–F Box plots showing cardiac size analyses (diastolic (D) and systolic diameters (E)) and percent fractional shortening (F) performed by SOHA approach for controls (*Hand-Gal4* and *UAS-Mp RNAi*) and mutant (*Hand* > *Mp RNAi*) contexts at 1 and 5 weeks of age, showing the reduced size of cardiac tube in *Hand* > *Mp RNAi* context in comparison to the *Hand-Gal4* control. $n = 20$ hearts. Central band corresponds to median. Whiskers correspond to Min to Max. Box corresponds to interquartile range from 25th to 75th percentile.

Data information: Scale bar = 20 μm. *P*-value < 0.05 considered statistically significant. (*) *P*-value = 0.033, (**) *P*-value = 0.021, (***) *P*-value = 0.0002, (****) *P*-value < 0.0001. The nonparametric Mann–Whitney test was performed to compare control samples and samples of interest.
Source data are available online for this figure.

is less common than the conduction system defects and arrhythmias (Nguyen *et al*, 1988; Lin *et al*, 1989). However, it is frequently associated with poor prognosis and indication for heart transplant (Papa *et al*, 2018).

In summary, we report evidence for the importance of *miR-1*-dependent gene deregulations in DM1 and identify *Mp* as a new *miR-1* target involved specifically in DM1-associated DCM. We also demonstrate that Mbl depletion and Bru3 up-regulation in the heart have overlapping impacts on DM1 pathogenesis, both leading to reduced *miR-1*, up-regulation of Mp, and so to DCM (see scheme in Fig 4I).

Our conclusion is that in a physiological context, Mp level is moderately triggered by Mbl-dependent regulation of *dmiR-1* processing and Bru3-dependent regulation of *dmiR-1* stability. However, in the DM1 context, Mbl is sequestered in nuclear foci while Bru3 levels increase, leading to a reduced *dmiR-1* and the up-regulation of its target gene *Mp*. Considering the known role of Mp as a positive regulator of cardiac lumen size (Harpaz *et al*, 2013) we would expect Mp accumulation in the adult heart also to promote heart tube enlargement, leading to the DCM phenotype. Whether like in embryos this Mp function involves the Slit/Robo signaling pathway remains to be investigated, but the finding that Robo2 is among identified *miR-1* targets up-regulated in DCM-developing DM1 flies (Table EV1) supports this possibility. Finally, the fact that Mp ortholog Col15A1 is highly elevated in cardiac samples from DM1 patients with DCM and that reducing Mp rescues the DCM

 © 2023 The Authors

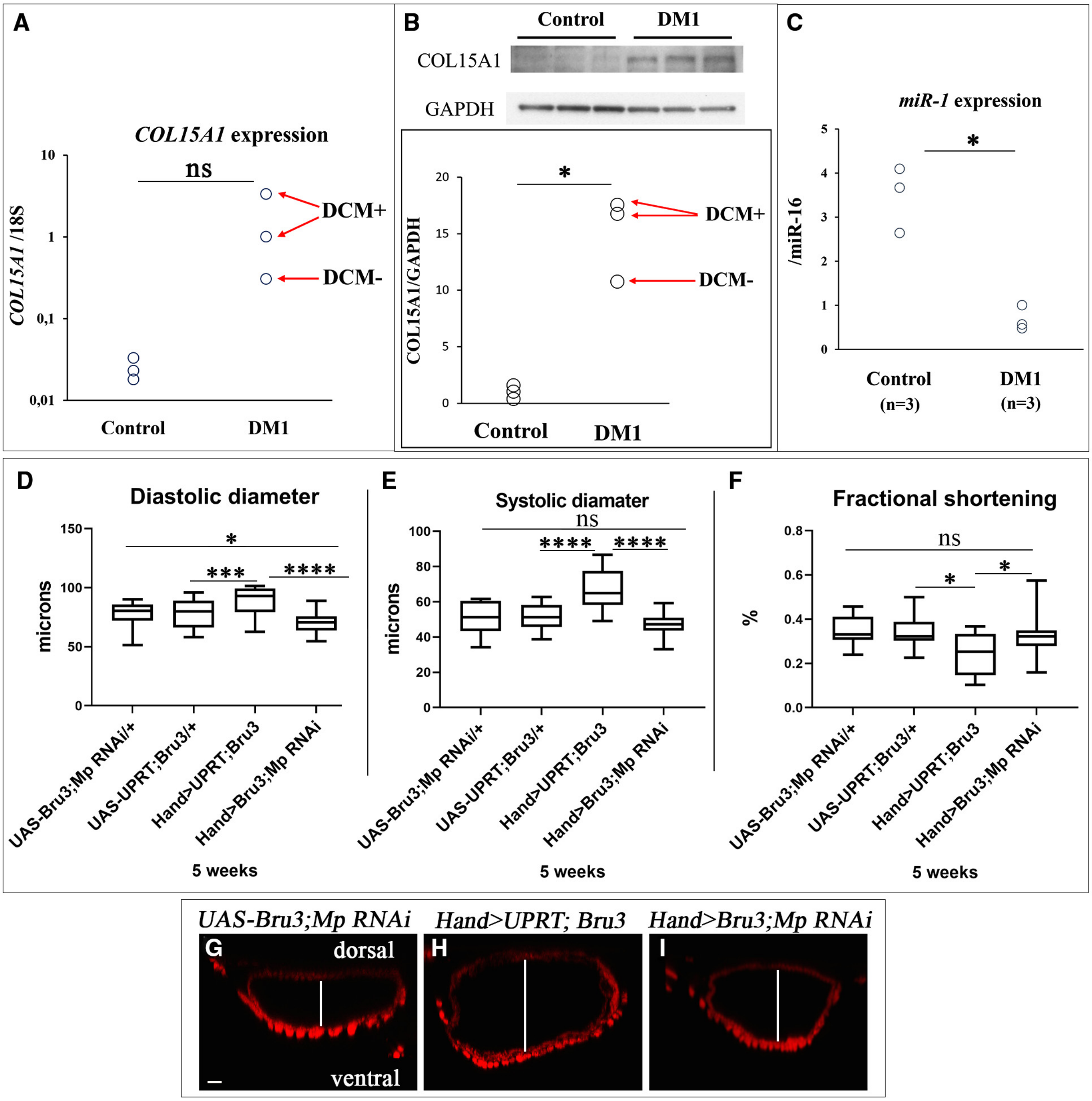

Figure 6. Increased cardiac expression of human Mp ortholog Col15A1 and low expression of *miR-1* are associated with DCM in DM1 patients.

A, B (A) *Col15A1* transcript levels tested by RT–qPCR and (B) Col15A1 protein levels tested by Western blot in cardiac samples from healthy donors and from DM1 patients with (DCM+) and without DCM (DCM−).

C *miR-1* levels tested by RT–qPCR in cardiac samples from healthy donors and from DM1 patients.

D, F Box plots showing cardiac variables (diastolic (D) and systolic (E) diameters and percent fractional shortening (F)) assessed by SOHA approach for controls (*UAS-Bru3; Mp RNAi and UAS-UPRT; Bru3*), Mp rescue (*Hand > Bru3; Mp RANi*) and DM1 context (*Hand > UPRT; Bru3*) at 5 weeks of age showing rescue of diastolic and systolic diameters in *Hand > Bru3; Mp RNAi* in comparison with controls (D, E) and rescue of cardiac contractility (F) $n = 20$ hearts. Central band corresponds to median. Whiskers correspond to Min to Max. Box corresponds to interquartile range from 25th to 75th percentile.

G–I Cross-sections of cardiac tubes 3D-reconstructed using Imaris software from 5-week-old control (*UAS-Bru3; MpRNAi*) (G), DM1 (*Hand > UPRT; Bru3*) (H) and Mp rescue (*Hand > Bru3; Mp RNAi*) (I) flies labeled for actin. The white line shows the diameter of the heart tube lumen. Scale bar = 20 µm.

Data information: *P*-value < 0.05 considered statistically significant. (*) *P*-value = 0.033, (***) *P*-value = 0.0002, (****) *P*-value < 0.0001. A parametric *t*-test was performed to compare control samples and samples of interest in (A, B, and C). The nonparametric Mann–Whitney test was performed to compare control samples and samples of interest in (D, E, F).

Source data are available online for this figure.

phenotype in DM1 fly model suggests that Mp/Col15A1 could be an attractive diagnostic and/or therapeutic target for DM1-associated DCM.

Materials and Methods

Drosophila stocks

All *D. melanogaster* stocks were grown and crossed at 25° C on standard fly food. In this study, we used three *Drosophila* DM1 models: *UAS-960CTG* (Picchio *et al*, 2013), *UAS-mblRNAi* (28732, VDRC Vienna, Austria), and *UAS-Bru3* (Picchio *et al*, 2013). For *dmiR-1* loss- and gain-of-function, we used *UAS-dmiR-1 sponge* (Fulga *et al*, 2015), *dmiR-1 KO* (58879, Bloomington, USA), and *UAS-dmiR-1* (41125, Bloomington, USA), respectively. For functional analyses of Mp, we used *UAS-MpRNAi-TRIP* (52981, Bloomington, USA) and *UAS-3HNC1* (Meyer & Moussian, 2009). For testing the rescue of dilated cardiomyopathy observed in *Hand > Bru3* line, we have recombined *UAS-Bru3* with *UAS-MpTRIP* line to generate *UAS-MpTRIP; Bru3* line. The *UAS-Bru3* line was also recombined with the *UAS-UPRT* (UAS-HA-UPRT Bloomington 27604) line to generate *UAS-UPRT; Bru3* was a negative control for this rescue experiment.

All inducible lines were crossed with the driver line *Hand-Gal4* (provided by Laurent Perrin, TAGC, Marseille, France) to induce the expression of transgenes specifically in the fly heart (cardioblasts and pericardial cells, with a low expression in VLM). Control lines were generated by crossing the above-cited UAS lines with w^{1118} line. *Hand-Gal4/+* control was generated also by crossing *Hand-Gal4* driver line with w^{1118}. w^{1118} is a mutant strain with a recessive white-eye phenotype.

Heartbeat analyses of adult *Drosophila* hearts

Physiological analyses of adult *Drosophila* hearts were performed on 1- and 5-week-old female flies using the Semi-automatic Optical Heartbeat Analysis (SOHA) approach protocol of Ocorr *et al* (2009). For each genotype, about 20 flies were analyzed. First, we proceeded to dissection and preparation of semi-dissected hearts: adult *Drosophila* flies were anesthetized with Fly Nap for 5 min, then placed, dorsal side down, into a petri dish coated with a thin layer of petroleum jelly. The head, the ventral thorax, and the ventral abdominal cuticle were removed in order to expose the abdominal contents. The ovaries and other organs as well as fats were then removed in order to expose heart tube attached to the dorsal cuticle. Dissection was performed in an oxygenated, artificial hemolymph (AH) solution composed of 108 mM Na^+, 5 mM KCl, 2 mM $CaCl_2$, 8 mM $MgCl_2$, 1 mM NaH_2PO_4, 4 mM $NaHCO_3$, 10 mM sucrose, 5 mM trehalose, 5 mM HEPES (pH 7.1, all reagents from Sigma Chemicals). The solution was oxygenated by air-bubbling for 15 to 20 min. The beating hearts were filmed by a digital camera on 30 s movie with the speed of 150 frames/s (Digital camera C9300, Hamamatsu, McBain Instruments, Chatsworth, CA) using microscope Zeiss (Axiophot, Zeiss) using immersion objective 10×. The heartbeats were recorded in A3 and A4 segments. The SOHA program based on Matlab R2009b software has been used for film analysis: The cardiac tube membrane during maximum diastole (relaxation) and maximum systole (contraction) were defined manually. One pair of marks identified the diastolic diameter, and one pair identified the systolic diameter. From this vertical row of pixels, an M-mode was generated to analyze the contraction and relaxation intervals: diastolic (DD), and systolic (SD) diameters, heart period (HP), and systolic (SI) and diastolic (DI) intervals. The diastolic and systolic diameters were used to calculate the fractional shortening (FS) using the formula: (((Diastolic diameter − Systolic diameter)/Diastolic diameter) × 100) (Ocorr *et al*, 2009).

Immunofluorescence staining of *Drosophila* heart

The adult hearts from 1- and 5-week-old females were dissected as described previously, then fixed with formaldehyde 4%. Samples were incubated with primary rat anti-Mp antibodies (1/100) (Harpaz *et al*, 2013) or goat anti-GFP (1/500) (Abcam ab5450) overnight at 4°C followed by 3 washes with PBS-Tween 0.1% of 10 min each, then secondary antibodies anti-rat Alexa-CY3 (1/150) or with anti-goat Alexa-488 (1/150) (Jackson ImmunoResearch), respectively, for 2 h at room temperature, Rhodamine phalloidin (1/1,000) (Thermo Fischer Scientific) was used to reveal actin filaments. The preparations were mounted in the Vectashield with DAPI (Vector Laboratories, Inc. Burlingame, CA). Immunofluorescence-labeled preparations were analyzed using a confocal microscope Leica SP8.

Quantification of Multiplexin immunolabeling

Fluorescent-labeled heart tissues were all processed and stained under the same conditions. The level of fluorescent signal was quantified using ImageJ software by CTCF (Corrected Total Cell Fluorescence) approach. CTCF is calculated according to the formula: Measured by the software Integrated Optical Density − (Area of select × mean fluorescence of background readings). For each heart, CTCF was measured in two regions from segment A3 and two regions from segment A4. For each genotype, 9 hearts were analyzed for cardioblasts with 4 measurements in regions for each, and 3 pericardial cells were analyzed from each heart.

Fluorescence in situ hybridization (FISH) using LNA-enhanced probes

For the detection of *dmiR-1*, we used fluorescence in situ hybridization (FISH) using double-labeled (enhanced) miRCURY LNA probes. First, flies 1- and 5-week-old were dissected and fixed for 30 min with 4% paraformaldehyde; rinsed at PBS1X-Tween 0.1% three-time, 5 min each. Samples were dehydrated through a series of increasing ethanol concentrations, transferred sequentially to 25, 50, 75, and 100% ethanol baths for 10 min each. After removing ethanol tissues were rehydrated through a series of decreasing ethanol concentrations, transferred sequentially to 50%, 25% ethanol baths for 10 min each, and then postfixed for 30 min with 4% paraformaldehyde. Then incubated with a solution composed of 50% PBT and 50% hybridization buffer (5 ml Formamide, 0.5 ml SSC 20X, 100 μl ssDNA, 20 μl tRNA 50 ng/μl, 5 μl Heparin 100 ng/μl, 10 μl Tween) for 5 min. The samples were finally incubated with hybridization solution for 1 h 30 min at 50°C. The hybridization mix including specific DIG-labeled probes (1 nM for U6 snRNA-positive control ref 339459, 20 nM for negative control scramble ref 339459 and 40 nM for DME-miR-1-3p ID: 339111) was added in

 © 2023 The Authors

each tube and samples were incubated at 50°C overnight. To remove nonspecific RNA hybridization, samples were washed with ISH (5 ml Formamide, 0.5 ml SSC 20X, 100 µl ssDNA, 5 µl Heparin 100 ng/µl, 10 µl Tween) for 30 min each at 50°C then with PBT. Slides were blocked in western blockage reagent (3 ml blockage reagent + 12 ml PBT) for 30 min, incubated with sheep anti-DIG POD antibodies (11633716001, Roche) for 2 h, treated with the TSA Plus Cyanine 3 System 1% (PerkinElmer, USA) for 5 min, blocked in 20% NGS diluted in PBT for 30 min. Finally, samples were incubated with primary antibodies (anti-Actin 1/250) overnight at 4°C and then with secondary antibodies coupled with Alexa448 (1/150). The preparations were mounted in a Vectashield with a DAPI medium. Immunofluorescence labeling preparations have been then analyzed using a confocal microscope Leica SP8.

FISH quantification

Stained *Drosophila* hearts were imaged in 3D to allow the quantification of RNA transcripts in the total volume of cardiac and pericardial cells. The transcripts hybridized with miRCURY LNA-enhanced probes appear on the images as luminous fluorescent spots. Each spot represents several *miR-1* transcripts. The 3D images were analyzed using Imaris (version 9.3.1) that allows the detection, the visualization of spatial distribution, and the quantification of intensity for each spot detected. To detect the real RNA spots of interest we have adjusted the threshold of intensity detection according to scramble. To determine the lower threshold in pericardial cells, we have analyzed the spots detected in 27 pericardial cells in 9 heart preparations labeled by scramble. We adjusted the lower threshold above the mean intensity of scramble spots (30) and the upper threshold (255) by default. With this background subtracting parameter, Imaris software generated *dmiR-1* spots and calculated the mean intensity for each spot detected and the average of the mean intensities for all *dmiR-1* spots. Similar approach has been applied for *dmiR-1* FISH quantification in cardioblasts.

RNA extraction and RT–qPCR on adult fly heart samples

Total RNA was isolated from 20 adult hearts from 1- and 5-week-old female flies, using Direct-zol™RNA Microprep (ref: R2060) from Zymo Research following the manufacturer's instructions. RNA quality and quantity were, respectively, assessed using Agilent RNA Screen Tape Assay on 4200 TapeStation System (Agilent Technologies) and Qubit RNA HS assay kit on a Qubit 3.0 Fluorometer (Thermo Fischer Scientific). Then, 150 ng total RNA was reverse transcribed using SuperScript IV Reverse Transcriptase kit (Invitrogen) with random primer mix, in a 20 µl reaction. Quantitative PCR was performed in 4 replicates in the final volume of 10 µl using Light SYBR Green PCR Master Mix (Roche, Applied Science) on a LightCycler 480 Real-Time PCR System (Roche, Applied Science). A 2 µl (3 ng) of cDNA were added to an SYBR Green Master Mix. We used Rp49 as a reference gene. The following pairs of primers were used: *Rp49*: forward GCTTCAAGGGACAGTATCTG and reverse AAACGCGGTTCTGCATGAG; *pre-dmiR-1*: forward TTCAGCCTTTG AGAGTTCCATG and reverse CGCCAGATTTCGCTCCATAC. The relative quantifications of transcripts were obtained with the ΔΔCt method. Finally, nonparametric Mann–Whitney tests were performed to compare control samples and samples of interest.

In silico search for *dmiR-1* target genes up-regulated in DM1 models

We aligned *dmiR-1-3p* sequence (*UGGAAUGUAAAGAAGUAUGGAG*; www.mirbase.org) on 3′UTR part of every *D. melanogaster* gene (dm6 *D. melanogaster* genome sequence) allowing 1 mismatch or indel using bowtie2 (Langmead & Salzberg, 2012). From this pool of potential *dmiR-1* targets, we selected those which are up-regulated (Fold change>1.7) in *Hand > Bru3* and/or *Hand > mblRNAi* DM1 contexts in comparison with the control *Hand > LacZ* at 5 weeks of age (transcriptomic data of our laboratory; Auxerre-Plantié *et al*, 2019).

Generating transgenic fly lines

To validate *Mp* as a direct target of *dmiR-1 in vivo*, we have generated double transgenic fly lines. For the generation of *UAS-GFP-3′UTRmp* line, approximately 470 base pairs surrounding the predicted *dmiR-1*-target site in 3′UTR of *Mp* were amplified directly from the genomic DNA from the w^{1118} flies using the primers Mp-F1: ATAACTAGTTGAGCGGAAACGGAAGGAAGAAGAGGAG and Mp-R1: ATATCTAGATGTTGTGAATGATGACGTTAGG and a high-fidelity DNA Polymerase enzyme (Thermo Scientific Phusion High-Fidelity DNA Polymerase) kit. For the *UAS-GFP-Δ3′UTRmp* line, the same steps were proceeded with primers Mp-F2: ATAACTAGTTGA TAAAACAAAACAAATCACAGCAC and Mp-R1: ATATCTAGA TGTTGTGAATGATGACGTTAGG to amplify about 350 bp without predicted *dmiR-1*-target site. The *Spe*I and *Xba*I restriction sites were incorporated into primers and introduced by PCR. PCR products were purified using NucleoSpin Plasmid clean-up kit after validation of inserts by electrophoresis on 1% agarose gel. After digestion of purified *3′UTRMp* fragments and *pUASP-PL-Venus* vector by *Spe*I and *Xba*I enzymes, we performed ligation between *3′UTRMp* fragments and the vector using the T4 DNA ligase kit (Invitrogen) according to the manufacturer's instructions. Purified vectors were then microinjected by the Fly Facility platform to generated transgenic lines. Finally, *UAS-GFP-3′UTRMp* and *UAS-GFP-Δ 3′UTRMp* transgenic lines were combined with *UAS-miR-1* (41125, Bloomington, USA) to generate *UAS-GFP-3′UTRMp; UAS-miR-1* and *UAS-GFP-Δ3′UTRMp; UAS-miR-1*, respectively, and then crossed with the driver line *Hand-GAL4*.

RNA extraction, RT–qPCR, and immunoblot on DM1 human hearts

Human ventricular cardiac muscle tissues were obtained at autopsy from 3 DM1 patients and 3 normal controls following informed consent. All experimental protocols were approved by the Institutional Review Board at Osaka University and carried out in accordance with the approved guidelines. Total mRNA was extracted and first-strand complementary DNA synthesized as described previously (Nakamori *et al*, 2008). RT–qPCR was performed using TaqMan Gene Expression assays (Hs00266332_m1 and 4333760F, Applied Biosystems) on an ABI PRISM 7900HT Sequence Detection System (Applied Biosystems), as described previously (Nakamori *et al*, 2011). The level of *COL15A1* mRNA was normalized to *18 S* rRNA. For *miR-1* expression analysis, micro-RNA was extracted from human cardiac muscle tissues by using miRNeasy Micro Kit (Qiagen). Follow-up qPCRs were

© 2023 The Authors

performed using TaqMan Advanced miRNA cDNA Synthesis Kit (Applied Biosystems) and TaqMan Advanced miRNA Assays (477820_mir) normalized by *miR-16-5p* (477860_mir). For protein analysis, cardiac muscle tissues were homogenized in a 10× volume of radioimmunoprecipitation assay buffer (25 mM Tris–HCl; pH 7.5; 150 mM NaCl; 1% NP-40; 1% sodium deoxycholate; and 0.1% sodium dodecyl sulfate) containing a protein inhibitor cocktail (Sigma-Aldrich). The homogenate was centrifuged for 10 min at 10,000 *g* and the supernatant was collected. Equal amounts of protein (40 µg) were separated by sodium dodecyl sulfate–polyacrylamide gel electrophoresis and transferred onto Immobilon-P membranes (Millipore), as previously described (Nakamori *et al*, 2008). Blots were blocked for nonspecific protein binding with 5% (w/v) nonfat milk and then incubated with a 1:500-diluted antibody against COL15A1 (Thermo Fisher Scientific) or 1:3,000-diluted antibody against GAPDH (glyceraldehyde 3-phosphate dehydrogenase) (Sigma-Aldrich). After repeated washings, the membranes were incubated with horseradish peroxidase-conjugated goat anti-rabbit IgG (Thermo Fisher Scientific). The ECL Prime Western Blotting Detection Reagent (Cytiva) and ChemiDoc Touch Imaging System (Bio-Rad) were used to detect the proteins.

Statistics

Nonparametric Mann–Whitney tests were performed to compare control samples and samples of interest in *Drosophila* model, and the *t*-test was performed to compare controls to DM1 context from heart samples of DM1 patients. All statistical analyses were performed using GraphPad Prism (version 8.0.1) software. Results are reported with P-value < 0.05 considered statistically significant.

Data availability

No primary datasets have been generated and deposited. Previously generated sequencing data have been deposited with the GEO-NCBI tracking system under accession number GSE109370 (http://www.ncbi.nlm.nih.gov/geo/query/acc.cgi?acc=GSE109370) and used here to identify potential dmiR-1 targets listed in Table EV1.

Expanded View for this article is available online.

Acknowledgements
We would like to thank *Drosophila* Bloomington and VDRC stock centers. This work was funded by the AFM-Telethon strategic grant to MyoNeurAlp consortium (to K.J.) and Intramural Research Grant (2–5) for Neurological and Psychiatric Disorders of National Center of Neurology and Psychiatry (to M.N.).

Author contributions
Anissa Souidi: Formal analysis; investigation; visualization; methodology; writing – original draft. **Masayuki Nakamori:** Formal analysis; investigation; methodology. **Monika Zmojdzian:** Formal analysis; investigation; methodology. **Teresa Jagla:** Investigation. **Yoan RENAUD:** Formal analysis. **Krzysztof Jagla:** Conceptualization; data curation; supervision; funding acquisition; writing – review and editing.

Disclosure and competing interest statement
The authors declare that they have no conflict of interest.

References

Auxerre-Plantié E, Nakamori M, Renaud Y, Huguet A, Choquet C, Dondi C, Miquerol L, Takahashi MP, Gourdon G, Junion G *et al* (2019) Straightjacket/α2δ3 deregulation is associated with cardiac conduction defects in myotonic dystrophy type 1. *Elife* 8: e51114

Dasgupta T, Ladd AN (2012) The importance of CELF control: molecular and biological roles of the CUG-BP, Elav-like family of RNA binding proteins. *Wiley Interdiscip Rev RNA* 3: 104–121

Eklund L, Piuhola J, Komulainen J, Sormunen R, Ongvarrasopone C, Fässler R, Muona A, Ilves M, Ruskoaho H, Takala TES *et al* (2001) Lack of type XV collagen causes a skeletal myopathy and cardiovascular defects in mice. *Proc Natl Acad Sci USA* 98: 1194–1199

Fardaei M, Larkin K, Brook JD, Hamshere MG (2001) In vivo co-localisation of MBNL protein with DMPK expanded-repeat transcripts. *Nucleic Acids Res* 29: 2766–2771

Fardaei M, Rogers MT, Thorpe HM, Larkin K, Hamshere MG, Harper PS, Brook JD (2002) Three proteins, MBNL, MBLL and MBXL, co-localize in vivo with nuclear foci of expanded-repeat transcripts in DM1 and DM2 cells. *Hum Mol Genet* 11: 805–814

Fernandez-Costa JM, Garcia-Lopez A, Zuñiga S, Fernandez-Pedrosa V, Felipo-Benavent A, Mata M, Jaka O, Aiastui A, Hernandez-Torres F, Aguado B *et al* (2013) Expanded CTG repeats trigger miRNA alterations in *Drosophila* that are conserved in myotonic dystrophy type 1 patients. *Hum Mol Genet* 22: 704–716

Fulga TA, McNeill EM, Binari R, Yelick J, Blanche A, Booker M, Steinkraus BR, Schnall-Levin M, Zhao Y, DeLuca T *et al* (2015) A transgenic resource for conditional competitive inhibition of conserved *Drosophila* microRNAs. *Nat Commun* 6: 7279

Garcia-Lopez A, Monferrer L, Garcia-Alcover I, Vicente-Crespo M, Alvarez-Abril MC, Artero RD (2008) Genetic and chemical modifiers of a CUG toxicity model in *Drosophila*. *PLoS One* 3: e1595

Gil-Cayuela C, Roselló-LLetí E, Ortega A, Tarazón E, Triviño JC, Martínez-Dolz L, González-Juanatey JR, Lago F, Portolés M, Rivera M (2016) New altered non-fibrillar collagens in human dilated cardiomyopathy: role in the remodeling process. *PLoS One* 11: e0168130

Griffiths-Jones S, Grocock RJ, van Dongen S, Bateman A, Enright AJ (2006) miRBase: microRNA sequences, targets and gene nomenclature. *Nucleic Acids Res* 34: D140–D144

Groh WJ, Groh MR, Saha C, Kincaid JC, Simmons Z, Ciafaloni E, Pourmand R, Otten RF, Bhakta D, Nair GV *et al* (2008) Electrocardiographic abnormalities and sudden death in myotonic dystrophy type 1. *N Engl J Med* 358: 2688–2697

de Haro M, Al-Ramahi I, De Gouyon B, Ukani L, Rosa A, Faustino NA, Ashizawa T, Cooper TA, Botas J (2006) MBNL1 and CUGBP1 modify expanded CUG-induced toxicity in a *Drosophila* model of myotonic dystrophy type 1. *Hum Mol Genet* 15: 2138–2145

de Haro M, Al-Ramahi I, Jones KR, Holth JK, Timchenko LT, Botas J (2013) Smaug/SAMD4A restores translational activity of CUGBP1 and suppresses CUG-induced myopathy. *PLoS Genet* 9: e1003445

Harpaz N, Ordan E, Ocorr K, Bodmer R, Volk T (2013) Multiplexin promotes heart but not aorta morphogenesis by polarized enhancement of slit/Robo activity at the heart lumen. *PLoS Genet* 9: e1003597

Hermans MC, Faber CG, Bekkers SC, de Die-Smulders CE, Gerrits MM, Merkies IS, Snoep G, Pinto YM, Schalla S (2012) Structural and functional cardiac changes in myotonic dystrophy type 1: a cardiovascular magnetic resonance study. *J Cardiovasc Magn Reson* 14: 48

Houseley JM, Wang Z, Brock GJR, Soloway J, Artero R, Perez-Alonso M, O'Dell KMC, Monckton DG (2005) Myotonic dystrophy associated expanded CUG

 © 2023 The Authors

repeat muscleblind positive ribonuclear foci are not toxic to *Drosophila*. *Hum Mol Genet* 14: 873 – 883

Huguet A, Medja F, Nicole A, Vignaud A, Guiraud-Dogan C, Ferry A, Decostre V, Hogrel J-Y, Metzger F, Hoeflich A *et al* (2012) Molecular, physiological, and motor performance defects in DMSXL mice carrying >1000 CTG repeats from the human DM1 locus. *PLoS Genet* 8: e1003043

Ikeda S, Kong SW, Lu J, Bisping E, Zhang H, Allen PD, Golub TR, Pieske B, Pu WT (2007) Altered microRNA expression in human heart disease. *Physiol Genomics* 31: 367 – 373

Isserlin R, Merico D, Wang D, Vuckovic D, Bousette N, Gramolini AO, Bader GD, Emili A (2014) Systems analysis reveals down-regulation of a network of pro-survival miRNAs drives the apoptotic response in dilated cardiomyopathy. *Mol Biosyst* 11: 239 – 251

Kalsotra A, Singh RK, Gurha P, Ward AJ, Creighton CJ, Cooper TA (2014) The Mef2 transcription network is disrupted in myotonic dystrophy heart tissue dramatically altering miRNA and mRNA expression. *Cell Rep* 6: 336 – 345

Katoh T, Hojo H, Suzuki T (2015) Destabilization of microRNAs in human cells by 3′ deadenylation mediated by PARN and CUGBP1. *Nucleic Acids Res* 43: 7521 – 7534

Koshelev M, Sarma S, Price RE, Wehrens XHT, Cooper TA (2010) Heart-specific overexpression of CUGBP1 reproduces functional and molecular abnormalities of myotonic dystrophy type 1. *Hum Mol Genet* 19: 1066 – 1075

Kuei-Ting C, Ching-Feng C, Pei-Chih K, Shin-Yi L, Guey-Shin W (2017) CELF1 mediates connexin 43 mRNA degradation in dilated cardiomyopathy. *Circ Res* 121: 1140 – 1152

Kura B, Kalocayova B, Devaux Y, Bartekova M (2020) Potential clinical implications of miR-1 and miR-21 in heart disease and cardioprotection. *Int J Mol Sci* 21: 700

Kuyumcu-Martinez NM, Wang G-S, Cooper TA (2007) Increased steady state levels of CUGBP1 in myotonic dystrophy 1 are due to PKC-mediated hyper-phosphorylation. *Mol Cell* 28: 68 – 78

Langmead B, Salzberg SL (2012) Fast gapped-read alignment with bowtie 2. *Nat Methods* 9: 357 – 359

Lee JE, Cooper TA (2009) Pathogenic mechanisms of myotonic dystrophy. *Biochem Soc Trans* 37: 1281 – 1286

Lee K-Y, Li M, Manchanda M, Batra R, Charizanis K, Mohan A, Warren SA, Chamberlain CM, Finn D, Hong H *et al* (2013) Compound loss of muscleblind-like function in myotonic dystrophy. *EMBO Mol Med* 5: 1887 – 1900

Lin AE, Mitchell FM, Fitz RW, Doyle JJ (1989) Dilated cardiomyopathy in myotonic dystrophy. *J Am Coll Cardiol* 13: 262 – 263

Louzao-Martinez L, Vink A, Harakalova M, Asselbergs FW, Verhaar MC, Cheng C (2016) Characteristic adaptations of the extracellular matrix in dilated cardiomyopathy. *Int J Cardiol* 220: 634 – 646

Masuda A, Andersen HS, Doktor TK, Okamoto T, Ito M, Andresen BS, Ohno K (2012) CUGBP1 and MBNL1 preferentially bind to 3′ UTRs and facilitate mRNA decay. *Sci Rep* 2: 209

Mathieu J, Allard P, Potvin L, Prévost C, Bégin P (1999) A 10-year study of mortality in a cohort of patients with myotonic dystrophy. *Neurology* 52: 1658 – 1662

McNally EM, Mestroni L (2017) Dilated cardiomyopathy. *Circ Res* 121: 731 – 748

Meola G, Cardani R (2015) Myotonic dystrophies: an update on clinical aspects, genetic, pathology, and molecular pathomechanisms. *Biochim Biophys Acta* 1852: 594 – 606

Meyer F, Moussian B (2009) *Drosophila* multiplexin (Dmp) modulates motor axon pathfinding accuracy. *Dev Growth Differ* 51: 483 – 498

Nakamori M, Kimura T, Kubota T, Matsumura T, Sumi H, Fujimura H, Takahashi MP, Sakoda S (2008) Aberrantly spliced alpha-dystrobrevin alters alpha-syntrophin binding in myotonic dystrophy type 1. *Neurology* 70: 677 – 685

Nakamori M, Gourdon G, Thornton CA (2011) Stabilization of expanded (CTG) •(CAG) repeats by antisense oligonucleotides. *Mol Ther* 19: 2222 – 2227

Nguyen HH, Wolfe JT, Holmes DR, Edwards WD (1988) Pathology of the cardiac conduction system in myotonic dystrophy: a study of 12 cases. *J Am Coll Cardiol* 11: 662 – 671

Ocorr K, Fink M, Cammarato A, Bernstein SI, Bodmer R (2009) Semi-automated optical heartbeat analysis of small hearts. *J Vis Exp* https://doi.org/10.3791/1435

Orengo JP, Chambon P, Metzger D, Mosier DR, Snipes GJ, Cooper TA (2008) Expanded CTG repeats within the DMPK 3′ UTR causes severe skeletal muscle wasting in an inducible mouse model for myotonic dystrophy. *Proc Natl Acad Sci USA* 105: 2646 – 2651

Papa AA, Verrillo F, Scutifero M, Rago A, Morra S, Cassese A, Cioppa ND, Magliocca MC, Galante D, Palladino A *et al* (2018) Heart transplantation in a patient with myotonic dystrophy type 1 and end-stage dilated cardiomyopathy: a short term follow-up. *Acta Myol* 37: 267 – 271

Pelargonio G, Russo AD, Sanna T, De Martino G, Bellocci F (2002) Myotonic dystrophy and the heart. *Heart* 88: 665 – 670

Philips AV, Timchenko LT, Cooper TA (1998) Disruption of splicing regulated by a CUG-binding protein in myotonic dystrophy. *Science* 280: 737 – 741

Picchio L, Plantie E, Renaud Y, Poovthumkadavil P, Jagla K (2013) Novel *Drosophila* model of myotonic dystrophy type 1: phenotypic characterization and genome-wide view of altered gene expression. *Hum Mol Genet* 22: 2795 – 2810

Picchio L, Legagneux V, Deschamps S, Renaud Y, Chauveau S, Paillard L, Jagla K (2018) Bruno-3 regulates sarcomere component expression and contributes to muscle phenotypes of myotonic dystrophy type 1. *Dis Model Mech* 11: dmm031849

Rao PK, Yumiko T, Rosaria CH, Sumeet G, Michael B, Rostislav M, Ferenc R, Ronglih L, Monty K, Rudolf J *et al* (2009) Loss of cardiac microRNA-mediated regulation leads to dilated cardiomyopathy and heart failure. *Circ Res* 105: 585 – 594

Rasi K, Jarkko P, Marcus C, Raija S, Mika I, Hanna L, Jaana R, Risto K, Paul J, Ritva H *et al* (2010) Collagen XV is necessary for modeling of the extracellular matrix and its deficiency predisposes to cardiomyopathy. *Circ Res* 107: 1241 – 1252

Rau F, Freyermuth F, Fugier C, Villemin J-P, Fischer M-C, Jost B, Dembele D, Gourdon G, Nicole A, Duboc D *et al* (2011) Misregulation of miR-1 processing is associated with heart defects in myotonic dystrophy. *Nat Struct Mol Biol* 18: 840 – 845

Schilling L, Forst R, Forst J, Fujak A (2013) Orthopaedic disorders in myotonic dystrophy type 1: descriptive clinical study of 21 patients. *BMC Musculoskelet Disord* 14: 338

Souidi A, Jagla K (2021) *Drosophila* heart as a model for cardiac development and diseases. *Cell* 10: 3078

Souidi A, Zmojdzian M, Jagla K (2018) Dissecting pathogenetic mechanisms and therapeutic strategies in *Drosophila* models of myotonic dystrophy type 1. *Int J Mol Sci* 19: E4104

Timchenko NA, Wang G-L, Timchenko LT (2005) RNA CUG-binding protein 1 increases translation of 20-kDa isoform of CCAAT/enhancer-binding protein β by interacting with the α and β subunits of eukaryotic initiation translation factor 2. *J Biol Chem* 280: 20549 – 20557

Timchenko LT, Salisbury E, Wang G-L, Nguyen H, Albrecht JH, Hershey JWB, Timchenko NA (2006) Age-specific CUGBP1-eIF2 complex increases translation of CCAAT/enhancer-binding protein beta in old liver. *J Biol Chem* 281: 32806 – 32819

© 2023 The Authors

Vlasova IA, Tahoe NM, Fan D, Larsson O, Rattenbacher B, Sternjohn JR, Vasdewani J, Karypis G, Reilly CS, Bitterman PB *et al* (2008) Conserved GU-rich elements mediate mRNA decay by binding to CUG-binding protein 1. *Mol Cell* 29: 263 – 270

Wang G-S, Kearney DL, De Biasi M, Taffet G, Cooper TA (2007) Elevation of RNA-binding protein CUGBP1 is an early event in an inducible heart-specific mouse model of myotonic dystrophy. *J Clin Invest* 117: 2802 – 2811

Wang ET, Ward AJ, Cherone JM, Giudice J, Wang TT, Treacy DJ, Lambert NJ, Freese P, Saxena T, Cooper TA *et al* (2015) Antagonistic regulation of mRNA expression and splicing by CELF and MBNL proteins. *Genome Res* 25: 858 – 871

Wei Y, Peng S, Wu M, Sachidanandam R, Tu Z, Zhang S, Falce C, Sobie EA, Lebeche D, Zhao Y (2014) Multifaceted roles of miR-1 s in repressing the fetal gene program in the heart. *Cell Res* 24: 278 – 292

Wolf MJ (2012) Modeling dilated cardiomyopathies in *Drosophila*. *Trends Cardiovasc Med* 22: 55 – 61

License: This is an open access article under the terms of the Creative Commons Attribution License, which permits use, distribution and reproduction in any medium, provided the original work is properly cited.

Expanded View Figures

Figure EV1. Flies showing DCM present myofibrillar disarray in the heart tubes.

A–D′ Z stack projection from hearts of controls (*UAS-mblRNAi*, *UAS-Bru3*, *UAS-dmiR-1 sponge* and *UAS-Mp*) (A–D) and mutants (*Hand* > *mblRNAi*, *Hand* > *Bru3*, *Hand* > *dmiR-1 sponge* and *Hand* > *Mp*) (A′–D′) aged of 5 weeks. The arrows point to irregularity in myofibrillar density and arrangement in the heart tubes. Scale bar = 20 μm.

E–G Box plots showing cardiac size analyses (diastolic (E) and systolic (F) diameters) and percent fractional shortening (G) performed by SOHA approach for controls (*Hand-Gal4* and *UAS-960CTG*) and mutant (*Hand* > *960CTG*) at 1 and 5 weeks of age. Aged *Hand* > *960CTG* flies present cardiac dilation characterized by a significant increase in diastolic and systolic diameters in comparison with control *Hand* > *Gal4* (E, F). $n = 20$ hearts. Central band corresponds to median. Whiskers correspond to Min to Max. Box corresponds to interquartile range from 25th to 75th percentile.

Data information: P-value < 0.05 considered statistically significant. (**) P-value = 0.021, (***) P-value = 0.0002. The nonparametric Mann–Whitney test was performed to compare control samples and samples of interest.

© 2023 The Authors

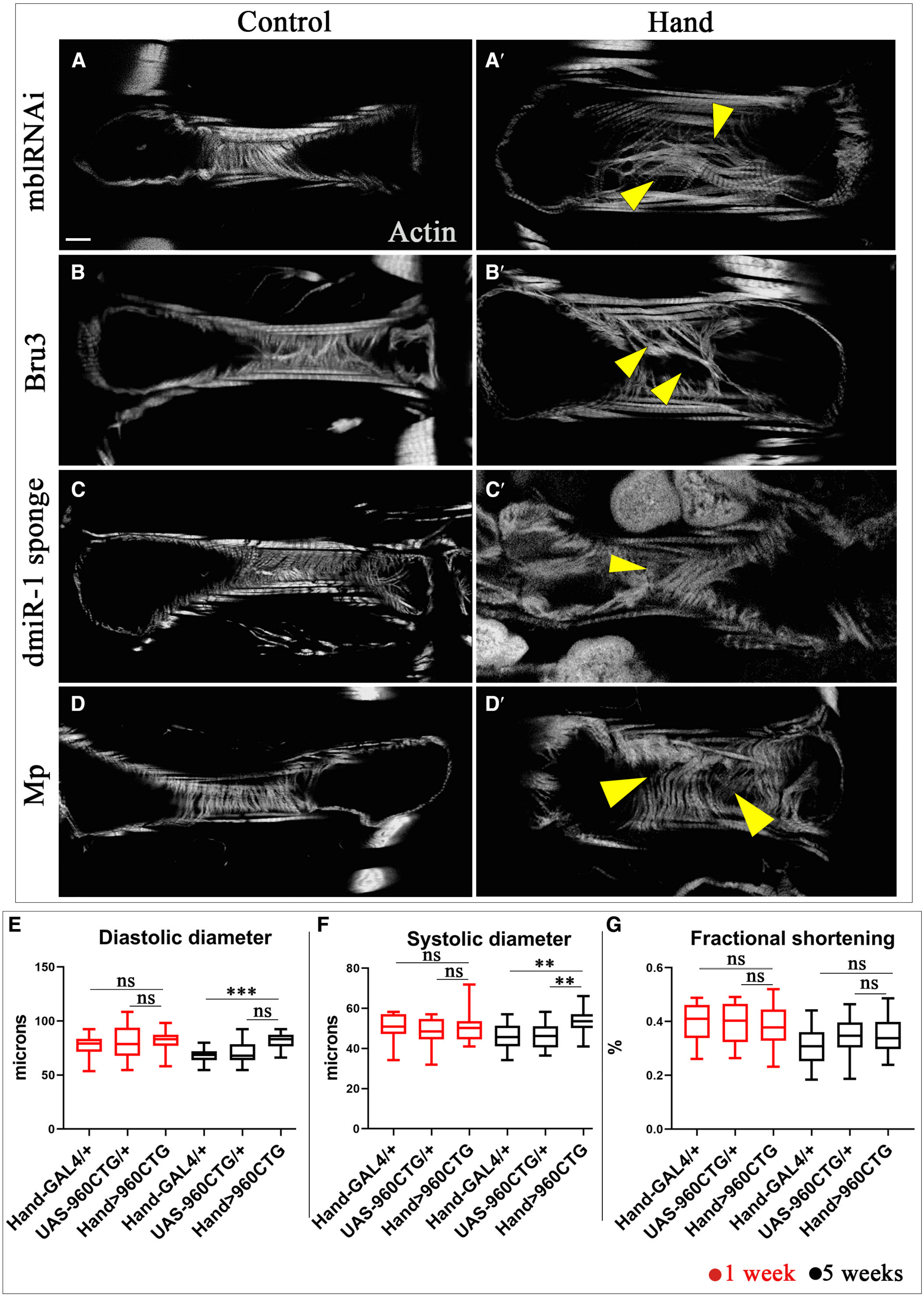

Figure EV1.

 © 2023 The Authors

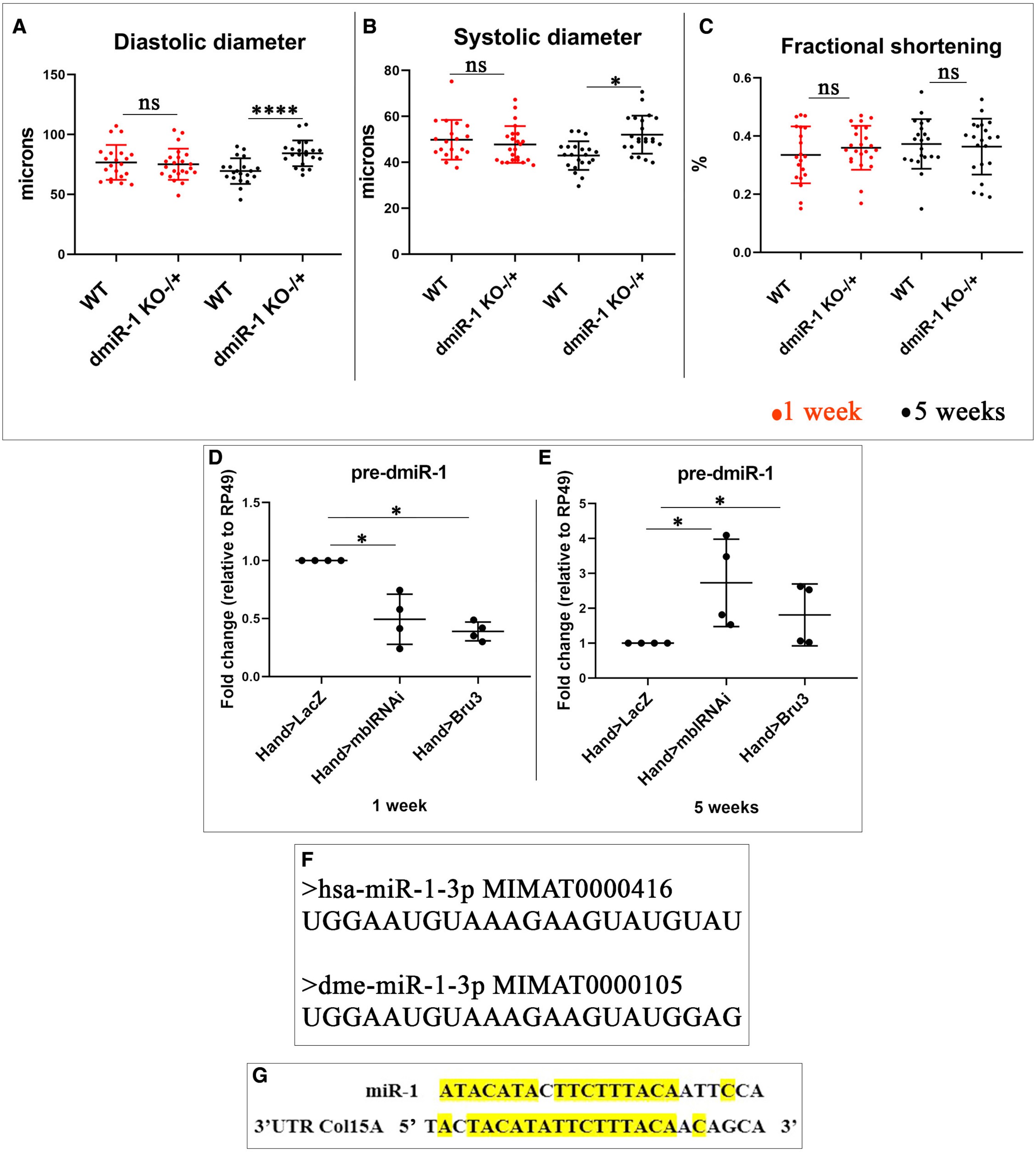
A
Diastolic diameter
microns
150
100
50
0
ns
****
WT
dmiR-1 KO-/+
WT
dmiR-1 KO-/+
B
Systolic diameter
microns
80
60
40
20
0
ns
*
WT
dmiR-1 KO-/+
WT
dmiR-1 KO-/+
C
Fractional shortening
%
0.6
0.4
0.2
0.0
ns
ns
WT
dmiR-1 KO-/+
WT
dmiR-1 KO-/+
•1 week
•5 weeks
D
pre-dmiR-1
Fold change (relative to RP49)
1.5
1.0
0.5
0.0
*
*
Hand>LacZ
Hand>mblRNAi
Hand>Bru3
1 week
E
pre-dmiR-1
Fold change (relative to RP49)
5
4
3
2
1
0
*
*
Hand>LacZ
Hand>mblRNAi
Hand>Bru3
5 weeks
F
>hsa-miR-1-3p MIMAT0000416
UGGAAUGUAAAGAAGUAUGUAU
>dme-miR-1-3p MIMAT0000105
UGGAAUGUAAAGAAGUAUGGAG
G
miR-1 ATACATACTTCTTTACAATTCCA
3'UTR Col15A 5' TACTACATATTCTTTACAACAGCA 3'

Figure EV2.

Figure EV2. Supplemental analyses of *dmiR-1*.

A–C Scatter plot showing cardiac size analyses (diastolic (A) and systolic (B) diameters) and percent fractional shortening (C) performed by SOHA approach for control wt^{1118} and heterozygous mutant context (*dmiR-1 KO*$^{-/+}$) at 1 and 5 weeks of age. Aged *Hand* > *dmiR-1 KO*$^{-/+}$ flies present cardiac dilation characterized by a significant increase in diastolic and systolic diameters in comparison with control. $n = 20$ hearts. Bars correspond to mean ± SD.

D, E RTq–PCR analysis for *pre-dmiR-1* transcript in adult heart of 1 and 5 weeks of age for control (*Hand* > *LacZ*) and DM1 contexts (*Hand* > *mblRNAi*, *Hand* > *Bru3*). $n = 4$ biological replicates.

F Alignment of *Drosophila* and human *miR-1* sequences.

G Potential binding site of human *miR-1* in 3′UTR of *COL15A1* (*COL15A1–201* transcript position 4428 to 4447).

Data information: *P*-value < 0.05 considered statistically significant. (*) *P*-value = 0.033, (****) *P*-value < 0.0001. The nonparametric Mann–Whitney test was performed to compare control samples and samples of interest.

Figure EV3. DM1 flies show reduced levels of *dmiR-1* in cardiac cells.

A–H′ FISH for scramble (5′ 3′ DIG) (A′) and U6 (5′DIG) (B′) detected in ω^{1118} cardiac tube. FISH with miRCURY LNA probe for *dmiR-1* detection in controls (*UAS-dmiR-1*, *UAS-mblRNAi*, *UAS-Bru3*) (C′, E′, G′) and mutants (*Hand* > *dmiR-1*, *Hand* > *mblRNAi*, *Hand* > *Bru3*) (D′, F′, H′) cardiac tubes labeled with *dmiR-1* probes (red) and actin (green) at 1 week of age. Representative spot views generated using Imaris from *in situ* hybridization with miRCURY LNA probe for *dmiR-1* and used for quantification of *dmiR-1* levels.

I, I′ Spot views of *dmiR-1* in hearts of 1-week-old control (*UAS-mblRNAi*) (I) and DM1 (*Hand* > *mblRNAi*) flies (I′) are shown. Each spot represents a pool of *dmiR-1* transcripts detected in the same area. The zoom area in (I) and (I′) corresponds to pericardial cells.

J, K Scatter plot graph showing the signal intensity of *dmiR-1* levels quantified in pericardial cells of 1- and 5-week-old flies for controls (*UAS-Bru3*, *UAS-mblRNAi*) and DM1 contexts (*Hand* > *Bru3*, *Hand* > *mblRNAi*). $n = 27$ pericardial cells. Bars correspond to mean ± SD.

Data information: Scale bar = 40 μm. *P*-value < 0.05 considered statistically significant. (*) *P*-value = 0.033, (**) *P*-value = 0.021, (***) *P*-value = 0.0002, (****) *P*-value < 0.0001. The nonparametric Mann–Whitney test was performed to compare control samples and samples of interest.

 © 2023 The Authors

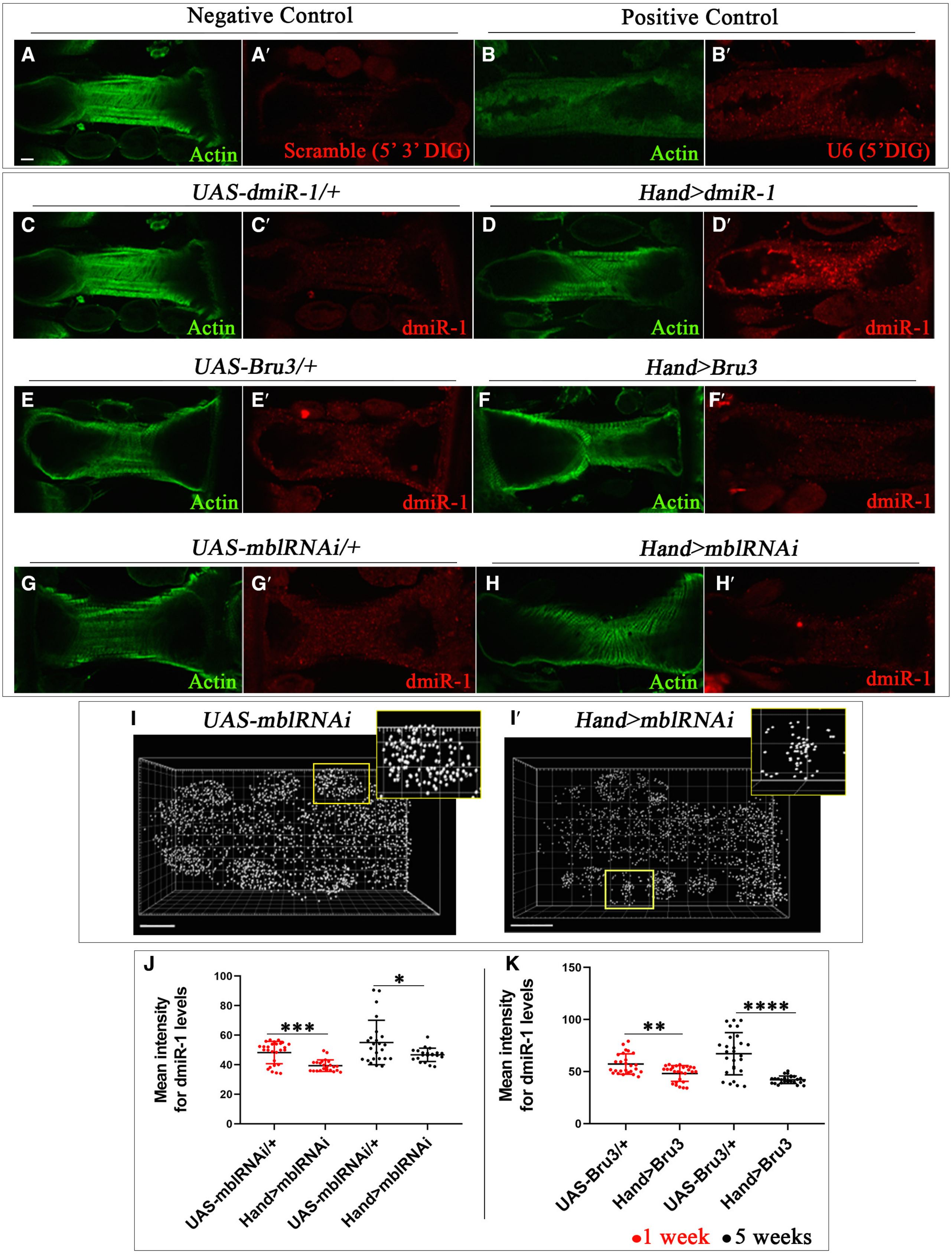
Negative Control
Positive Control
A
A′
B
B′
Actin
Scramble (5' 3' DIG)
Actin
U6 (5'DIG)
UAS-dmiR-1/+
Hand>dmiR-1
C
C′
D
D′
Actin
dmiR-1
Actin
dmiR-1
UAS-Bru3/+
Hand>Bru3
E
E′
F
F′
Actin
dmiR-1
Actin
dmiR-1
UAS-mblRNAi/+
Hand>mblRNAi
G
G′
H
H′
Actin
dmiR-1
Actin
dmiR-1
I
UAS-mblRNAi
I′
Hand>mblRNAi
J
Mean intensity for dmiR-1 levels
100
80
60
40
20
0
***
*
UAS-mblRNAi/+
Hand>mblRNAi
UAS-mblRNAi/+
Hand>mblRNAi
K
Mean intensity for dmiR-1 levels
150
100
50
0
**
****
UAS-Bru3/+
Hand>Bru3
UAS-Bru3/+
Hand>Bru3
•1 week •5 weeks

Figure EV3.

Figure EV4. Mp is expressed in the adult fly heart and up-regulated in pericardial cells of DCM-developing DM1 lines.

A–C″ (A) Adult heart of ω^{1118} line labeled for Mp (green), actin (red), and DAPI (blue). (C) The yellow arrows indicate ventral longitudinal muscles (VLM). (C′) The yellow arrows indicate circular fibers (within cardioblast cells). (A″, B″) Cross-section of ω^{1118} cardiac tube after 3D reconstruction with Imaris software, showing the expression of Mp at the internal and the external surface of the cardiac tube.

D–G′ Adult heart of 1 and 5 weeks of age labeled for Mp (green) for controls (*UAS-mblRNAi*, *UAS-Bru3*) (D–G) and DM1 context (*Hand > mblRNAi*, *Hand > Bru3*) (D′–G′). Encircled region in E′ corresponds to an example of area used for quantifications of the fluorescent signal in pericardial cells using the CTCF method.

H, I Fluorescence signal intensity quantification for Mp expression in pericardial cells in adult heart of 1 and 5 weeks of age for controls (*UAS-mblRNAi*, *UAS-Bru3*) and DM1 context (*Hand > mblRNAi*, *Hand > Bru3*) using the CTCF method. For each genotype, 9 hearts were analyzed and 3 pericardial cells were analyzed from each heart. Bars correspond to mean ± SD.

J Fluorescence signal intensity quantification for Mp expression in cardioblasts in adult heart of 1 week of age for control (*UAS-dmiR-1 sponge*) and mutant (*Hand > dmiR-1 sponge*) using the CTCF method. For each genotype, 9 hearts were analyzed and fluorescence intensity was measured in two regions from segment A3 and two regions from segment A4. Bars correspond to mean ± SD.

Data information: Scale bar = 20 μm. *P*-value < 0.05 considered statistically significant. (**) *P*-value = 0.021, (***) *P*-value = 0.0002, (****) *P*-value < 0.0001. The nonparametric Mann–Whitney test was performed to compare control samples and samples of interest.

 © 2023 The Authors

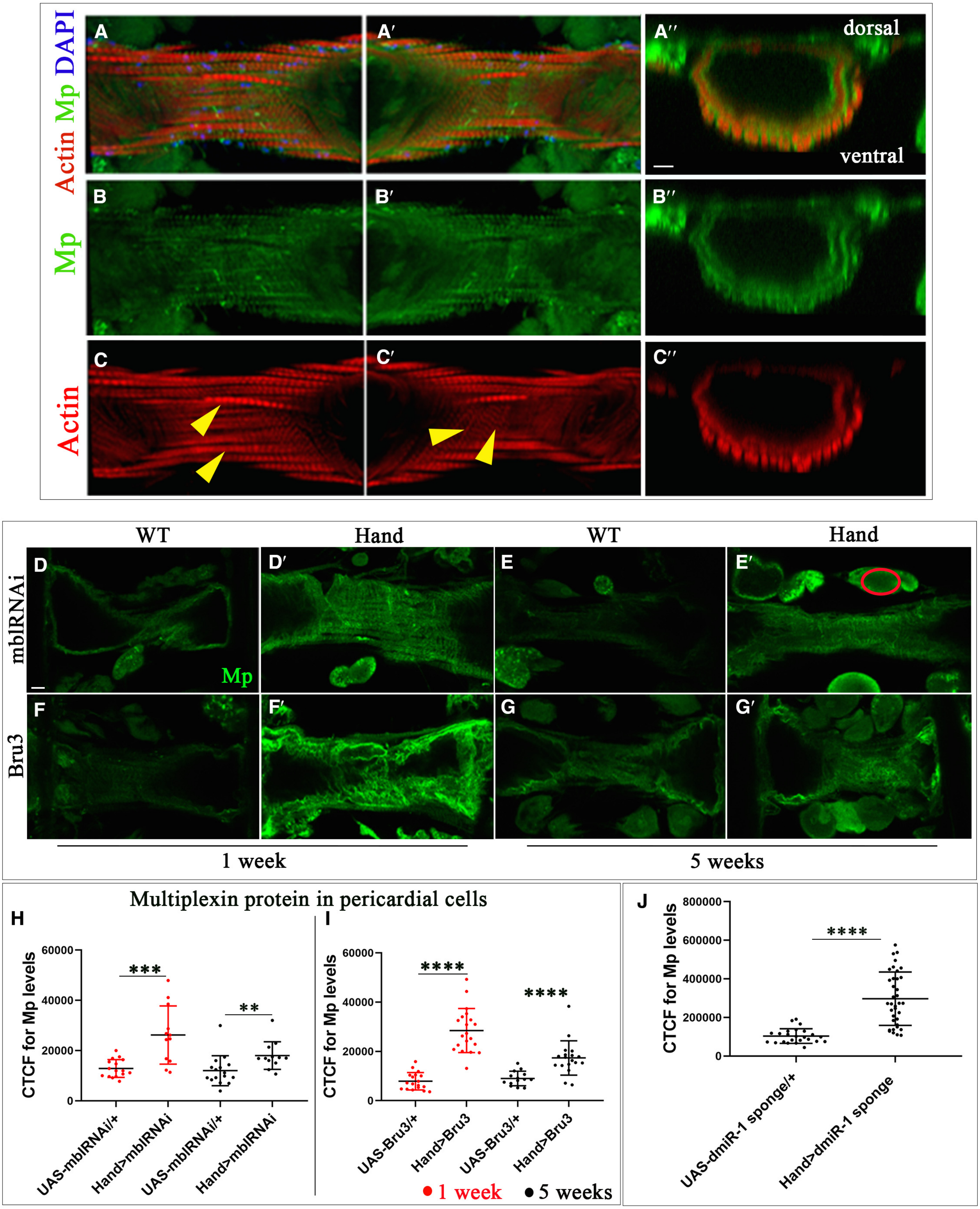
Actin Mp DAPI
A
A′
A″
dorsal
ventral
Mp
B
B′
B″
Actin
C
C′
C″
WT
Hand
WT
Hand
mblRNAi
Bru3
D
D′
E
E′
F
F′
G
G′
Mp
1 week
5 weeks
Multiplexin protein in pericardial cells
H
CTCF for Mp levels
60000
40000
20000
0
***
**
UAS-mblRNAi/+
Hand>mblRNAi
UAS-mblRNAi/+
Hand>mblRNAi
I
CTCF for Mp levels
60000
40000
20000
0
****
****
UAS-Bru3/+
Hand>Bru3
UAS-Bru3/+
Hand>Bru3
● 1 week
● 5 weeks
J
CTCF for Mp levels
800000
600000
400000
200000
0
****
UAS-dmiR-1 sponge/+
Hand>dmiR-1 sponge

Figure EV4.

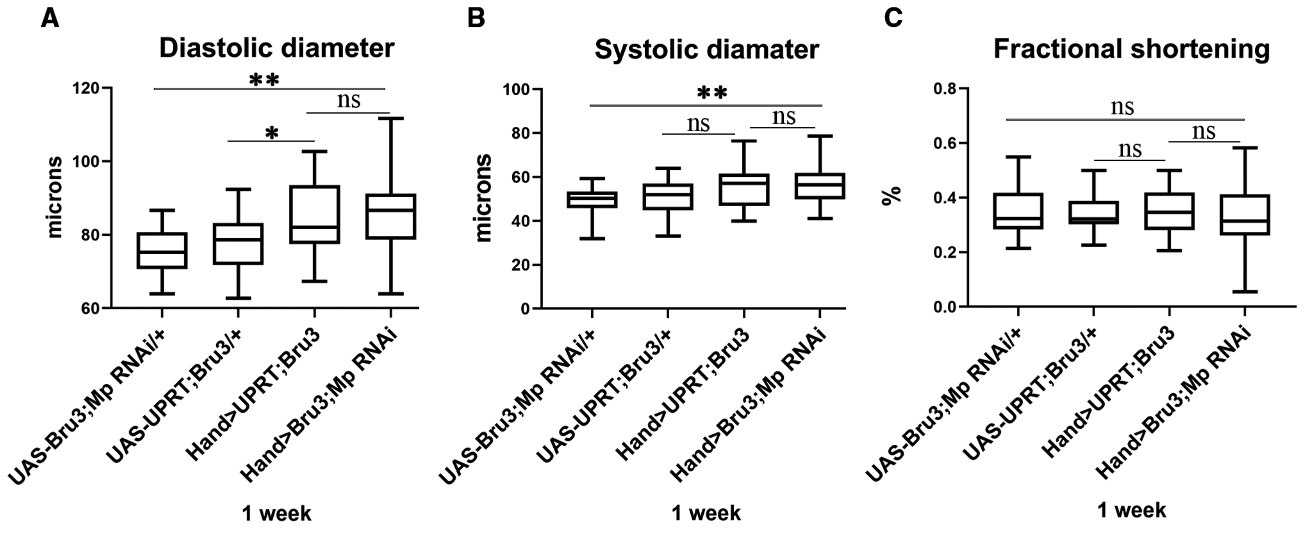

Figure EV5. The young DM1 flies with reduced Mp present normal heart size.

A–C Box plots showing cardiac size analyses (diastolic (A) and systolic (B) diameters) and percent fractional shortening (C) performed by SOHA approach for controls (*UAS-Bru3; UAS-MpRNAi* and *UAS-UPRT; Bru3*) and Mp rescue (*Hand > Bru3; Mp RNAi*) and DM1 (*Hand > UPRT; Bru3*) at 1 week of age. $n = 20$ hearts. Central band corresponds to median. Whiskers correspond to Min to Max. Box corresponds to interquartile range from 25th to 75th percentile.

Data information: P-value < 0.05 considered statistically significant. (*) P-value = 0.033, (**) P-value = 0.021. The nonparametric Mann–Whitney test was performed to compare control samples and samples of interest.

 © 2023 The Authors
