# Supplementary material for: Deregulations of miR‐1 and its target Multiplexin promote dilated cardiomyopathy associated with myotonic dystrophy type 1
Source: EMBO Rep. 2023 Feb 28;24(4):e56616. doi: 10.15252/embr.202256616 (PMC10074075; doi:10.15252/embr.202256616)
Supplement: Supplementary file 4 — Source Data for Figure 1 [file EMBR-24-e56616-s001.zip › embr202256616-sup-0003-SDataFig1/EMBOR-2022-56616V2-Figure_1_Readme-sd.docx]

(A) Maximum intensity projection of Z-stack images of the adult heart of *UAS-dmiR-1 sponge* line

(A’) Cross-section of the 3D-reconstructed adult cardiac tube represented in (A)

(B) Maximum intensity projection of Z-stack images of the adult heart of *Hand>dmiR-1 sponge* line

(B’) Cross-section of the 3D-reconstructed adult cardiac tube represented in (B)

(C) M-mode generated by SOHA program from the heart beat movie of *UAS-dmiR-1 sponge* line

(C’) M-mode generated by SOHA program from the heart beat movie of *Hand>dmiR-1 sponge* line

(D) Heart diameters in the end of relaxation (maximum diastole) for *UAS-dmiR-1 sponge* and *Hand>dmiR-1 sponge* flies, obtained by SOHA program

(E) Heart diameters in the end of contraction (maximum systole) for *UAS-dmiR-1 sponge* and *Hand>dmiR-1 sponge* flies, obtained by SOHA

(F) Fractional shortening measurements represent the contractility of the heart of *UAS-dmiR-1 sponge* and *Hand>dmiR-1 sponge* flies, calculated by SOHA program
