# Supplementary figures and images for: Deregulations of miR‐1 and its target Multiplexin promote dilated cardiomyopathy associated with myotonic dystrophy type 1
Source: EMBO Rep. 2023 Feb 28;24(4):e56616. doi: 10.15252/embr.202256616 (PMC10074075; doi:10.15252/embr.202256616)

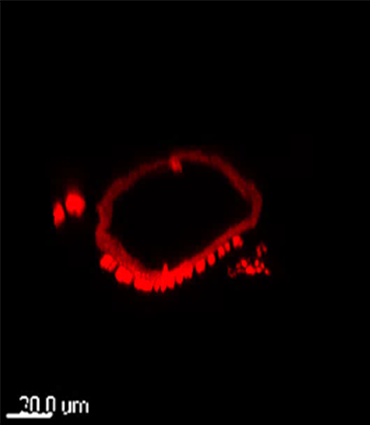

Supplement: Supplementary file 4 — Source Data for Figure 1 [file EMBR-24-e56616-s001.zip › embr202256616-sup-0003-SDataFig1/EMBOR-2022-56616V2-Fig1A-sd(1).tif]

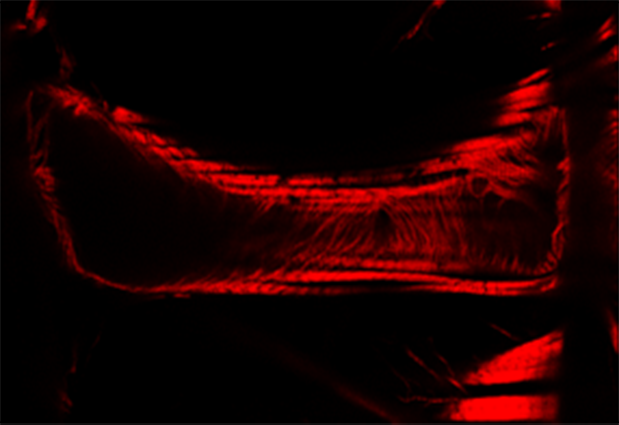

Supplement: Supplementary file 4 — Source Data for Figure 1 [file EMBR-24-e56616-s001.zip › embr202256616-sup-0003-SDataFig1/EMBOR-2022-56616V2-Fig1A-sd.tif]

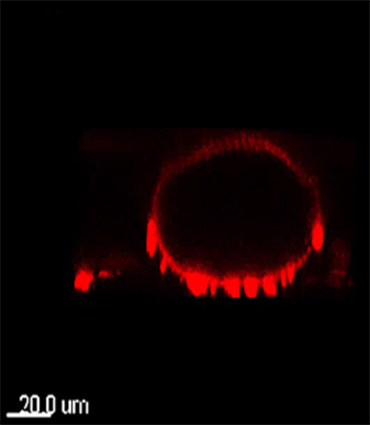

Supplement: Supplementary file 4 — Source Data for Figure 1 [file EMBR-24-e56616-s001.zip › embr202256616-sup-0003-SDataFig1/EMBOR-2022-56616V2-Fig1B-sd(1).tif]

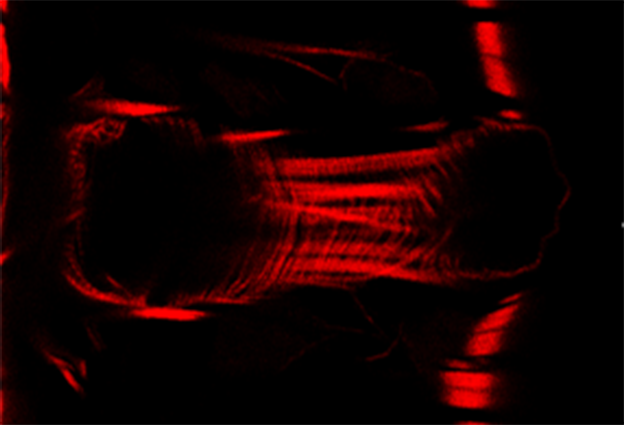

Supplement: Supplementary file 4 — Source Data for Figure 1 [file EMBR-24-e56616-s001.zip › embr202256616-sup-0003-SDataFig1/EMBOR-2022-56616V2-Fig1B-sd.tif]

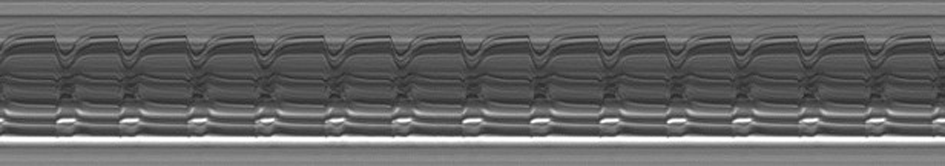

Supplement: Supplementary file 4 — Source Data for Figure 1 [file EMBR-24-e56616-s001.zip › embr202256616-sup-0003-SDataFig1/EMBOR-2022-56616V2-Fig1C-sd(1).tif]

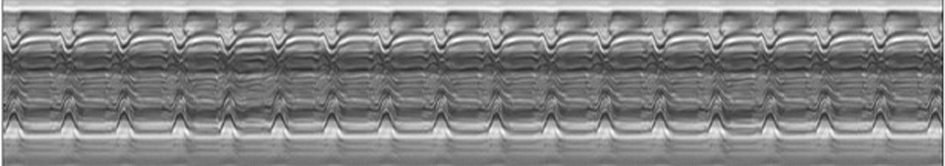

Supplement: Supplementary file 4 — Source Data for Figure 1 [file EMBR-24-e56616-s001.zip › embr202256616-sup-0003-SDataFig1/EMBOR-2022-56616V2-Fig1C-sd.tif]

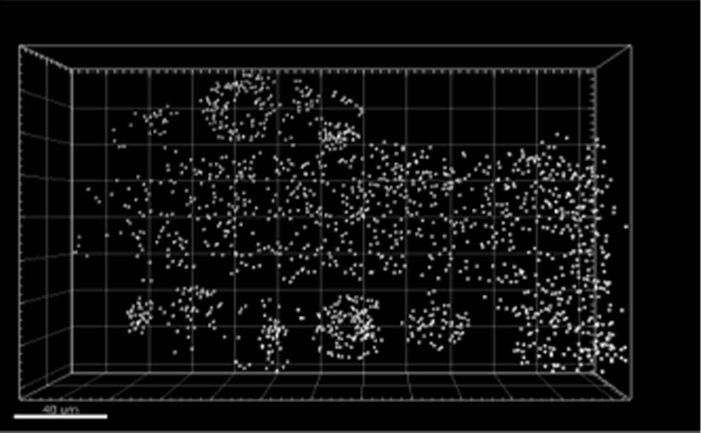

Supplement: Supplementary file 5 — Source Data for Figure 2 [file EMBR-24-e56616-s006.zip › embr202256616-sup-0004-SDataFig2/EMBOR-2022-56616V2-Fig2G-sd(1).tif]

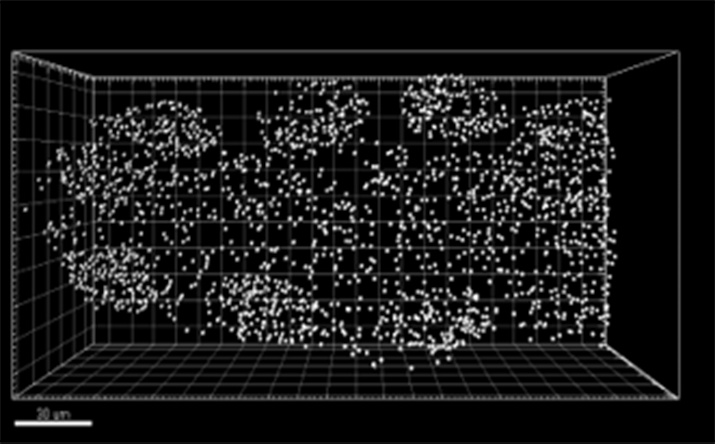

Supplement: Supplementary file 5 — Source Data for Figure 2 [file EMBR-24-e56616-s006.zip › embr202256616-sup-0004-SDataFig2/EMBOR-2022-56616V2-Fig2G-sd.tif]

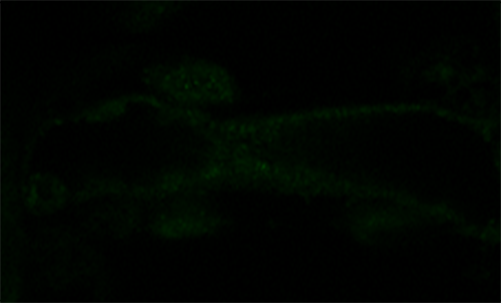

Supplement: Supplementary file 6 — Source Data for Figure 3 [file EMBR-24-e56616-s003.zip › embr202256616-sup-0005-SDataFig3/EMBOR-2022-56616V2-Fig3C-sd(1).tif]

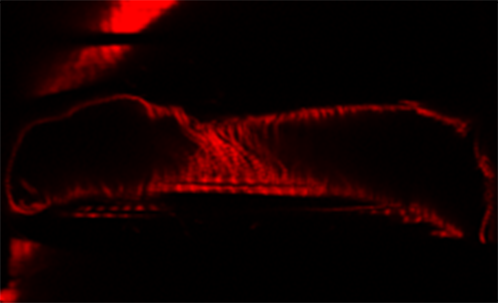

Supplement: Supplementary file 6 — Source Data for Figure 3 [file EMBR-24-e56616-s003.zip › embr202256616-sup-0005-SDataFig3/EMBOR-2022-56616V2-Fig3C-sd.tif]

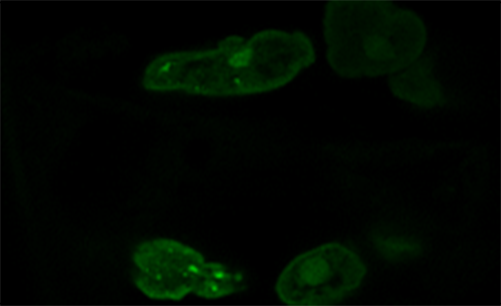

Supplement: Supplementary file 6 — Source Data for Figure 3 [file EMBR-24-e56616-s003.zip › embr202256616-sup-0005-SDataFig3/EMBOR-2022-56616V2-Fig3D-sd(1).tif]

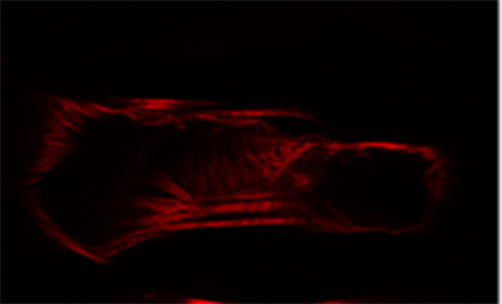

Supplement: Supplementary file 6 — Source Data for Figure 3 [file EMBR-24-e56616-s003.zip › embr202256616-sup-0005-SDataFig3/EMBOR-2022-56616V2-Fig3D-sd.tif]

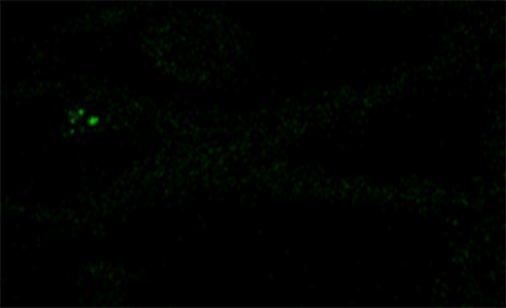

Supplement: Supplementary file 6 — Source Data for Figure 3 [file EMBR-24-e56616-s003.zip › embr202256616-sup-0005-SDataFig3/EMBOR-2022-56616V2-Fig3E-sd(1).tif]

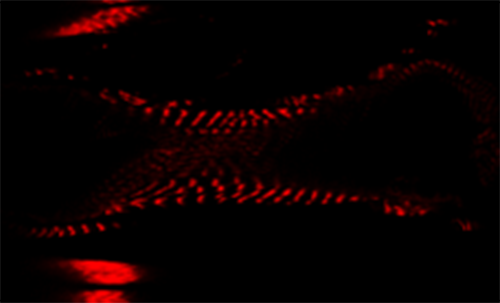

Supplement: Supplementary file 6 — Source Data for Figure 3 [file EMBR-24-e56616-s003.zip › embr202256616-sup-0005-SDataFig3/EMBOR-2022-56616V2-Fig3E-sd.tif]

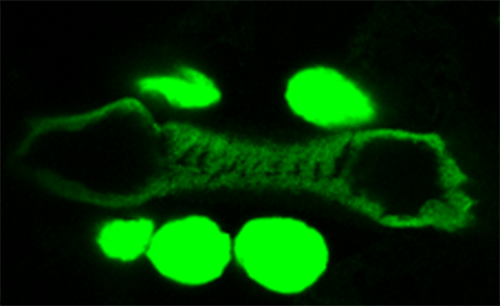

Supplement: Supplementary file 6 — Source Data for Figure 3 [file EMBR-24-e56616-s003.zip › embr202256616-sup-0005-SDataFig3/EMBOR-2022-56616V2-Fig3F-sd(1).tif]

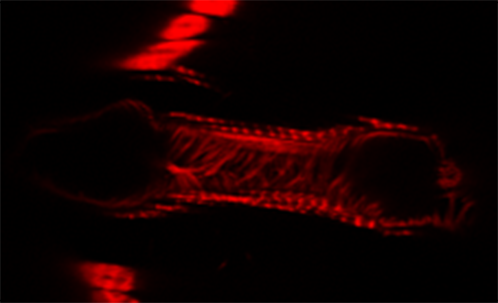

Supplement: Supplementary file 6 — Source Data for Figure 3 [file EMBR-24-e56616-s003.zip › embr202256616-sup-0005-SDataFig3/EMBOR-2022-56616V2-Fig3F-sd.tif]

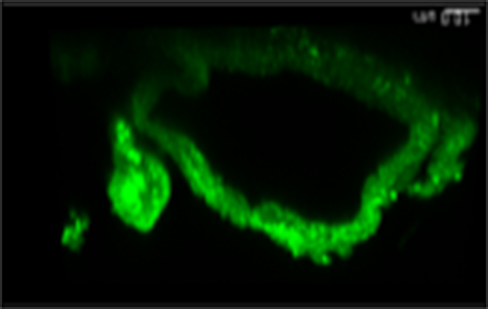

Supplement: Supplementary file 6 — Source Data for Figure 3 [file EMBR-24-e56616-s003.zip › embr202256616-sup-0005-SDataFig3/EMBOR-2022-56616V2-Fig3G-sd(1).tif]

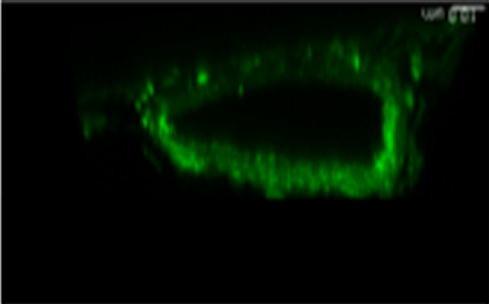

Supplement: Supplementary file 6 — Source Data for Figure 3 [file EMBR-24-e56616-s003.zip › embr202256616-sup-0005-SDataFig3/EMBOR-2022-56616V2-Fig3G-sd.tif]

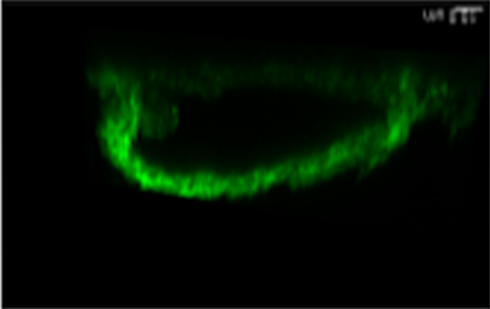

Supplement: Supplementary file 6 — Source Data for Figure 3 [file EMBR-24-e56616-s003.zip › embr202256616-sup-0005-SDataFig3/EMBOR-2022-56616V2-Fig3H-sd(1).tif]

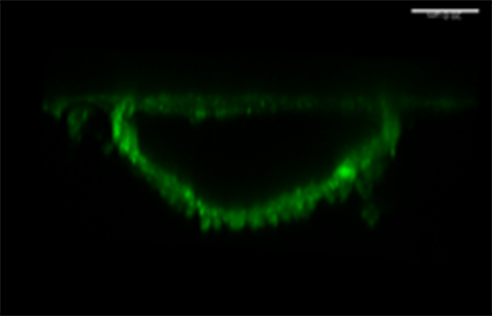

Supplement: Supplementary file 6 — Source Data for Figure 3 [file EMBR-24-e56616-s003.zip › embr202256616-sup-0005-SDataFig3/EMBOR-2022-56616V2-Fig3H-sd.tif]

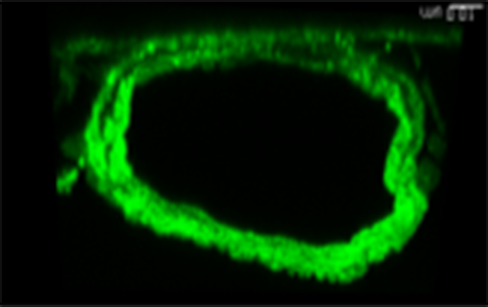

Supplement: Supplementary file 6 — Source Data for Figure 3 [file EMBR-24-e56616-s003.zip › embr202256616-sup-0005-SDataFig3/EMBOR-2022-56616V2-Fig3I-sd(1).tif]

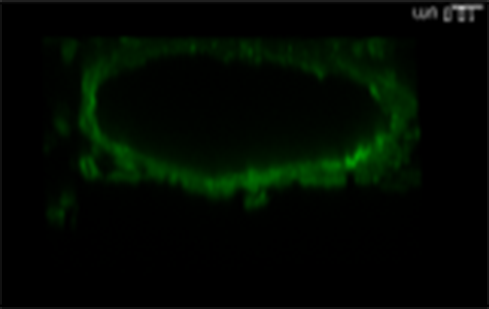

Supplement: Supplementary file 6 — Source Data for Figure 3 [file EMBR-24-e56616-s003.zip › embr202256616-sup-0005-SDataFig3/EMBOR-2022-56616V2-Fig3I-sd.tif]

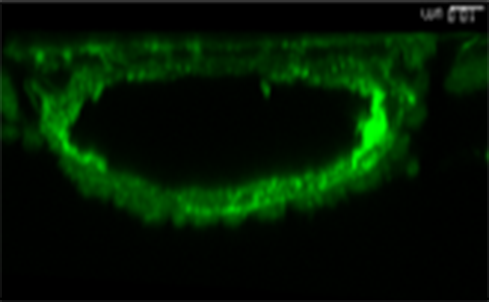

Supplement: Supplementary file 6 — Source Data for Figure 3 [file EMBR-24-e56616-s003.zip › embr202256616-sup-0005-SDataFig3/EMBOR-2022-56616V2-Fig3J-sd(1).tif]

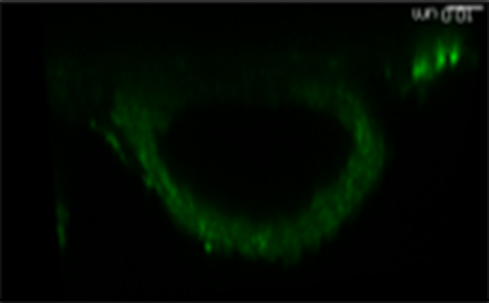

Supplement: Supplementary file 6 — Source Data for Figure 3 [file EMBR-24-e56616-s003.zip › embr202256616-sup-0005-SDataFig3/EMBOR-2022-56616V2-Fig3J-sd.tif]

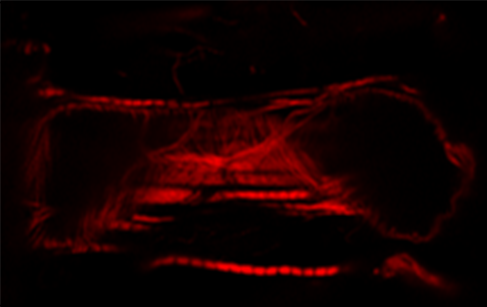

Supplement: Supplementary file 7 — Source Data for Figure 4 [file EMBR-24-e56616-s008.zip › embr202256616-sup-0006-SDataFig4/EMBOR-2022-56616V2-Fig4A-sd(1).tif]

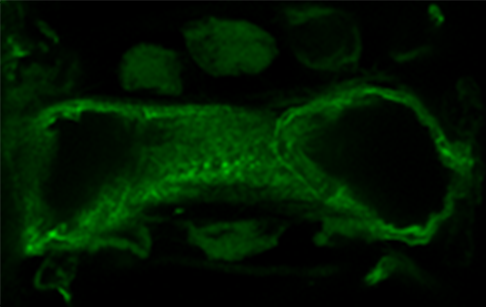

Supplement: Supplementary file 7 — Source Data for Figure 4 [file EMBR-24-e56616-s008.zip › embr202256616-sup-0006-SDataFig4/EMBOR-2022-56616V2-Fig4A-sd.tif]

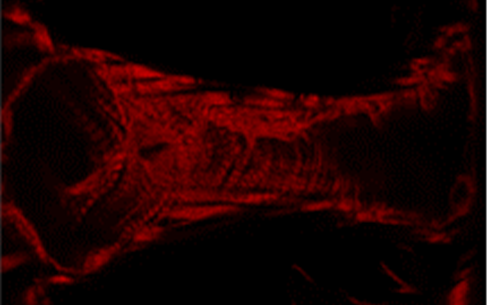

Supplement: Supplementary file 7 — Source Data for Figure 4 [file EMBR-24-e56616-s008.zip › embr202256616-sup-0006-SDataFig4/EMBOR-2022-56616V2-Fig4B-sd(1).tif]

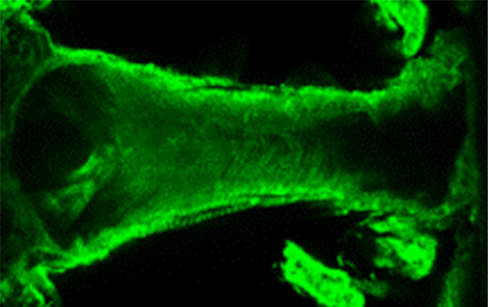

Supplement: Supplementary file 7 — Source Data for Figure 4 [file EMBR-24-e56616-s008.zip › embr202256616-sup-0006-SDataFig4/EMBOR-2022-56616V2-Fig4B-sd.tif]

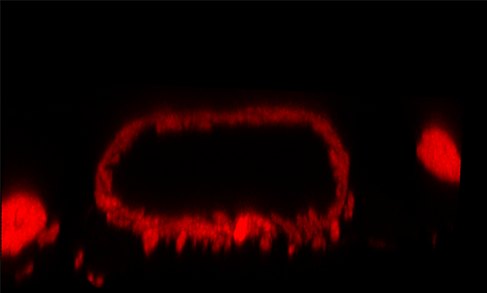

Supplement: Supplementary file 7 — Source Data for Figure 4 [file EMBR-24-e56616-s008.zip › embr202256616-sup-0006-SDataFig4/EMBOR-2022-56616V2-Fig4C-sd(1).tif]

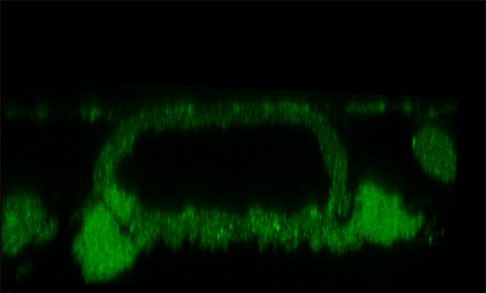

Supplement: Supplementary file 7 — Source Data for Figure 4 [file EMBR-24-e56616-s008.zip › embr202256616-sup-0006-SDataFig4/EMBOR-2022-56616V2-Fig4C-sd.tif]

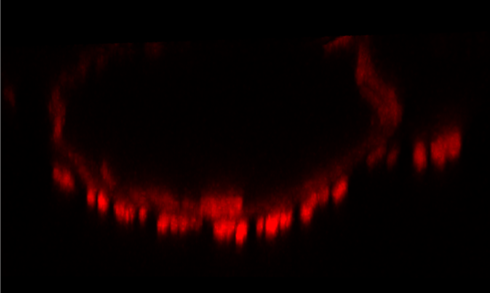

Supplement: Supplementary file 7 — Source Data for Figure 4 [file EMBR-24-e56616-s008.zip › embr202256616-sup-0006-SDataFig4/EMBOR-2022-56616V2-Fig4D-sd(1).tif]

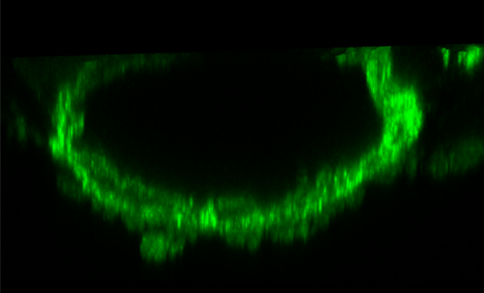

Supplement: Supplementary file 7 — Source Data for Figure 4 [file EMBR-24-e56616-s008.zip › embr202256616-sup-0006-SDataFig4/EMBOR-2022-56616V2-Fig4D-sd.tif]

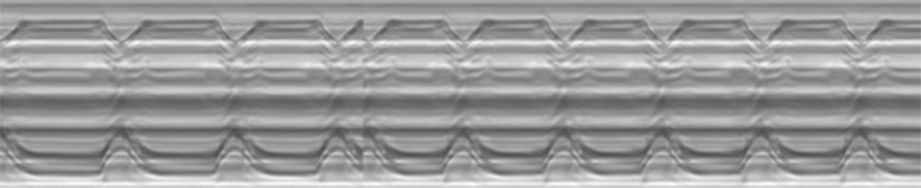

Supplement: Supplementary file 7 — Source Data for Figure 4 [file EMBR-24-e56616-s008.zip › embr202256616-sup-0006-SDataFig4/EMBOR-2022-56616V2-Fig4H-sd(1).tif]

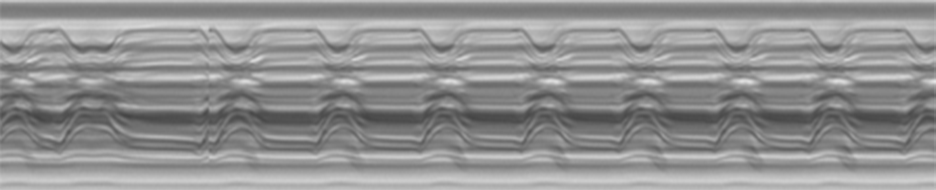

Supplement: Supplementary file 7 — Source Data for Figure 4 [file EMBR-24-e56616-s008.zip › embr202256616-sup-0006-SDataFig4/EMBOR-2022-56616V2-Fig4H-sd.tif]

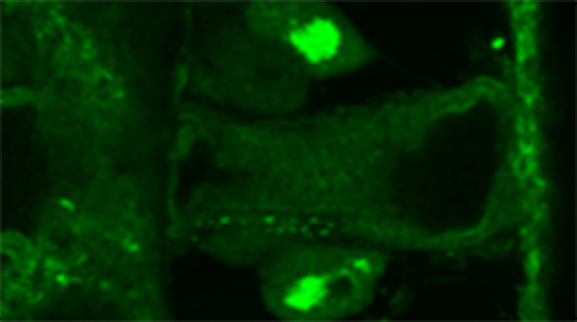

Supplement: Supplementary file 8 — Source Data for Figure 5 [file EMBR-24-e56616-s010.zip › embr202256616-sup-0007-SDataFig5/EMBOR-2022-56616V2-Fig5A-sd(1).tif]

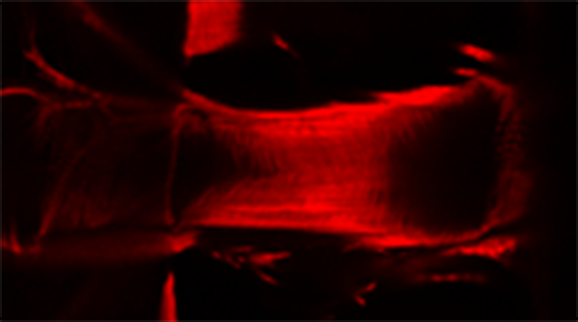

Supplement: Supplementary file 8 — Source Data for Figure 5 [file EMBR-24-e56616-s010.zip › embr202256616-sup-0007-SDataFig5/EMBOR-2022-56616V2-Fig5A-sd.tif]

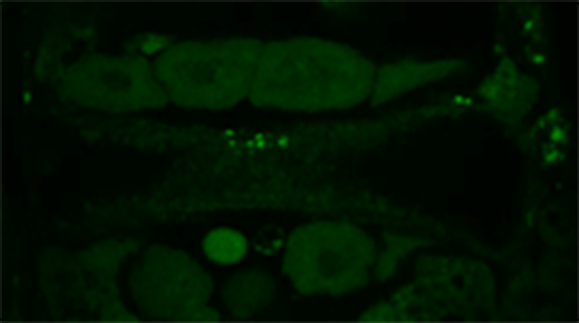

Supplement: Supplementary file 8 — Source Data for Figure 5 [file EMBR-24-e56616-s010.zip › embr202256616-sup-0007-SDataFig5/EMBOR-2022-56616V2-Fig5B-sd(1).tif]

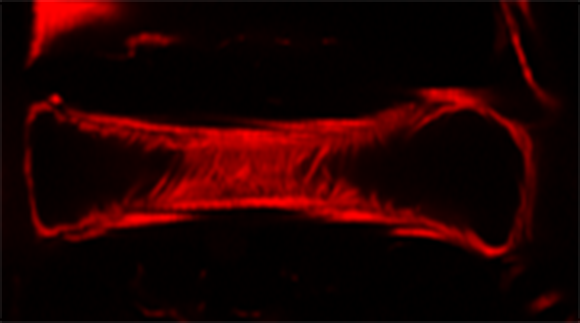

Supplement: Supplementary file 8 — Source Data for Figure 5 [file EMBR-24-e56616-s010.zip › embr202256616-sup-0007-SDataFig5/EMBOR-2022-56616V2-Fig5B-sd.tif]

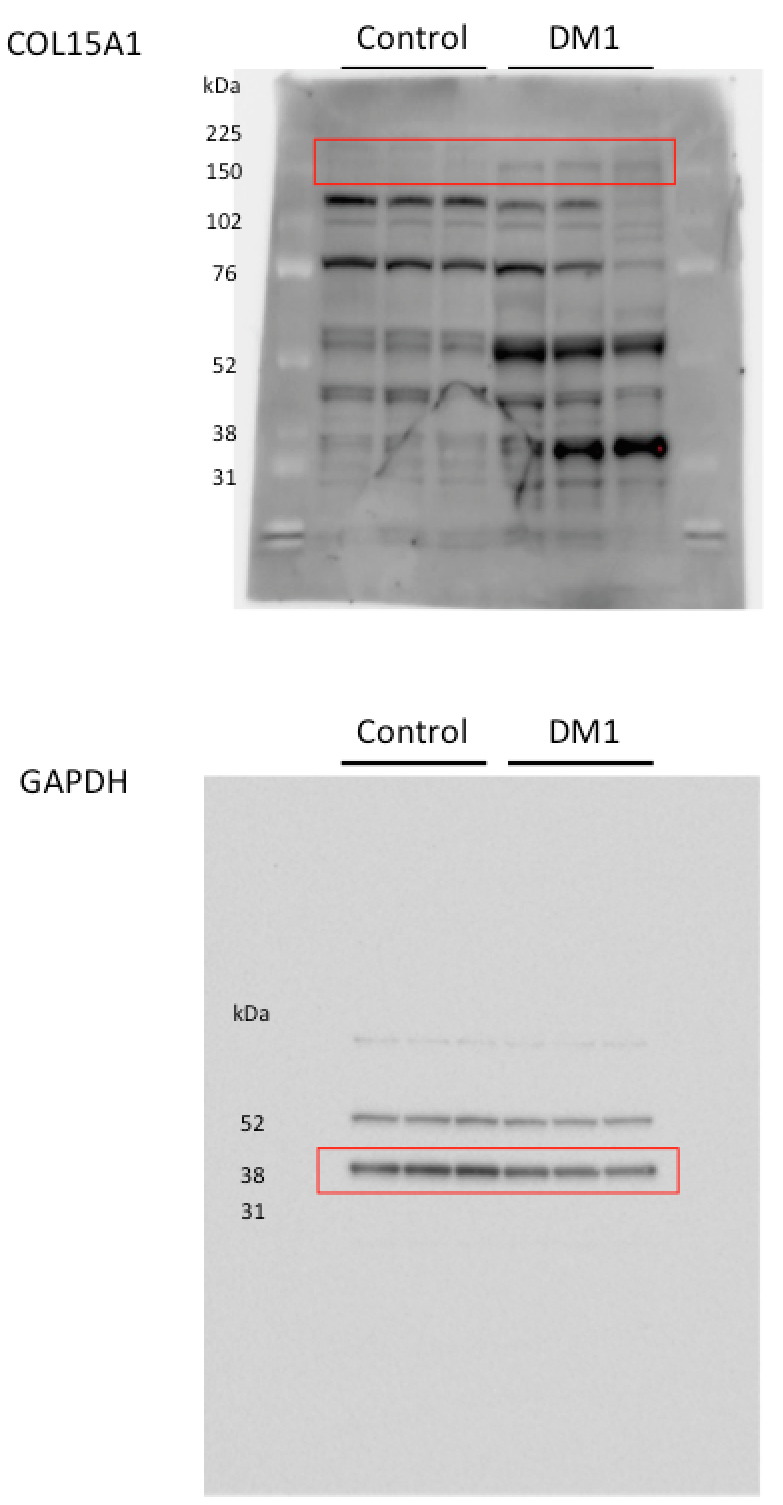

Supplement: Supplementary file 9 — Source Data for Figure 6 [file EMBR-24-e56616-s005.zip › embr202256616-sup-0008-SDataFig6/EMBOR-2022-56616V2-Fig6B_Western-sd.tif]

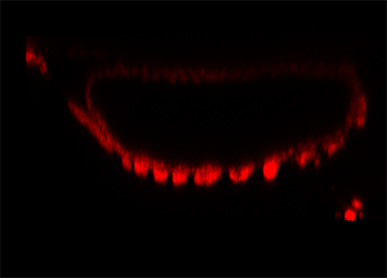

Supplement: Supplementary file 9 — Source Data for Figure 6 [file EMBR-24-e56616-s005.zip › embr202256616-sup-0008-SDataFig6/EMBOR-2022-56616V2-Fig6G-sd.tif]

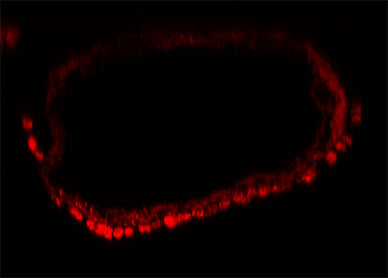

Supplement: Supplementary file 9 — Source Data for Figure 6 [file EMBR-24-e56616-s005.zip › embr202256616-sup-0008-SDataFig6/EMBOR-2022-56616V2-Fig6H-sd.tif]

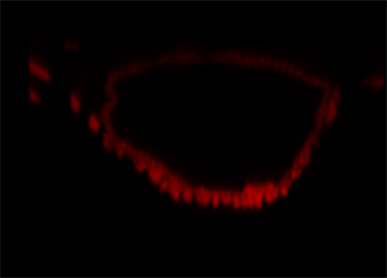

Supplement: Supplementary file 9 — Source Data for Figure 6 [file EMBR-24-e56616-s005.zip › embr202256616-sup-0008-SDataFig6/EMBOR-2022-56616V2-Fig6I-sd.tif]
