# Supplementary material for: Deregulations of miR‐1 and its target Multiplexin promote dilated cardiomyopathy associated with myotonic dystrophy type 1
Source: EMBO Rep. 2023 Feb 28;24(4):e56616. doi: 10.15252/embr.202256616 (PMC10074075; doi:10.15252/embr.202256616)
Supplement: Supplementary file 5 — Source Data for Figure 2 [file EMBR-24-e56616-s006.zip › embr202256616-sup-0004-SDataFig2/EMBOR-2022-56616V2-Figure_2_Readme-sd.docx]

(A) Heart diameters in the end of relaxation (maximum diastole) for *UAS-Bru3* and *Hand>Bru3* obtained by SOHA program

(B) Heart diameters in the end of contraction (maximum systole) for *UAS-Bru3* and *Hand>Bru3* obtained by SOHA program

(C) Fractional shortening measurements represent the contractility of the *UAS-Bru3* and *Hand>Bru3* hearts calculated by SOHA program

(D) Heart diameters in the end of relaxation (maximum diastole) for *UAS-mblRNAi* and *Hand>mblRNAi* obtained by SOHA program

(E) Heart diameters in the end of contraction (maximum systole) for *UAS-mblRNAi* and *Hand>mblRNAi* obtained by SOHA program

(F) Fractional shortening measurements represent the contractility of the *UAS-mblRNAi* and *Hand>mblRNAi* hearts calculated by SOHA program

(G) Spot views generated by Imaris software in 3D reconstructed adult hearts (*UAS-mblRNAi*) after background subtracting. Single spot observed in the figure corresponds to *dmiR-1* transcripts labeled with miRCURY LNA probe for *dmiR-1*

(G’) Spot views generated by Imaris software in 3D reconstructed adult hearts (*Hand>mblRNAi*) after background subtracting. Single spot observed in the figure corresponds to *dmiR-1* transcripts labeled with miRCURY LNA probe for *dmiR-1*

(H) Each spot in the scatter plot graph corresponds to the average of the mean intensities calculated by Imaris software for all *dmiR-1* spots detected in cardiomyocytes of one adult heart (*UAS-mblRNAi* or Hand>*mblRNAi*) after background subtracting

(H’) Each spot in the scatter plot graph corresponds to the average of the mean intensities calculated by Imaris software for all *dmiR-1* spots detected in cardiomyocytes of one adult heart (*UAS-Bru3* or Hand>*Bru3*) after background subtracting
