# Supplementary material for: Deregulations of miR‐1 and its target Multiplexin promote dilated cardiomyopathy associated with myotonic dystrophy type 1
Source: EMBO Rep. 2023 Feb 28;24(4):e56616. doi: 10.15252/embr.202256616 (PMC10074075; doi:10.15252/embr.202256616)
Supplement: Supplementary file 6 — Source Data for Figure 3 [file EMBR-24-e56616-s003.zip › embr202256616-sup-0005-SDataFig3/EMBOR-2022-56616V2-Figure_3_Readme-sd.docx]

(C) Maximum intensity projection of Z-stack images of the *UAS-GFP3’UTRMp; UAS-miR1*adult heart labeled for Actin

(C’) Maximum intensity projection of Z-stack images of the *UAS-GFP3’UTRMp; UAS-miR1*adult heart labeled for GFP

(D) Maximum intensity projection of Z-stack images of the *Hand>-GFP3’UTRMp; UAS-miR1*adult heart labeled for Actin

(D’) Maximum intensity projection of Z-stack images of the *Hand>-GFP3’UTRMp; UAS-miR1*adult heart labeled for GFP

(E) Maximum intensity projection of Z-stack images of the *UAS-GFPdelta3’UTRMp; UAS-miR1*adult heart labeled for Actin

(E’) Maximum intensity projection of Z-stack images of the *UAS-GFPdelta3’UTRMp; UAS-miR1*adult heart labeled for GFP

(F) Maximum intensity projection of Z-stack images of the *Hand>GFPdelta3’UTRMp; UAS-miR1*adult heart labeled for Actin

(F’) Maximum intensity projection of Z-stack images of the *Hand>GFPdelta3’UTRMp; UAS-miR1*adult heart labeled for GFP

(G, H) Cross-sections of the 3D-reconstructed adult *UAS-mblRNAi* cardiac tube labeled for Mp

(G’, H’) Cross-sections of the 3D-reconstructed adult *Hand>mblRNAi* cardiac tube labeled for Mp

(I, J) Cross-sections of the 3D-reconstructed adult *UAS-Bru3* cardiac tube labeled for Mp

(I’, J’) Cross-sections of the 3D-reconstructed adult *Hand>Bru3* cardiac tube labeled for Mp
