# Supplementary material for: Deregulations of miR‐1 and its target Multiplexin promote dilated cardiomyopathy associated with myotonic dystrophy type 1
Source: EMBO Rep. 2023 Feb 28;24(4):e56616. doi: 10.15252/embr.202256616 (PMC10074075; doi:10.15252/embr.202256616)
Supplement: Supplementary file 7 — Source Data for Figure 4 [file EMBR-24-e56616-s008.zip › embr202256616-sup-0006-SDataFig4/EMBOR-2022-56616V2-Figure_4_Readme-sd.docx]

(A) Maximum intensity projection of Z-stack images of the adult heart of *UAS-Mp* line labeled with Mp

(A’) Maximum intensity projection of Z-stack images of the adult heart of *UAS-Mp* line labeled with actin

(B) Maximum intensity projection of Z-stack images of the adult heart of *Hand>Mp* line labeled with Mp

(B’) Maximum intensity projection of Z-stack images of the adult heart of *Hand>Mp* line labeled with actin

(C) Cross-section of the 3D-reconstructed adult cardiac tube represented in (A)

(C’) Cross-section of the 3D-reconstructed adult cardiac tube represented in (A’)

(D) Cross-section of the 3D-reconstructed adult cardiac tube represented in (B)

(D’) Cross-section of the 3D-reconstructed adult cardiac tube represented in (B’)

(E) Heart diameters in the end of relaxation (maximum diastole) for *UAS-Mp* and *Hand>Mp* flies, obtained by SOHA program

(F) Heart diameters in the end of contraction (maximum systole) for *UAS-Mp* and *Hand>Mp* flies, obtained by SOHA program

(G) Fractional shortening measurements represent the contractility of the heart of *UAS-Mp* and *Hand>Mp* flies, calculated by SOHA program

(H) M-mode generated by SOHA program from the heart beat movie of *UAS-Mp* line

(H’) M-mode generated by SOHA program from the heart beat movie of *Hand>Mp* line
