# Supplementary material for: Deregulations of miR‐1 and its target Multiplexin promote dilated cardiomyopathy associated with myotonic dystrophy type 1
Source: EMBO Rep. 2023 Feb 28;24(4):e56616. doi: 10.15252/embr.202256616 (PMC10074075; doi:10.15252/embr.202256616)
Supplement: Supplementary file 8 — Source Data for Figure 5 [file EMBR-24-e56616-s010.zip › embr202256616-sup-0007-SDataFig5/EMBOR-2022-56616V2-Figure_5_Readme-sd.docx]

(A) Maximum intensity projection of Z-stack images of the adult heart of *UAS-Mp RNAi* lines labeled with actin

(A’) Maximum intensity projection of Z-stack images of the adult heart of *UAS-Mp RNAi* lines labeled with Mp

(B) Maximum intensity projection of Z-stack images of the adult heart of *UAS-Mp RNAi* line labeled with actin

(B’) Maximum intensity projection of Z-stack images of the adult heart of *Hand>Mp RNAi* line labeled with Mp

(D) Heart diameters in the end of relaxation (maximum diastole) for *UAS-Mp RNAi* and *Hand>Mp RNAi* flies, obtained by SOHA program

(E) Heart diameters in the end of contraction (maximum systole) for *UAS-Mp RNAi* and *Hand>Mp RNAi* flies, obtained by SOHA program

(F) Fractional shortening measurements represent the contractility of the heart of *UAS-Mp RNAi* and *Hand>Mp RNAi* flies, calculated by SOHA program
