# Supplementary material for: Deregulations of miR‐1 and its target Multiplexin promote dilated cardiomyopathy associated with myotonic dystrophy type 1
Source: EMBO Rep. 2023 Feb 28;24(4):e56616. doi: 10.15252/embr.202256616 (PMC10074075; doi:10.15252/embr.202256616)
Supplement: Supplementary file 9 — Source Data for Figure 6 [file EMBR-24-e56616-s005.zip › embr202256616-sup-0008-SDataFig6/EMBOR-2022-56616V2-Figure_6_Readme-sd.docx]

(A) Relative expression of *Col15A1* transcripts quantified by RT-qPCR in ventricular cardiac muscle tissues of DM1 patients with dilated cardiomyopathy (DCM+) and without dilated cardiomyopathy (DCM-) and from controls, normalized to ribosomal RNA 18S.

(B) Western blots of crude protein extracts from 3 control tissue samples of cardiac muscles and from 3 samples of cardiac muscle of DM1 patients. Antibody against COL15A1 and antibody against GAPDH were used for the detection of COL15A1 and GAPDH respectively. Proteins are detected by Touch Imaging System (Bio-Rad). GAPDH served as a loading control.

(C) Relative *miR-1* expression quantified by RT-qPCR in ventricular cardiac muscle tissues from DM1 patients and from controls, normalized to miR-16.

(D) Heart diameters in the end of relaxation (maximum diastole) for *UAS-Bru3;Mp RNAi*, *UAS-UPRT;Bru3* and *Hand>Bru3;Mp RNAi*, *Hand>UPRT;Bru3* flies, obtained by SOHA program

(E) Heart diameters in the end of contraction (maximum systole) *UAS-Bru3;Mp RNAi*, *UAS-UPRT;Bru3* and *Hand>Bru3;Mp RNAi*, *Hand>UPRT;Bru3* flies, obtained by SOHA program

(F) Fractional shortening measurements represent the contractility of the heart of *UAS-Bru3;Mp RNAi,* *UAS-UPRT;Bru3* and *Hand>Bru3;Mp RNAi*, *Hand>UPRT;Bru3* flies, calculated by SOHA program

(G) Cross-section of the 3D-reconstructed adult *UAS-Bru3;Mp RNAi* cardiac tube labeled with actin

(H) Cross-section of the 3D-reconstructed adult *Hand>UPRT;Bru3* cardiac tube labeled with actin

(I) Cross-section of the 3D-reconstructed adult *Hand-Bru3;Mp RNAi* cardiac tube labeled with actin
