# Supplementary material for: Deregulations of miR‐1 and its target Multiplexin promote dilated cardiomyopathy associated with myotonic dystrophy type 1
Source: EMBO Rep. 2023 Feb 28;24(4):e56616. doi: 10.15252/embr.202256616 (PMC10074075; doi:10.15252/embr.202256616)
Supplement: Supplementary file 10 [file EMBR-24-e56616-s009.pdf]

EMBO reports

Deregulations of miR-1 and its target Multiplexin promote dilated cardiomyopathy associated with myotonic dystrophy type 1

Anissa Souidi, Masayuki Nakamori, Monika Zmojdzian, Teresa Jagla, Yoan RENAUD, and Krzysztof Jagla
DOI: 10.15252/embr.202050852

Corresponding author(s): Krzysztof Jagla (christophe.jagla@uca.fr)

Review Timeline:

Transfer from Review Commons:	6th Dec 22
Editorial Decision:	24th Jan 23
Revision Received:	3rd Feb 23
Accepted:	14th Feb 23

Editor: Esther Schnapp

Transaction Report: This manuscript was transferred to EMBO Reports following peer review at Review Commons.

(Note: With the exception of the correction of typographical or spelling errors that could be a source of ambiguity, letters and reports are not edited. Depending on transfer agreements, referee reports obtained elsewhere may or may not be included in this compilation. Referee reports are anonymous unless the Referee chooses to sign their reports.)

Review #1

1. Evidence, reproducibility and clarity:

Evidence, reproducibility and clarity (Required)

The paper titled "Deregulations of miR-1 and its target Multiplexin promote dilated cardiomyopathy associated with myotonic dystrophy type 1" by the Jagla group studied the effect of down-regulation of miR-1 in myotonic dystrophy type 1 (DM1) using fly as the disease model. The study is based on previous findings that in DM1 MBNL1 is sequestered, CELF1 is stabilized, and miR-1 is down regulated. The authors further identified Multiplexin to be the target effector of miR-1 in the fly heart and studied its function with a series of gain- and loss-of-function and rescue experiments. The authors' findings represent a significant advance in understanding the genetic mechanisms that can explain the pathogenic causes of dilated cardiomyopathy associated with DM1. Overall, this paper is well written and organized, with well-designed experiments and a clear model. A few additional experiments are suggested to further strengthen the conclusion.

Major points:

1. Wild-type and Hand-Gal4 controls are missing for all experiments (only UAS lines outcrossed to w1118 are displayed). Also, Hand-Gal4 driver lines can cause mild dilation by itself, which would influence interpretation and statistics of data. Also, the authors should consider to confirm the miR-1 phenotype obtained with the sponge with a miR mutant, and also combine miR-1 het with miR sponge (worsening of phenotype?). Alternatively, knockdown efficiency of miR should be tested by qPCR or HCR/smFISH.

2. It is surprising that one of the DM1 fly models, overexpression of 960 CTG repeats, did not show DCM, considering it is the primary cause of DM1 in humans due to excessive CTG repeats. It should be discussed why Hand>960 CTG does not lead to DCM, since the authors claim that this model with high number of CTG repeats shows a strong phenotype. Are Hand>bru3 and Hand>mbl stronger? Is miR-1 (and Mp) unaltered in these flies with 960 CTG repeats? It would be interesting to overexpress 960 CTG in a miR-1 or mbl heterozygous mutant background, which may produce DCM.

3. In Figure 2H, the mean intensity is displayed as the readout of the smFISH quantification of miR-1 levels. If understood correctly, this is the wrong readout since smFISH detects single molecule fluorescence of transcripts, so the number of transcripts should be quantified. Furthermore, Hand-Gal4 is not expressed in the ventral longitudinal muscles (VLM). As a proof of principle, miR-1 levels should be quantified in VLM (no change in transcripts levels expected).

4. Fig. 5: Since DCM- DM1 patients still show elevated COL15A1 levels but no DCM, it would be interesting to know if DCM phenotypes are COL15A1-dosage dependent. This could be easily tested in the fly model by testing UAS-Mp overexpression at different temperatures.

5. The authors elegantly show rescue of Hand>Bru3 flies by Mp RNAi. Their model would be further strengthened if a similar rescue can be shown with Hand>mblRNAi.

Minor points:

1. Fig. 1A,B: Ventral longitudinal muscles are covering the hearts on these images, so it's difficult to see the heart dimensions. This holds true for images throughout the manuscript. Where were the diameters measured (by the valves)? A better description and illustration would help the reader understand the situation.

2. Fig. 1 A',B': White line does not reflect the location where SOHA data are measured and should be horizontal for consistency. Where is ventral vs. dorsal?

3. Fig. 1D-F: Annotate 1 and 5 weeks in Figure, please. Also, why were 1 and 5 weeks tested? Is there an age-component in DM1 phenotype severity?

4. Fig. 3A: Transcriptional analysis was done at which stage of development?

5. Fig. 3: It is not clear, in which set the authors looked for miR-1 bindings sites (144 genes or the whole set)? Not well annotated. What is meant by 'heart-targeted'?

6. Fig. 4C,D: It looks like they are not shown in the same dorsal-ventral orientation. Also, it looks Mp is overexpressed in the VLM, but Hand-Gal4 only drives in the cardiomyocytes and pericardial cells? How was quantification done?

7. Fig. 4I: Why are some myofibers indicated in red in the model?

8. Fig. 5 D-E: Genotypes need to be better indicated in the graphs. Did the authors control for multiple UAS sites? Is UPRT a UAS control?

In the first paragraph of Result 3, the last sentence seems unfinished. "We identified a set of candidate genes, of which Multiplexin (Mp)"

In Method, the in silico screening for miR-1 target should be explained in more detail.

2. Significance:

Significance (Required)

The presented data is a significant advance our knowledge of our understanding of the molecular mechanisms involved in DM1. I expect that scientists in the muscular disease field and beyond will find this work of high interest.

3. How much time do you estimate the authors will need to complete the suggested revisions:

Estimated time to Complete Revisions (Required)

(Decision Recommendation)

Between 1 and 3 months

4. *Review Commons* values the work of reviewers and encourages them to get credit for their work. Select 'Yes' below to register your reviewing activity at Web of Science Reviewer Recognition Service (formerly Publons); note that the content of your review will not be visible on Web of Science.

Web of Science Reviewer Recognition

Yes

Review #2

1. Evidence, reproducibility and clarity:

Evidence, reproducibility and clarity (Required)

Summary:

This well-written manuscript utilizes the Drosophila model system to demonstrate that reduction of micro-RNA miR-1 and the resulting increase in one of its down-regulated proteins (Multiplexin) contribute to a dilated cardiomyopathy (DCM) phenotype. This is of interest in that this particular micro-RNA is downregulated in myotonic dystrophy type 1 (DM1), and this correlates with the DCM phenotype observed in patients. Further, the authors show that the human ortholog of Multiplexin is enriched in human DM1-DCM hearts and that downregulation of this protein in Drosophila DM1 models improves the DCM phenotype. Hence, the work demonstrates a potential mechanism for disease development and its amelioration.

Major comments:

1. As pericardial cells are probed and mentioned substantially in this paper, the authors should explain what these cells do in flies. While affiliated with the heart, they are not myocytes and are probably not particularly relevant to the human heart. In this regard, it is possible that the phenotypes observed in the heart are partially or completely the result of Hand-driven expression of transgenes in the pericardial cells. Although unlikely, this issue should be mentioned as well.

2. Does human miR-1 target Col15A1 transcripts based upon in silico analysis? This issue should be mentioned and discussed.

Minor comments:

1. The abstract should explicitly state that Multiplexin is a form of collagen.

2. More information on the identity between the Drosophila and human forms of miR-1 would be helpful to establish that they are conserved. What is the percent identity and are the sequences that target mRNAs homologous?

3. In Figure 1C, it appears that there is an increased heartbeat frequency and arrhythmicity. Are these mutant phenotypes as well?

4. Incomplete sentence (page 5): We identified a set of candidate genes, of which Multiplexin (Mp)

5. What is the basis for studying Multiplexin function as opposed to other candidates that were identified? It would be useful to mention this in the Results, although it is mentioned in the Discussion ("We top-ranked Mp because of its known role in setting the size of the cardiac lumen").

6. "Mp was detected on the luminal and external surfaces of the cardiomyocytes ensuring cardiac contractions" Why does this ensure cardiac contractions?

7. Need to state in the text that the increased levels of Col15A1 transcript expression in DM1 patients was not statistically significant.

8. Need a magnification bar for Figures 5F-H.

9. Please speculate as to why the third DM1 model does not recapitulate the cardiac phenotypes.

10. Did the confocal studies indicate whether there was myofibrillar disarray in the heart tubes?

11. For the statistical comparisons in the figures, please indicate in the legends that statistically significant differences ($p<0.05$) are shown.

12. Please more thoroughly explain the UPRT control line.

13. Figure S1 legend: "(red) and 5 (darck)"; the latter should read "(black)"

14. Figure S2 panels J and K: it would be helpful to indicate what is being measured on the Y axis, e.g., Mean intensity of dmiR-1 levels. This is true for the various panels in other figures labeled CTCF on their Y axes.

CROSS-CONSULTATION COMMENTS

All reviewers agree that this is a well-designed study and that the manuscript is well written. The missing Hand-Gal4 control mentioned by Reviewers 1 and 3 seems an important element that is missing. These reviewers also call into question the FISH quantification methodology. These two issues seem the most critical to resolve. The other additional experiments suggested deserve input from the authors as to whether they already have relevant data that can be cited, whether they are important to pursue or if they go beyond the scope of the current study. Reviewers 1 and 2 agree that further discussion of the fly model that does not show DCM should be provided. The question on fibrosis in the fly models is germane (Reviewer 3), although it might be indirectly addressed by the fact that a collagen molecule is upregulated here (a major player in fibrosis). All of the minor comments are reasonable and should be addressed by the authors.

2. Significance:

Significance (Required)

This paper is significant in that it draws a more direct connection between the reduction in a microRNA that occurs in myotonic dystrophy and dilated cardiomyopathy that is affiliated with this disease. It shows that a form of collagen that is overexpressed in both Drosophila models and humans with DM1-caused DCM is causative/correlated with the increased heart diameters. Thus, the fly model provides important insights into the link between the mutant gene and the cardiac phenotype. This work will be of interest to those studying skeletal and cardiac muscle disease and scientists interested in developing potential therapeutics for treating DM1-caused DCM. Note that my expertise is in producing and studying skeletal muscle and cardiac disease models in the Drosophila system, which is relevant to evaluating this paper and defining its significance in the field.

3. How much time do you estimate the authors will need to complete the suggested revisions:

Estimated time to Complete Revisions (Required)

(Decision Recommendation)

Less than 1 month

4. *Review Commons* values the work of reviewers and encourages them to get credit for their work. Select 'Yes' below to register your reviewing activity at [Web of Science Reviewer Recognition Service]() (formerly

Publons); note that the content of your review will not be visible on Web of Science.

Web of Science Reviewer Recognition

Yes

Review #3

1. Evidence, reproducibility and clarity:

Evidence, reproducibility and clarity (Required)

A current study demonstrates that miR-1 targets the newly identified heat-specific target Multiplexin, which, when upregulated, exhibits similar phenotypes observed for the Drosophila DM1 model. Furthermore, the authors additionally confirm some of their results using samples derived from DM1 patients and support the data obtained in flies. Overall, it is a good study with well-performed experiments. The data presented in the paper are convincing and most of the claims the authors provide are supported by their findings. Manuscript is clearly written and easy to read and understand. Statistical analysis and the description of the methods are appropriate. It is an interesting paper and I would highly support it to be accepted for publication; however, I few comments I would like authors to address.

Major comments:

1. The cardiac dilation/fractional shortening phenotype in Hand>dmiR-1-KD flies is only observed in young flies but not in old flies. However, heart-targeted Mp overexpression leads to DCM in aged flies. Could authors comment on this?

2. Since the HAND-Gal4 line was used to drive multiple transgenes, it would be important to have the cardiac dilation/fractional shortening phenotype measured in this line as a control.

3. The use of a sponge line is highly appreciated as it allows for tissue-specific downregulation of miRNA. However, to corroborate the data, I would recommend including the knockdown mutant that is available in Bloomington as additional confirmation since no qPCR is provided for the efficacy of the sponge line. This line could also be used in combination with reporter lines to perform a targeting experiment.

4. The authors state that the reduced miR-1 levels have already been shown in DM1 patients. It would be a stronger argument if similar downregulation was shown in

patient samples used in this manuscript (qPCR would be sufficient).

5. Because fibrosis is a hallmark of myotonic dystrophy, do the authors have some makers or other methods to test whether observed phenotypes are due to fibrosis?

6. The explanation of the observation that pre-miR-1 levels are down-regulated only in young flies, whereas old flies show an opposite tendency, is missing. The authors suggested that this is due to "impaired processing". To corroborate this interesting hypothesis, the authors performed only the smFISH intensity analyses, which are somewhat difficult to decipher. I would recommend, in addition to the pre-miRNA levels, to test and compare the mature miRNA expression using TaqMan qPCR.

7. The relationship between Bru3 and miR-1 shown in the schematic is not well-defined and would rather require a question mark or dotted line, as the authors provide no evidence that Bru3 can be directly involved in miR-1 processing. The authors suggest that CELF1 may bind UG-rich miRNAs and mediate their degradation by recruiting poly(A)-specific ribonuclease (PARN), but this is only a hypothesis and does not justify the placement of a direct line of repression on the schematic in the last figure.

8. I also feel that the authors did not clearly explain the cardiac phenotypes in terms of systolic and diastolic diameter measurements. Which parameters clearly represent the DM1 model, specifically higher or lower diameters of systole and diastole? Results should be clearly indicated in figure legends.

Minor comments:

1. The full name of CELF1 on page 2: CUGBP Elav-like family member 1 should be added.

2. For better readability of the text and corresponding figures, consistent use of UAS-Mp or UAS-3HNC1 is recommended, but not a mixture of both.

3. Why is the Multiplexin overexpression line called UAS-3HNC1?

4. For all figures, it would be better if the genotypes were indicated in the panels and the graphs had the age of the flies instead of color coding.

5. Figure 5. Were technical replicates performed for the western blot shown in 5B?

6. Figure S1-S2. Why the data for qPCR of miR-1 is in figure S1 and not S2?

7. Figure S4. Misspelling in figure legend: Scale "barre" instead of scale "bar".

2. Significance:

Significance (Required)

The manuscript "Deregulations of miR-1 and its target Multiplexin promote dilated cardiomyopathy associated with myotonic dystrophy type 1" by Souidi et al., reports a novel role of identified Multiplexin (Mp) as a new cardiac miR-1 target involved in

myotonic dystrophy type 1 (DM1) using Drosophila as a model system. Myotonic dystrophy type 1 (MD1) is a severe disease that results in a multisystem disorder affecting the skeletal and smooth muscles as well as the eye, heart, endocrine system, and central nervous system. At the moment, no appropriate treatment has been identified to prevent it. Previous studies have also shown that heart-specific miR-1 levels are reduced in patients with DM1, but the role and targets of this miRNA in the heart have not been analyzed. Research presented in this paper is of a broad interest and provide new evidence that will help to better understating DM1 on molecular level. It will be interesting not only to scientists from the Drosophila field but will also contribute to medical research field.

3. How much time do you estimate the authors will need to complete the suggested revisions:

Estimated time to Complete Revisions (Required)

(Decision Recommendation)

Between 1 and 3 months

4. *Review Commons* values the work of reviewers and encourages them to get credit for their work. Select 'Yes' below to register your reviewing activity at Web of Science Reviewer Recognition Service (formerly Publons); note that the content of your review will not be visible on Web of Science.

Web of Science Reviewer Recognition

Yes

Full Revision

Manuscript number: RC- 2022-01663
Corresponding author(s): Krzysztof Jagla

[Please use this template only if the submitted manuscript should be considered by the affiliate journal as a full revision in response to the points raised by the reviewers.

If you wish to submit a preliminary revision with a revision plan, please use our "Revision Plan" template. ***It is important to use the appropriate template to clearly inform the editors of your intentions.****]*

1. General Statements [optional]

We would like thank all reviewers for appreciating our work and for constructive comments.
To answer all the raised points we performed several additional experiments with data reported in new panels of main figures and in new supplemental figures. Revisions in the text are in red.
We hope that this fully revised version of our manuscript will be suitable for publication.

2. Point-by-point description of the revisions

Our answers are in italics

Reviewer #1 (Evidence, reproducibility and clarity (Required)):

The paper titled "Deregulations of miR-1 and its target Multiplexin promote dilated cardiomyopathy associated with myotonic dystrophy type 1" by the Jagla group studied the effect of down-regulation of miR-1 in myotonic dystrophy type 1 (DM1) using fly as the disease model. The study is based on previous findings that in DM1 MBNL1 is sequestered, CELF1 is stabilized, and miR-1 is down regulated. The authors further identified Multiplexin to be the target effector of miR-1 in the fly heart and studied its function with a series of gain- and loss-of-function and rescue experiments. The authors' findings represent a significant advance in understanding the genetic mechanisms that can explain the pathogenic causes of dilated cardiomyopathy associated with DM1. Overall, this paper is well written and organized, with well-designed experiments and a clear model. A few additional experiments are suggested to further strengthen the conclusion.

We are grateful to the Reviewer for appreciating quality and significance of our work.

Major points:

1. Wild-type and Hand-Gal4 controls are missing for all experiments (only UAS lines outcrossed

to w1118 are displayed). Also, Hand-Gal4 driver lines can cause mild dilation by itself, which would influence interpretation and statistics of data.

We agree and include the Hand-Gal4/+ control condition to all main and supplemental figures showing heart parameters. The differences in data statistics using Hand-Gal4/+ compared to UAS/+ control lines reinforce our data interpretation.
They are listed below:

- *i) increase in diastolic diameter and reduction of fractional shortening become statistically significant in Hand>miR-1sponge hearts at 5 weeks (Fig. 1D, F);*
- *ii) reductions of fractional shortening become significant in Hand>Bru3 (Fig. 2C) and Hand>mblRNAi (Fig. 2F) contexts at 1 week;*
- *iii) increase in diastolic diameter of Hand>mblRNAi hearts at 5 weaks becomes statistically significant (Fig. 2D);*
- *iv) increase in diastolic diameter of Hand>Mp hearts at 5 weeks (Fig. 4E) becomes statistically significant ;*
- *v) reduction of fractional shortening becomes statistically significant in Hand>Mp context at 1 week (Fig. 4G);*
- *vi) increase in diastolic diameter in Hand>960CTG context at 5 weeks becomes statistically significant (Fig. S2A).*

Also, the authors should consider to confirm the miR-1 phenotype obtained with the sponge with a miR mutant, and also combine miR-1 het with miR sponge (worsening of phenotype?). Alternatively, knockdown efficiency of miR should be tested by qPCR or HCR/smFISH.

We are grateful for these comments. Below we refer to published data and to performed additional experiments that are in support of miR-1 sponge phenotypes:

- *UAS-miR-1 sponge line we used was generated and tested by Fulga et al., (Nat Comm, 2015). Fulga and colleagues apply UAS-miR1sponge line to attenuate miR-1 function in muscles and obtain miR-KO-like muscle phenotypes.*
- *Here, we identify Mp as a new direct miR-1 target. To test whether miR-1 sponge attenuates miR-1 function we analyzed Mp protein levels in the hearts from wt and Hand>miR-1sponge flies. Mp expression is highly increased in Hand>miR-1sponge context indicating attenuation of miR-1 by the sponge transgene. These data are presented in new Fig. S5J;*
- *We tested whether heterozygous dmiR-1 KO -/+ flies (homozygous dmiR-1 mutants are lethal) develop Hand>miR-1sponge-like heart phenotype. Indeed, at 5 weeks of age dmiR-1 KO -/+ flies show significantly increased diastolic and systolic heart diameters. Thus, in old flies loss of one copy of miR-1 mimics heart dilation observed in Hand>dmiR-1sponge context. Heart contractility remains unaffected in dmiR-1 KO -/+ flies, suggesting that loss of one copy of miR-1 has a weaker impact on heart function than heart-targeted miR-1sponge. These data are shown in a new supplemental figure (Fig. S4A-C).*

2. It is surprising that one of the DM1 fly models, overexpression of 960 CTG repeats, did not show DCM, considering it is the primary cause of DM1 in humans due to excessive CTG repeats. It should be discussed why Hand>960 CTG does not lead to DCM, since the authors claim that this model with high number of CTG repeats shows a strong phenotype. Are Hand>bru3 and Hand>mbl stronger?

We thank the reviewer for pointing this out.
Heart and muscle-specific DM1 models we established and tested (Hand> or Mef>960CTG, Hand> or Mef>mblRNAi and Hand> or Mef>Bru3) all develop the majority of DM1 phenotypes (Picchio et al., 2013 ; Picchio et al., 2018 ; Auxerre-Planté et al., 2019). However, some cardiac DM1 phenotypes such as conduction defects (Auxerre-Plantié et al., 2019) and described here DCM are only observed in Hand>Bru3 and Hand>mblRNAi contexts. We previously observed that the down-regulation of sarcomeric genes is more important in Mef>Bru3 than in Mef>960CTG context (Picchio et al., 2018). This could result from a milder effect of 960CTG repeats on Bru3 and Mbl levels when compared with Gal4-driven overexpression of Bru3 and RNAi-knockdown of mbl. We add a comment to Results section (page 5) to discuss this point: "The Hand>960CTG line shows cardiac dilation at 5 weeks of age characterized by significant increase in diastolic and systolic diameters but with normal cardiac contractility (Fig. S2A,B,C). We hypothesise that non-affected contractility in this DM1 line is due to a milder effect of 960CTG repeats on Bru3 and Mbl levels compared to GAL4-driven overexpression of Bru3 and RNAi-knockdown of mbl."

Is miR-1 (and Mp) unaltered in these flies with 960 CTG repeats?

In Hand>960CTG context a reduced level of miR-1 and an increase in Mp are also observed (not shown). Hand>960CTG flies do not develop DCM but at 5 weeks of age show a significant increase in diastolic and systolic heart diameters. One possibility we favor is that deregulation of miR-1 and Mp in Hand>960CTG is under the level that induces DCM. By analogy, only DM1 patients with a high increase in Col15A1 develop DCM (Fig. 5).

It would be interesting to overexpress 960 CTG in a miR-1 or mbl heterozygous mutant background, which may produce DCM.

To test additional genetic context in which miR-1 is reduced we used miR-1 heterozygous KO flies. We found that in 5 weeks-old flies loss of one copy of miR-1 leads, like in Hand>miR-1sponge flies, to heart dilation (Fig. S4A-C), however mir-1KO +/- flies do not show affected contractility.
We agree that combining Hand>960CTG with mir-1 heterozygous mutants would potentially result in an additional DCM producing context. As we are focusing on our DCM developing DM1

models (Hand>Bru3 and Hand>mblRNAi) and on conserved deregulation of miR-1 and its target Mp/Col15A1, we didn't follow this suggestion.

3. In Figure 2H, the mean intensity is displayed as the readout of the smFISH quantification of miR-1 levels. If understood correctly, this is the wrong readout since smFISH detects single molecule fluorescence of transcripts, so the number of transcripts should be quantified.

The miR-1 quantification was done using FISH with miR-1-specific LNA probe (Qiagen miRCURY system). This is highly sensitive ISH but the resolution is not at a single molecule level. The Imaris-generated spots in Fig. 2G and 2G' represent (for each of them) several miR-1 molecules. The mean intensity of the fluorescent signal for a given spot is proportional to the number of miR-1 molecules and the average of the mean intensities of all spots illustrates the level of miR-1 expression (Fig. 2H). We remove "sm" abbreviations from the text and Figure legend and provide a more detailed description of miR-1 quantification in the Method section.

Furthermore, Hand-Gal4 is not expressed in the ventral longitudinal muscles (VLM). As a proof of principle, miR-1 levels should be quantified in VLM (no change in transcripts levels expected).

When cross with UAS-GFP the Hand-Gal4 expression could be detected in VLMs even if VLM associated GFP signal is much lower than in cardioblasts (CB) and pericardial cells (PC). Representative views of Hand>GFP hearts labeled with anti-GFP are shown below.

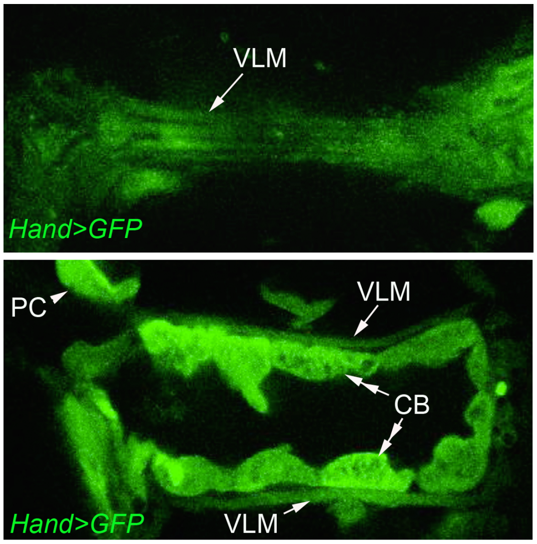

4. Fig. 5: Since DCM- DM1 patients still show elevated COL15A1 levels but no DCM, it would be interesting to know if DCM phenotypes are COL15A1-dosage dependent. This could be easily tested in the fly model by testing UAS-Mp overexpression at different temperatures.

Heart parameters are to some extent temperature-sensitive (our observations) thus in our view increasing targeted Mp expression by elevating temperature is not appropriate for heart physiology experiments.
Presented in the manuscript data on Mp overexpression at 25°C already provide some indication for Mp dose-dependent effect in the fly model. We observe that DCM is induced at both 1 and 5 weeks of age, but the cardiac tube dilation is less important in 1 than in 5 weeks-old flies (Fig. 4). Also, when analysing Hand>960CTG DM1 model we observed that young flies with a low Mp levels do not show cardiac dilation while aged Hand>960CTG flies display an increase in diastolic and systolic heart diameters concomitant with a higher Mp.

5. The authors elegantly show rescue of Hand>Bru3 flies by Mp RNAi. Their model would be further strengthened if a similar rescue can be shown with Hand>mblRNAi.

So far we were unsuccessful in generation of recombined mblRNAi ;MpRNAi line most probably because of incompatibility in chromosomal transgene locations. Thus, we were unable to perform this experiment.
Because gene deregulations and DCM phenotypes we describe are highly similar in Hand>Bru3 and Hand>mblRNAi context we believe that rescue experiment we provide is representative for both DM1-associated DCM contexts.

Minor points:

Fig. 1A,B: Ventral longitudinal muscles are covering the hearts on these images, so it's difficult to see the heart dimensions. This holds true for images throughout the manuscript. Where were the diameters measured (by the valves)? A better description and illustration would help the reader understand the situation.

In the lateral heart views as in Fig. 1A and B it is indeed difficult to appreciate heart dimensions. For this reason we always show transversal sections derived from 3D reconstruction (as in Fig. 1A' and B'). In this context differences in the internal heart diameters could be appreciated (white lines). All diastolic and systolic heart diameter measures presented in the graphs are extracted from the SOHA registrations (see Methods).

Fig. 1 A',B': White line does not reflect the location where SOHA data are measured and should be horizontal for consistency. Where is ventral vs. dorsal?

We agree. We indicate where is ventral and dorsa. For consistency we remove white lines from panels 1A and 1B and maintain orientations of white lines in panels 1A' and 1B'.

Fig. 1D-F: Annotate 1 and 5 weeks in Figure, please. Also, why were 1 and 5 weeks tested? Is there an age-component in DM1 phenotype severity?

We add 1 and 5 weeks indications to the figures and discuss in the text (Results section page 4) that 1 and 5 weeks analyses were applied because the severity of cardiac phenotypes increases with age.

Fig. 3A: Transcriptional analysis was done at which stage of development?

It was done at 5 weeks of age. We add information to the figure legend.

Fig. 3: It is not clear, in which set the authors looked for miR-1 bindings sites (144 genes or the whole set)? Not well annotated. What is meant by 'heart-targeted'?

In silico search was performed on the whole set of genes. We provide more precisions on in silico screen in Method section.

Fig. 4C,D: It looks like they are not shown in the same dorsal-ventral orientation. Also, it looks Mp is overexpressed in the VLM, but Hand-Gal4 only drives in the cardiomyocytes and pericardial cells? How was quantification done?

We are thanking for pointing this out. We revised heart orientations in panel 4C and 4D. As previously mentioned Hand-Gal4 is also expressed in the VLM. We present a more representative view in 4D with a lower Mp signal in VLMs. Quantification of Mp expression is not presented here but performed like in Fig. 3G.

Fig. 4I: Why are some myofibers indicated in red in the model?

In red are indicated additional actin filaments that form in the case of heart dilation. As we do not discuss this aspect we modify drawing in the model.

Fig. 5 D-E: Genotypes need to be better indicated in the graphs.

We provide now more complete genotypes.

Did the authors control for multiple UAS sites? Is UPRT a UAS control?
Yes, indeed UPRT is the UAS line.

In the first paragraph of Result 3, the last sentence seems unfinished. "We identified a set of candidate genes, of which Multiplexin (Mp)"
We revise this sentence.

In Method, the in silico screening for miR-1 target should be explained in more detail.
We provide a more detailed in silico screening protocol in Method section.

Reviewer #1 (Significance (Required)):

The presented data is a significant advance our knowledge of our understanding of the molecular mechanisms involved in DM1. I expect that scientists in the muscular disease field and beyond will find this work of high interest.

Reviewer #2 (Evidence, reproducibility and clarity (Required)):

Summary:
This well-written manuscript utilizes the Drosophila model system to demonstrate that reduction of micro-RNA miR-1 and the resulting increase in one of its down-regulated proteins (Multiplexin) contribute to a dilated cardiomyopathy (DCM) phenotype. This is of interest in that this particular micro-RNA is downregulated in myotonic dystrophy type 1 (DM1), and this correlates with the DCM phenotype observed in patients. Further, the authors show that the human ortholog of Multiplexin is enriched in human DM1-DCM hearts and that downregulation of this protein in Drosophila DM1 models improves the DCM phenotype. Hence, the work demonstrates a potential mechanism for disease development and its amelioration.

We are grateful to the Reviewer for appreciating our work and pointing out potential impact of our findings.

Major comments:
1. As pericardial cells are probed and mentioned substantially in this paper, the authors should explain what these cells do in flies. While affiliated with the heart, they are not myocytes and are probably not particularly relevant to the human heart. In this regard, it is possible that the phenotypes observed in the heart are partially or completely the result of Hand-driven expression of transgenes in the pericardial cells. Although unlikely, this issue should be mentioned as well.

Hand-Gal4 driver is the most commonly used Drosophila cardiac driver. Regarding influence of Hand-Gal4 driven expression in pericardial cells on the heart phenotypes, we previously tested all our DM1 models using cardioblast-specific Tin-GAL4 driver. All cardiac phenotypes including DCM are also observed when using Tin-Gal4 driver (as shown for conduction defects in Fig.5B Auxerre-Plantié et al., Elife 2019) indicating that the phenotypes are mainly due to gene deregulations within the cardioblasts.

2. Does human miR-1 target Col15A1 transcripts based upon in silico analysis? This issue should be mentioned and discussed.

In silico analysis (new supplemental Fig.S4G) reveals that Col15A1 transcripts carry a perfect miR-1 seed site in 3'UTR region.

Minor comments:

1. The abstract should explicitly state that Multiplexin is a form of collagen.

We mention this in the abstract.

2. More information on the identity between the Drosophila and human forms of miR-1 would be helpful to establish that they are conserved. What is the percent identity and are the sequences that target mRNAs homologous?

Mature Drosophila and human miR-1 are highly homologous. We provide their sequences in new supplemental Fig. S4F.

3. In Figure 1C, it appears that there is an increased heartbeat frequency and arrhythmicity. Are these mutant phenotypes as well?

We check it again and do not observe any significant change in heart period or in arrhythmia index in Hand>miR1 sponge context in both young and old flies. We show a new more representative view of M-modes in panel 1C.

4. Incomplete sentence (page 5): We identified a set of candidate genes, of which Multiplexin (Mp)

This sentence was revised.

5. What is the basis for studying Multiplexin function as opposed to other candidates that were identified? It would be useful to mention this in the Results, although it is mentioned in the Discussion ("We top-ranked Mp because of its known role in setting the size of the cardiac lumen").

We add following sentences to Results section to clarify this point earlier in the manuscript.
"Mp overexpression in the developing embryonic heart leads to an enlargement of heart lumen and is sufficient to promote an increase of the embryonic aorta diameter to that of the heart proper (Harpaz et al., 2013). We thus reasoned that Mp could be involved in DM1-associated DCM."

6. "Mp was detected on the luminal and external surfaces of the cardiomyocytes ensuring cardiac contractions" Why does this ensure cardiac contractions?

We are grateful for pointing this out. Mp is not ensuring but could influence cardiac contractions. We revise this sentence by deleting its second part "ensuring cardiac contractions".

7. Need to state in the text that the increased levels of Col15A1 transcript expression in DM1 patients was not statistically significant.

We state this in the text.

8. Need a magnification bar for Figures 5F-H.

Scale bar is added.

9. Please speculate as to why the third DM1 model does not recapitulate the cardiac phenotypes.

Heart and muscle-specific DM1 models we established and tested (Hand> or Mef>960CTG, Hand> or Mef>mblRNAi and Hand> or Mef>Bru3) all develop the majority of DM1 phenotypes (Picchio et al., 2013 ; Picchio et al., 2018 ; Auxerre-Planté et al., 2019). However, some cardiac DM1 phenotypes such as conduction defects (Auxerre-Plantié et al., 2019) and described here DCM are only observed in Hand>Bru3 and Hand>mblRNAi contexts. We previously observed that downregulation of sarcomeric genes is higher in Mef>Bru3 than in Mef>960CTG contexts (Picchio et al., 2018). This could result from a milder effect of 960CTG repeats on Bru3 and Mbl levels when compared with Gal4-driven overexpression of Bru3 and RNAi-knockdown of mbl. We add a comment in Results section (page 5) to discuss this point: "The Hand>960CTG line shows cardiac dilation at 5 weeks of age characterized by significant increase in diastolic and systolic diameters but with normal cardiac contractility (Fig. S2A,B,C). We hypothesise that non-affected contractility in this DM1 line is due to a milder effect of 960CTG repeats on Bru3 and Mbl levels compared to GAL4-driven overexpression of Bru3 and RNAi-knockdown of mbl."

10. Did the confocal studies indicate whether there was myofibrillar disarray in the heart tubes?

Thank you for this comment. Yes, we observe myofibrillar disarray. We show disarray phenotypes in all DCM developing contexts in a new supplementary figure (Fig.S1).

11. For the statistical comparisons in the figures, please indicate in the legends that statistically significant differences ($p<0.05$) are shown.
We provide this precision in Figure legends.

12. Please more thoroughly explain the UPRT control line.

We provide information about UPRT line in the Results section (p.8).

13. Figure S1 legend: "(red) and 5 (darck)"; the latter should read "(black)"

Revised.

14. Figure S2 panels J and K: it would be helpful to indicate what is being measured on the Y axis, e.g., Mean intensity of dmiR-1 levels. This is true for the various panels in other figures labeled CTCF on their Y axes.

Revised as suggested by the Reviewer.

CROSS-CONSULTATION COMMENTS
All reviewers agree that this is a well-designed study and that the manuscript is well written. The missing Hand-Gal4 control mentioned by Reviewers 1 and 3 seems an important element that is missing. These reviewers also call into question the FISH quantification methodology. These two issues seem the most critical to resolve. The other additional experiments suggested deserve input from the authors as to whether they already have relevant data that can be cited, whether they are important to pursue or if they go beyond the scope of the current study. Reviewers 1 and 2 agree that further discussion of the fly model that does not show DCM should be provided. The question on fibrosis in the fly models is germane (Reviewer 3), although it might be indirectly addressed by the fact that a collagen molecule is upregulated here (a major player in fibrosis). All of the minor comments are reasonable and should be addressed by the authors.

We provide answer to these comments

Reviewer #2 (Significance (Required)):

This paper is significant in that it draws a more direct connection between the reduction in a microRNA that occurs in myotonic dystrophy and dilated cardiomyopathy that is affiliated with this disease. It shows that a form of collagen that is overexpressed in both Drosophila models and humans with DM1-caused DCM is causative/correlated with the increased heart diameters. Thus, the fly model provides important insights into the link between the mutant gene and the cardiac phenotype. This work will be of interest to those studying skeletal and cardiac muscle

disease and scientists interested in developing potential therapeutics for treating DM1-caused DCM. Note that my expertise is in producing and studying skeletal muscle and cardiac disease models in the Drosophila system, which is relevant to evaluating this paper and defining its significance in the field.

Reviewer #3 (Evidence, reproducibility and clarity (Required)):

A current study demonstrates that miR-1 targets the newly identified heat-specific target Multiplexin, which, when upregulated, exhibits similar phenotypes observed for the Drosophila DM1 model. Furthermore, the authors additionally confirm some of their results using samples derived from DM1 patients and support the data obtained in flies. Overall, it is a good study with well-performed experiments. The data presented in the paper are convincing and most of the claims the authors provide are supported by their findings. Manuscript is clearly written and easy to read and understand. Statistical analysis and the description of the methods are appropriate. It is an interesting paper and I would highly support it to be accepted for publication; however, I few comments I would like authors to address.

We are grateful to the Reviewer for his enthusiastic and supportive comments on our work.

Major comments:

1. The cardiac dilation/fractional shortening phenotype in Hand>dmiR-1-KD flies is only observed in young flies but not in old flies. However, heart-targeted Mp overexpression leads to DCM in aged flies. Could authors comment on this?

We speculate that attenuation of miR-1 in the heart could lead to more drastic pro-DCM alterations thus leading to earlier phenotypes than in the case of Mp overexpression.
To better assess DCM phenotypes we included additional Hand-Gal4/+ control context and more heart samples from 5 weeks-old flies. These additional analyses presented in revised Fig. 1D-F reveal that old Hand>dmiR-1KD flies, like the young one, also develop DCM phenotype.

2. Since the HAND-Gal4 line was used to drive multiple transgenes, it would be important to have the cardiac dilation/fractional shortening phenotype measured in this line as a control.

We performed this control experiments and suggested by the reviewer they are now included to the graphs.

3. The use of a sponge line is highly appreciated as it allows for tissue-specific downregulation of miRNA. However, to corroborate the data, I would recommend including the knockdown mutant that is available in Bloomington as additional confirmation since no qPCR is provided for the efficacy of the sponge line. This line could also be used in combination with reporter lines to perform a targeting experiment.

We are grateful for these comments. Below we refer to performed additional experiments:

- *We tested whether heterozygous dmiR-1 KO -/+ flies (homozygous dmiR-1 mutants are lethal) develop Hand>miR-1sponge-like heart phenotype. Indeed, at 5 weeks of age dmiR-1 KO -/+ flies show significantly increased diastolic and systolic heart diameters. Thus, in old flies loss of one copy of miR-1 mimics heart dilation observed in Hand>dmiR-1sponge context. Heart contractility remains unaffected in dmiR-1 KO -/+ flies, suggesting that loss of one copy of miR-1 has a weaker impact on heart function than heart-targeted miR-1sponge. These data are shown in a new supplemental figure (Fig. S4A-C).*
- *Here, we identify Mp as a new direct miR-1 target. To test whether miR-1 sponge attenuates miR-1 function we analyzed Mp protein levels in the hearts from wt and Hand>miR-1sponge flies. Mp expression is highly increased in Hand>miR-1sponge context indicating attenuation of miR-1 by the sponge transgene. These data are presented in new Fig. S5J;*

4. The authors state that the reduced miR-1 levels have already been shown in DM1 patients. It would be a stronger argument if similar downregulation was shown in patient samples used in this manuscript (qPCR would be sufficient).

We performed suggested by the reviewer analyses of miR-1 in patient samples. We show that miR-1 is indeed down regulated. These new data supporting conserved pro-DCM deregulation of miR-1 and its target Mp/Col15A1 are shown in new Fig 5C.

5. Because fibrosis is a hallmark of myotonic dystrophy, do the authors have some makers or other methods to test whether observed phenotypes are due to fibrosis?

Fibrosis (replacement of muscle by fibrotic tissue) has not been reported in Drosophila and is not associated with degeneration of body wall or cardiac Drosophila muscles in so far described fly models of human muscular dystrophies. However, one could speculate that increase in Mp/Col15A1 levels within the ECM of diseased DM1 cardiac cells we observe, could have, a fibrotic-like, negative effect on cardiac function.

6. The explanation of the observation that pre-miR-1 levels are down-regulated only in young flies, whereas old flies show an opposite tendency, is missing.

Accumulation of pre-miR-1 in old flies is most probably due to the affected processing mediated by mbl. This is correlated with the reduction of the mature miR-1.

The authors suggested that this is due to "impaired processing". To corroborate this interesting hypothesis, the authors performed only the smFISH intensity analyses, which are somewhat

difficult to decipher. I would recommend, in addition to the pre-miRNA levels, to test and compare the mature miRNA expression using TaqMan qPCR.

Impaired miR-1 processing is supported by the previous studies in human cells (Rau et al., 2011) and in Drosophila models (Fernandez-Costa et al., 2013). We believe that our method of quantification of miR-1 expression via highly sensitive miRCURY LNA FISH is a well-adapted method. It was performed with all necessary controls. In the method section we provide now more details for the LNA FISH based miR-1 quantification approach.

In parallel, TaqManPCR-based miR-1 quantification was performed for human cardiac samples from DM1 patients.

7. The relationship between Bru3 and miR-1 shown in the schematic is not well-defined and would rather require a question mark or dotted line, as the authors provide no evidence that Bru3 can be directly involved in miR-1 processing. The authors suggest that CELF1 may bind UG-rich miRNAs and mediate their degradation by recruiting poly(A)-specific ribonuclease (PARN), but this is only a hypothesis and does not justify the placement of a direct line of repression on the schematic in the last figure.

We agree and modify scheme accordingly.

8. I also feel that the authors did not clearly explain the cardiac phenotypes in terms of systolic and diastolic diameter measurements. Which parameters clearly represent the DM1 model, specifically higher or lower diameters of systole and diastole? Results should be clearly indicated in figure legends.

We provide appropriate precisions in figure legends.

Minor comments:

1. The full name of CELF1 on page 2: CUGBP Elav-like family member 1 should be added.

Revised

2. For better readability of the text and corresponding figures, consistent use of UAS-Mp or UAS-3HNC1 is recommended, but not a mixture of both.

We consistently use UAS-Mp in the revised version

3. Why is the Multiplexin overexpression line called UAS-3HNC1?

This is the name that resumes protein Mp domains: Collagen tripple helix and trimerization region (3H) and NC1 domain (C-terminal non-triple helical domain) comprising Endostatin domain. We provide this information in Methods section.

4. For all figures, it would be better if the genotypes were indicated in the panels and the graphs had the age of the flies instead of color coding.

We revised these points as suggested.

5. Figure 5. Were technical replicates performed for the western blot shown in 5B?

We didn't perform technical replicates because of limited human sample amounts

6. Figure S1-S2. Why the data for qPCR of miR-1 is in figure S1 and not S2?

In the revised version all supplemental analyses on miR1 are included to the new Fig. S4

7. Figure S4. Misspelling in figure legend: Scale "barre" instead of scale "bar".

Revised

Reviewer #3 (Significance (Required)):

The manuscript "Deregulations of miR-1 and its target Multiplexin promote dilated cardiomyopathy associated with myotonic dystrophy type 1" by Souidi et al., reports a novel role of identified Multiplexin (Mp) as a new cardiac miR-1 target involved in myotonic dystrophy type 1 (DM1) using Drosophila as a model system. Myotonic dystrophy type 1 (MD1) is a severe disease that results in a multisystem disorder affecting the skeletal and smooth muscles as well as the eye, heart, endocrine system, and central nervous system. At the moment, no appropriate treatment has been identified to prevent it. Previous studies have also shown that heart-specific miR-1 levels are reduced in patients with DM1, but the role and targets of this miRNA in the heart have not been analyzed. Research presented in this paper is of a broad interest and provide new evidence that will help to better understating DM1 on molecular level. It will be interesting not only to scientists from the Drosophila field but will also contribute to medical research field.

Dear Dr. Jagla,

Thank you for the submission of your revised manuscript. We have now received the enclosed reports from the referees. Referee 1 still has minor suggestions that I would like you to incorporate before we can proceed with the official acceptance of your manuscript.

A few editorial requests will also need to be addressed:

- You manuscript contains 5 main figures but is layed out as a full article. Please either add one more main figure or combine the results and discussion sections to publish your study as a short report (with a maximum of 27.000 characters). You can find more info in our guide to authors online.

- Please add up to 5 keywords to the ms file.

- Please add a Data Availability Section (DAS) to the end of the materials and methods. If you have not deposited data in public databases please mention this fact in the DAS.

- Please add a "Disclosure and Competing Interest" (former conflict of interest) statement to the ms file.

- The reference format needs to be corrected to EMBO reports (Harvard) style. Only the first 10 authors should be listed, followed by et al. The DOIs need to be removed.

- Please fill in and send us a completed author checklist that can be found here: <https://www.embopress.org/page/journal/14693178/authorguide> The completed checklist will also be part of the transparent peer review process file (RPF).

- The funding info in our online ms system and in the ms itself dont match. Please correct.

- Please upload all main and all EV figures as individual files.

- Fig 4A,H; 5G,H,I, S1A,D; S3A-K; S4A-C,F; S5A,C; S6A-F; S7A-C callouts are missing, please add.

- Your ms has 7 supplementary figures and 1 table. We offer a maximum of 5 expanded view (EV) figures that appear inline in the ms html version. If you move 1 suppl figure to the main ms you could may be combine the 6 remaining suppl figures into 5 EV figures. The legends for the EV figures need to be moved to after the main figure legends. You can find more info on our format requirements online in our guide to authors. The table could be a table in the main ms file or could be called Table EV1, which needs to have a title in the table file. The EV table can be an excel file, which might help to display the data.

- In Figure 3K there seem to be small segments missing (top corners) from the scans in the left figure. Please send us the source data for panel 3K.

- I attach to this email a related ms file with comments by our data editors. Please address all comments in the final ms.

EMBO press papers are accompanied online by A) a short (1-2 sentences) summary of the findings and their significance, B) 2-3 bullet points highlighting key results and C) a synopsis image that is exactly 550 pixels wide and 200-600 pixels high (the height is variable). You can either show a model or key data in the synopsis image. Please note that text needs to be readable at the final size. Please send us this information along with the revised manuscript.

I would like to suggest some changes to the abstract that needs to be written in present tense. Please let me know whether you agree with the following:

Myotonic dystrophy type 1 (DM1) is the most common muscular dystrophy in adults. It is caused by the excessive expansion of non-coding CTG repeats, which when transcribed affect functions of RNA-binding factors with adverse effects on alternative splicing, processing and stability of a large set of muscular and cardiac transcripts. Among these effects, inefficient processing and down-regulation of muscle- and heart-specific miRNA, miR-1, has been reported in DM1 patients, but the impact of reduced miR-1 on DM1 pathogenesis has been unknown. Here, we use Drosophila DM1 models to explore the role of miR-1 in cardiac dysfunction in DM1. We show that miR-1 downregulation in the heart leads to dilated cardiomyopathy (DCM), a DM1-associated phenotype. We combined in silico screening for miR-1 targets with transcriptional profiling of DM1 cardiac cells to identify miR-1 target genes with potential roles in DCM. We identify Multiplexin (Mp) as a new cardiac miR-1 target involved in DM1. Mp encodes a collagen protein involved in cardiac tube formation in Drosophila. Mp and its human ortholog Col15A1 are both highly enriched in cardiac cells of DCM-developing DM1 flies and in heart samples from DM1 patients with DCM, respectively.

Importantly, when overexpressed in the heart, Mp induces DCM, whereas its attenuation rescues the DCM phenotype of aged DM1 flies. Reduced levels of miR-1 and consecutive up-regulation of its target Mp/Col15A1 are thus critical in DM1-associated DCM.

I look forward to seeing a final version of your manuscript as soon as possible. Please use this link to submit your revision: https://embor.msubmit.net/cgi-bin/main.plex

Best regards,
Esther

Esther Schnapp, PhD
Senior Editor
EMBO reports

Referee #1:

The authors have done an excellent job at improving the paper and have generally dealt with the issues I raised. I have two comments that I believe would be helpful to consider:

1. Although the authors adequately explain the lack of relevance of pericardial cells to the current work in their reviewers response, this information does not appear to be added to the manuscript. I still think the reader would benefit from an explanation of what a pericardial cell is and an indication that the previous use of the Tin-Gal4 driver obviated the possibility that they are implicated in the heart phenotypes described.

2. Page 8: "and the cardiac contractility is ameliorated" should read "and the cardiac contractility defect is ameliorated"

Referee #2:

The authors have made all required changes and performed the requested additional experiments and in my opinion, the manuscript is acceptable for publication in the EMBO reports journal.

Referee #3:

The revised manuscript is recommended for publication in its current revised form. The author responded adequately to the reviewers' criticism.

The authors have addressed all editorial requests.’

Dr. Krzysztof Jagla
GReD, INSERM, CNRS, University of Clermont Auvergne
28 Place Henri Dunant
Auvergne 63270
France

Dear Dr. Jagla,

I am very pleased to accept your manuscript for publication in the next available issue of EMBO reports. Thank you for your contribution to our journal.

At the end of this email I include important information about how to proceed. Please ensure that you take the time to read the information and complete and return the necessary forms to allow us to publish your manuscript as quickly as possible.

As part of the EMBO publication's Transparent Editorial Process, EMBO reports publishes online a Review Process File to accompany accepted manuscripts. As you are aware, this File will be published in conjunction with your paper and will include the referee reports, your point-by-point response and all pertinent correspondence relating to the manuscript.

If you do NOT want this File to be published, please inform the editorial office within 2 days, if you have not done so already, otherwise the File will be published by default [contact: emboreports@embo.org]. If you do opt out, the Review Process File link will point to the following statement: "No Review Process File is available with this article, as the authors have chosen not to make the review process public in this case." Please note that the author checklist will still be published even if you opt out of the transparent process.

Thank you again for your contribution to EMBO reports and congratulations on a successful publication. Please consider us again in the future for your most exciting work.

Yours sincerely,

Esther Schnapp, PhD
Senior Editor
EMBO reports

****

THINGS TO DO NOW:

Once your article has been received by Wiley for production, the corresponding author will receive an email from Wiley's Author Services system which will ask them to log in and will present them with the appropriate license for completion.

You will receive proofs by e-mail approximately 2-3 weeks after all relevant files have been sent to our Production Office; you should return your corrections within 2 days of receiving the proofs.

Please inform us if there is likely to be any difficulty in reaching you at the above address at that time. Failure to meet our deadlines may result in a delay of publication, or publication without your corrections.

All further communications concerning your paper should quote reference number EMBOR-2022-56616V2 and be addressed to emboreports@wiley.com.

Should you be planning a Press Release on your article, please get in contact with emboreports@wiley.com as early as possible, in order to coordinate publication and release dates.

EMBO Press Author Checklist

Corresponding Author Name: Krzysztof Jagla	
Journal Submitted to: EMBO Reports	
Manuscript Number: EMBOR-2022-56616V1	

USEFUL LINKS FOR COMPLETING THIS FORM
The EMBO Journal - Author Guidelines
EMBO Reports - Author Guidelines
Molecular Systems Biology - Author Guidelines
EMBO Molecular Medicine - Author Guidelines

Reporting Checklist for Life Science Articles (updated January 2022)
This checklist is adapted from Materials Design Analysis Reporting (MDAR) Checklist for Authors. MDAR establishes a minimum set of requirements in transparent reporting in the life sciences (see Statement of Task: 10.31222/osf.io/9sm4x). Please follow the journal's guidelines in preparing your manuscript.
Please note that a copy of this checklist will be published alongside your article.

Abridged guidelines for figures
1. Data
The data shown in figures should satisfy the following conditions:
- the data were obtained and processed according to the field's best practice and are presented to reflect the results of the experiments in an accurate and unbiased manner.
- ideally, figure panels should include only measurements that are directly comparable to each other and obtained with the same assay.
- plots include clearly labeled error bars for independent experiments and sample sizes. Unless justified, error bars should not be shown for technical replicates.
- if n<5, the individual data points from each experiment should be plotted. Any statistical test employed should be justified.
- Source Data should be included to report the data underlying figures according to the guidelines set out in the authorship guidelines on Data Presentation.

2. Captions
Each figure caption should contain the following information, for each panel where they are relevant:
- a specification of the experimental system investigated (eg cell line, species name).
- the assay(s) and method(s) used to carry out the reported observations and measurements.
- an explicit mention of the biological and chemical entity(ies) that are being measured.
- an explicit mention of the biological and chemical entity(ies) that are altered/varied/perturbed in a controlled manner.
- the exact sample size (n) for each experimental group/condition, given as a number, not a range;
- a description of the sample collection allowing the reader to understand whether the samples represent technical or biological replicates (including how many animals, litters, cultures, etc.).
- a statement of how many times the experiment shown was independently replicated in the laboratory.
- definitions of statistical methods and measures:
  - common tests, such as t-test (please specify whether paired vs. unpaired), simple χ2 tests, Wilcoxon and Mann-Whitney tests, can be unambiguously identified by name only, but more complex techniques should be described in the methods section;
  - are tests one-sided or two-sided?
  - are there adjustments for multiple comparisons?
  - exact statistical test results, e.g., P values = x but not P values < x;
  - definition of 'center values' as median or average;
  - definition of error bars as s.d. or s.e.m.

Please complete ALL of the questions below.
Select "Not Applicable" only when the requested information is not relevant for your study.

Materials

Newly Created Materials	Information included in the manuscript?	In which section is the information available? (Reagents and Tools Table, Materials and Methods, Figures, Data Availability Section)
New materials and reagents need to be available; do any restrictions apply?	Yes	Materials and Methods

Antibodies	Information included in the manuscript?	In which section is the information available? (Reagents and Tools Table, Materials and Methods, Figures, Data Availability Section)
For **antibodies** provide the following information:<br>- Commercial antibodies: RRID (if possible) or supplier name, catalogue number and or/clone number<br>- Non-commercial: RRID or citation	Yes	Materials and Methods

DNA and RNA sequences	Information included in the manuscript?	In which section is the information available? (Reagents and Tools Table, Materials and Methods, Figures, Data Availability Section)
**Short novel DNA or RNA including primers, probes**: provide the sequences.	Yes	Materials and Methods

Cell materials	Information included in the manuscript?	In which section is the information available? (Reagents and Tools Table, Materials and Methods, Figures, Data Availability Section)
**Cell lines:** Provide species information, strain. Provide accession number in repository **OR** supplier name, catalog number, clone number, and/**OR** RRID.	Not Applicable	
**Primary cultures:** Provide species, strain, sex of origin, genetic modification status.	Not Applicable	
Report if the cell lines were recently **authenticated** (e.g., by STR profiling) and tested for mycoplasma contamination.	Not Applicable	

Experimental animals	Information included in the manuscript?	In which section is the information available? (Reagents and Tools Table, Materials and Methods, Figures, Data Availability Section)
**Laboratory animals or Model organisms:** Provide species, strain, sex, age, genetic modification status. Provide accession number in repository **OR** supplier name, catalog number, clone number, **OR** RRID.	Yes	Materials and Methods
**Animal observed in or captured from the field:** Provide species, sex, and age where possible.	Not Applicable	
Please detail **housing and husbandry conditions**.	Yes	Materials and Methods

Plants and microbes	Information included in the manuscript?	In which section is the information available? (Reagents and Tools Table, Materials and Methods, Figures, Data Availability Section)
**Plants:** provide species and strain, ecotype and cultivar where relevant, unique accession number if available, and source (including location for collected wild specimens).	Not Applicable	
**Microbes:** provide species and strain, unique accession number if available, and source.	Not Applicable	

Human research participants	Information included in the manuscript?	In which section is the information available? (Reagents and Tools Table, Materials and Methods, Figures, Data Availability Section)
If collected and within the bounds of privacy constraints report on age, sex and gender or ethnicity for all study participants.	Not Applicable	

Core facilities	Information included in the manuscript?	In which section is the information available? (Reagents and Tools Table, Materials and Methods, Figures, Data Availability Section)
If your work benefited from core facilities, was their service mentioned in the acknowledgments section?	Yes	Materials and Methods

Design

Study protocol	Information included in the manuscript?	In which section is the information available? (Reagents and Tools Table, Materials and Methods, Figures, Data Availability Section)
If study protocol has been **pre-registered, provide DOI in the manuscript**. For clinical trials, provide the trial registration number **OR** cite DOI.	Not Applicable	
Report the **clinical trial registration number** (at ClinicalTrials.gov or equivalent), where applicable.	Not Applicable	

Laboratory protocol	Information included in the manuscript?	In which section is the information available? (Reagents and Tools Table, Materials and Methods, Figures, Data Availability Section)
Provide DOI OR other citation details if **external detailed step-by-step protocols** are available.	Not Applicable	

Experimental study design and statistics	Information included in the manuscript?	In which section is the information available? (Reagents and Tools Table, Materials and Methods, Figures, Data Availability Section)
Include a statement about **sample size** estimate even if no statistical methods were used.	Yes	Materials and Methods and Figure legends
Were any steps taken to minimize the effects of subjective bias when allocating animals/samples to treatment (e.g. **randomization procedure**)? If yes, have they been described?	Not Applicable	
Include a statement about **blinding** even if no blinding was done.	Yes	We will include statement that no blinding was done to the final version
Describe **inclusion/exclusion criteria** if samples or animals were excluded from the analysis. Were the criteria pre-established?<br><br>If sample or data points were omitted from analysis, report if this was due to attrition or intentional exclusion and provide justification.	Not Applicable	
For every figure, are **statistical tests** justified as appropriate? Do the data meet the assumptions of the tests (e.g., normal distribution)? Describe any methods used to assess it. Is there an estimate of variation within each group of data? Is the variance similar between the groups that are being statistically compared?	Yes	Materials and Methods

Sample definition and in-laboratory replication	Information included in the manuscript?	In which section is the information available? (Reagents and Tools Table, Materials and Methods, Figures, Data Availability Section)
In the figure legends: state number of times the experiment was **replicated** in laboratory.	Yes	Figure legends
In the figure legends: define whether data describe **technical or biological replicates**.	Yes	Figure legends

Ethics

Ethics	Information included in the manuscript?	In which section is the information available? (Reagents and Tools Table, Materials and Methods, Figures, Data Availability Section)
Studies involving **human participants**: State details of **authority granting ethics approval** (IRB or equivalent committee(s), provide reference number for approval.	Not Applicable	
Studies involving **human participants**: Include a statement confirming that **informed consent** was obtained from all subjects and that the experiments conformed to the principles set out in the WMA Declaration of Helsinki and the Department of Health and Human Services Belmont Report.	Not Applicable	
Studies involving **human participants:** For publication of **patient photos**, include a statement confirming that consent to publish was obtained.	Not Applicable	
Studies involving experimental **animals**: State details of **authority granting ethics approva**l (IRB or equivalent committee(s), provide reference number for approval. Include a statement of compliance with ethical regulations.	Not Applicable	
Studies involving **specimen and field samples:** State if relevant **permits** obtained, provide details of authority approving study; if none were required, explain why.	Not Applicable	

Dual Use Research of Concern (DURC)	Information included in the manuscript?	In which section is the information available? (Reagents and Tools Table, Materials and Methods, Figures, Data Availability Section)
Could your study fall under dual use research restrictions? Please check biosecurity documents and list of **select agents and toxins** (CDC): https://www.selectagents.gov/sat/list.htm	Not Applicable	
If you used a select agent, is the security level of the lab appropriate and reported in the manuscript?	Not Applicable	
If a study is subject to dual use research of concern regulations, is the name of the **authority granting approval and reference number** for the regulatory approval provided in the manuscript?	Not Applicable	

Reporting

The MDAR framework recommends adoption of discipline-specific guidelines, established and endorsed through community initiatives. Journals have their own policy about requiring specific guidelines and recommendations to complement MDAR.

Adherence to community standards	Information included in the manuscript?	In which section is the information available? (Reagents and Tools Table, Materials and Methods, Figures, Data Availability Section)
State if relevant guidelines or checklists (e.g., **ICMJE, MIBBI, ARRIVE, PRISMA**) have been followed or provided.	Not Applicable	
For **tumor marker prognostic studies,** we recommend that you follow the **REMARK** reporting guidelines (see link list at top right). See author guidelines, under 'Reporting Guidelines'. Please confirm you have followed these guidelines.	Not Applicable	
For **phase II and III randomized controlled trials**, please refer to the **CONSORT** flow diagram (see link list at top right) and submit the CONSORT checklist (see link list at top right) with your submission. See author guidelines, under 'Reporting Guidelines'. Please confirm you have submitted this list.	Not Applicable	

Data Availability

Data availability	Information included in the manuscript?	In which section is the information available? (Reagents and Tools Table, Materials and Methods, Figures, Data Availability Section)
Have **primary datasets** been deposited according to the journal's guidelines (see 'Data Deposition' section) and the respective accession numbers provided in the Data Availability Section?	Not Applicable	
Were **human clinical and genomic datasets** deposited in a public access-controlled repository in accordance to ethical obligations to the patients and to the applicable consent agreement?	Not Applicable	
Are **computational models** that are central and integral to a study available without restrictions in a machine-readable form? Were the relevant accession numbers or links provided?	Not Applicable	
If publicly available data were reused, provide the respective **data citations in the reference list**.	Yes	We will include to the final version reference of RNAseq datasets that were deposited in 2019 and reused here
